# Synthesis and evaluation of new chalcones and oximes as anticancer agents†

**DOI:** 10.1039/d2ra01198k

**Published:** 2022-04-01

**Authors:** Syed Nasir Abbas Bukhari

**Affiliations:** Department of Pharmaceutical Chemistry, College of Pharmacy, Jouf University Sakaka Aljouf 72388 Saudi Arabia sbukhari@ju.edu.sa snab_hussaini@yahoo.com +96 6565738896

## Abstract

Complex illnesses, such as cancer, are often caused by many disorders, gene mutations, or pathways. Biological pathways play a significant part in the development of these diseases. Multi-target directed ligands (MTDLs) have been used by medicinal chemists recently in an effort to find single molecules that can affect many targets concurrently. In this work, several chalcones containing the ligustrazine moiety were synthesized and tested for their *in vitro* anticancer activity and several cancer markers, including EGFR, BRAF^V600E^, c-Met, and tubulin polymerization, in order to uncover multitarget bioactive compounds. In assays using multiple cancer cell lines, the majority of the compounds examined showed strong anticancer activity against them. To synthesize oximes, all of the chalcones were used as precursors. The IC_50_ values of two compounds (11g and 11e) were found to be 0.87, 0.28, 2.43, 1.04 μM and 11d, 1.47, 0.79, 3.8, 1.63 μM respectively, against A-375, MCF-7, HT-29 and H-460 cell lines. These IC_50_ values revealed an excellent antiproliferative activity compared to those of the positive control foretinib, (IC_50_ = 1.9, 1.15, 3.97, and 2.86 μM). Careful examination of their structure and configuration revealed that both compounds had an oxime functional group with *z* configuration, in place of carbonyl functional group, along with a 2-phenyl thiophenyl moiety with or without a bromo group at position-5. The possible binding pattern was implied by docking simulation, inferring the possibility of introducing interactions with the nearby tubulin chain. Since the novel structural trial has been conducted with a detailed structure activity relationship discussion, this work might stimulate new ideas in further modification of multitarget anti-cancer agents and therapeutic approaches.

## Abstract 2

Discovery of multitarget anticancer agents by modifications of natural compound.

## Graphical abstract

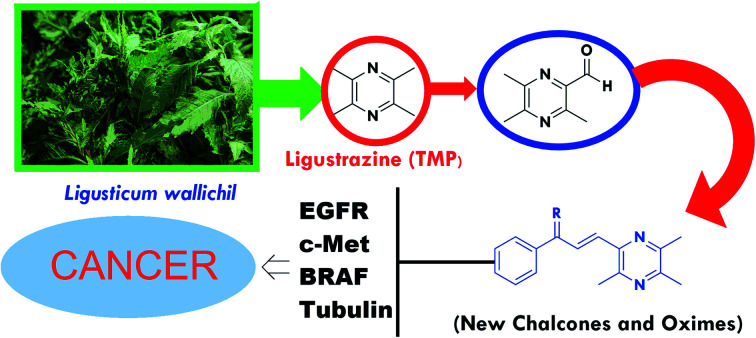

## 1. Introduction

Malignant neoplasm is the medical term for cancer. In this case, the proliferation of cells is aberrant and uncontrolled, resulting in metastasis. This century, cancer is predicted to overtake cardiovascular disease as the major cause of early mortality in most nations.^1^ Premature deaths from cardiovascular disease and cancer were examined in 20 countries for the period 2000–2019 to see if they were on track to reach Sustainable Development Goal (SDG) 3.4, which calls for an overall decrease in noncommunicable disease-related fatalities by one-third by 2030. With regard to the control of cardiovascular disease compared to cancer, national progress was very varied and appeared to be more visible in high-income nations compared to middle-income countries.^2^

Biological activities are common in oximes derived from nature. Because oximes are biosynthetic intermediates for other metabolites and their concentration is generally low, their inclusion in the biological sample may be overlooked.^3^ As an example, oxidation or reduction can biotransform the oxime moiety. The capacity of oximes to bind with metals is their most valuable characteristic, and this makes them ideal candidates for use as metalloenzyme inhibitors as medicinal agents.^4^ A fusion technique can lead to the formation of oximes. Reactive C=O groups and electron pair donors are also hallmarks of oximes in biological compounds.^5^ The cytotoxicity of oximes should be taken into account when considering their use as medicines. Some therapies may have adverse effects since oximes can be cytotoxic, however oximes can also be used as possible anticancer drugs because of their ability to kill cancer cells.^4^ Many oximes have previously been found to be useful in the treatment of a variety of ailments. We've previously identified oximes that are extremely effective at halting the proliferation of cancer cells.^6,7^

On the other hand, chalcone is the preferred scaffold and one of the key core moieties in a large number of naturally occurring pharmacologically active molecules, and has garnered considerable study focus in recent years.^8–16^ We have discovered a huge number of chalcones as multifunctional bioactive agents over the previous decade.^14,17–19^ Thus, in this work, novel chalcones were developed and synthesized utilizing an aldehyde derived from ligustrazine and a range of ketones. We have also extensively reported on ligustrazine-based compounds in recent years due to their diverse biological properties.^20–22^ In the second step, novel chalcones were used as starting materials for the synthesis of oximes, and all newly synthesized compounds were evaluated against various types of cancer cells. The most active compounds were then subjected to anticancer mechanistic studies, and some compounds were also sculpted for binding modes.

For anticancer mechanistic studies inhibition activities of most active compounds towards epidermal growth factor receptor (EGFR), BRAF^V600E^, c-mesenchymal–epithelial transition factor (c-Met) and tubulin polymerization were evaluated. Because overexpression of EGFR is detected in a large percentage of malignancies, targeting EGFR and its downstream signaling cascades is considered a sensible and beneficial approach in cancer therapy.^23^ When cells are growing and developing, the BRAF gene is frequently implicated. Several human cancers include the BRAF (V600E) mutation, which causes an increase in cell proliferation by altering the ERK/MAPK signaling cascade.^24^ While c-Met inhibitors have demonstrated potential anti-tumor effectiveness in pre-clinical and early phase clinical studies in a variety of tumor types, but the majority of phase III trials with these agents have had poor findings.^25^ So, an ideal method for combating cancer is the development of small molecules that exhibit multitarget and multifunctional behaviour and this research is an attempt to discover such multifunctional molecules.

## 2. Results and discussion

### 2.1. Chemistry

**Scheme 1:**
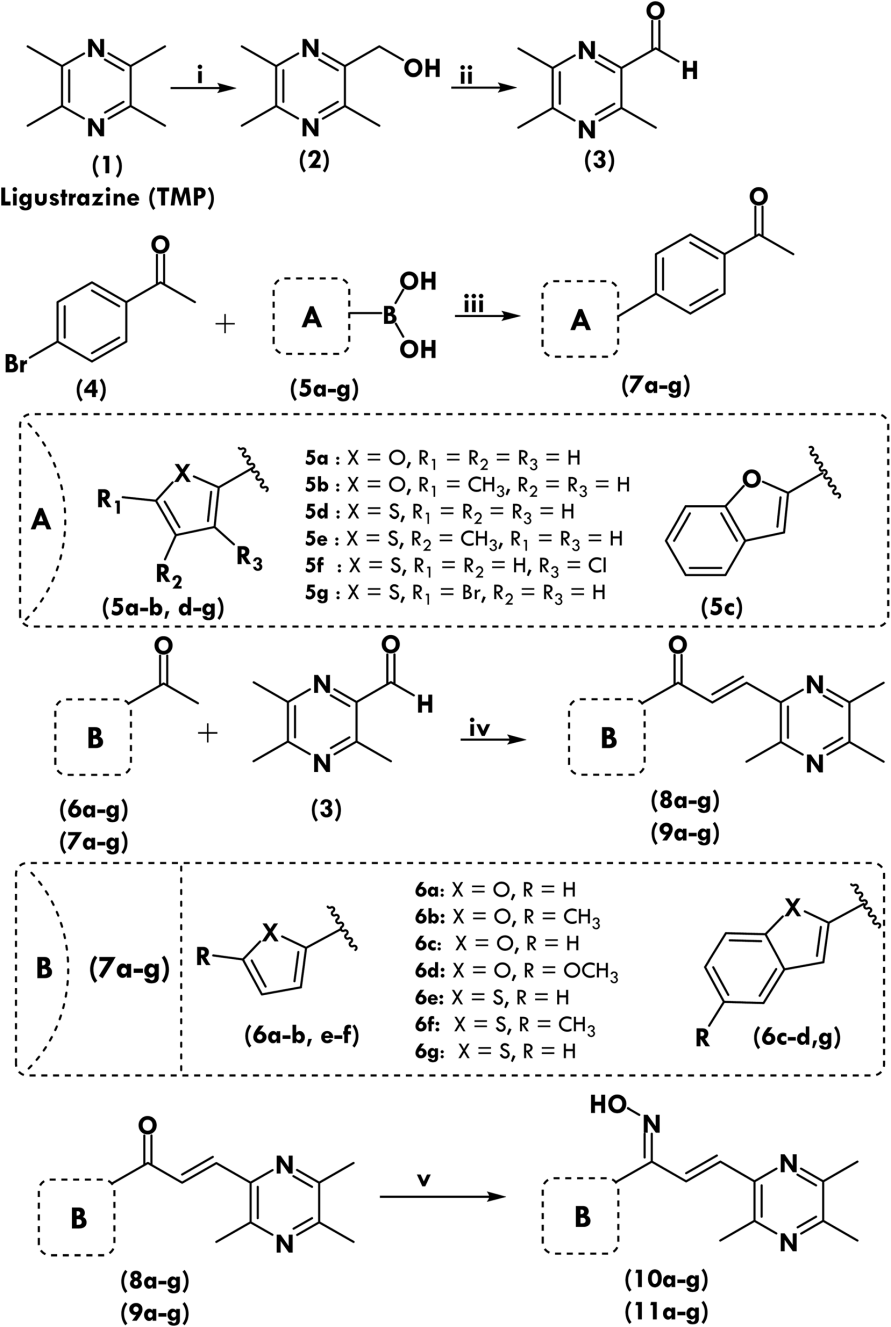
Synthesis scheme of ligustrazine aldehyde (3), modified ketones (7a–g) and new chalcone (8a–g & 9a–g)/oxime derivatives (10a–g & 11a–g). Reagents and conditions: (i-a) 30% H_2_O_2_, acetic acid, 70 °C, 8 h; (i-b) acetic anhydride, reflux, 2 h; (i-c) 20% NaOH; (ii) IBX, DMSO, room temperature, 0.5 h; (iii) K_2_CO_3_, Pd(PPh_3_)_4_, DME, 90 °C, overnight; (iv) NaOH, EtOH, room temperature; 1–6 h; (v) NH_2_OH·HCl, pyridine, ethanol, anhyd., reflux, 6–8 h.

Scheme 1 depicts the methods used to synthesize the target compounds. Ligustrazine based aldehyde, chalcone derivatives and oximes were synthesized as previously described, with minor alterations as we previously reported.^26^ Novel ketones were designed and synthesized as shown in Scheme 1. Chalcone, (1,3-diaryl-2-propen-1-ones) was used as precursor to synthesize new derivatives (8a–8g, 9a–9g, 10a–10g and 11a–11g). The phenyl group of chalcone at position-3 was replaced with 3,5,6-trimethylpyrazine and the phenyl group at position-1 was replaced with five-membered heterocyclic moieties, the carbonyl group was also replaced with oxime group to investigate SAR relationship and to prepare potent derivatives towards antiproliferative activity. The experimental part contains data on the characterisation of all synthesized compounds, including melting point (MP), ^1^H NMR, ^13^C NMR, HRMS, and microanalysis (CHNS). All chemicals have a purity level of at least 95%.

### 2.2. *In vitro* cytotoxicity of target compounds in normal human umbilical vein endothelial cells (HUVECs)

To see how harmful the target compounds were to normal cells, Huvec cells were chosen and treated with all of the compounds at the same time and in the same way as previously reported.^6,27^ A lot of the compounds in Table 1 had an IC_50_ of more than 100 μM. The *in vitro* therapeutic index (IVTI) was then calculated. It was determined by comparing the cytotoxicity activity (IC_50_) of compounds against MCF-7 cells, which were the most sensitive to the test compounds, to that of compounds against Huvec cells. IVTI was above 7 for all compounds. This data suggests that our compounds are safe for potential use as drug candidates.

**Table 1:** The *in vitro* cell proliferation activities (IC_50_ (μM) of new ligustrazine containing chalcone derivatives and chalcone based oxime derivatives against human normal cell line Huvec

Chalcone derivatives			Oxime derivatives		
Compound	Huvec	IVTI^a^	Compound	Huvec	IVTI^a^
8a	>100	19.72	10a	>100	21.69
8b	>100	9.20	10b	94 ± 1.91	25.47
8c	>100	7.91	10c	>100	16.29
8d	>100	12.80	10d	91 ± 1.28	28.26
8e	>100	11.01	10e	92 ± 2.02	38.33
8f	>100	11.83	10f	84 ± 0.98	98.82
8g	>100	8.20	10g	93 ± 1.81	17.58
9a	>100	10.58	11a	91 ± 0.97	29.26
9b	>100	8.11	11b	94 ± 1.29	15.82
9c	>100	7.25	11c	>100	12.36
9d	>100	12.17	11d	88 ± 1.76	111.39
9e	>100	10.79	11e	92 ± 2.03	33.82
9f	>100	10.75	11f	93 ± 1.45	33.70
9g	>100	12.20	11g	88 ± 1.82	314.29

a *In vitro* therapeutic index (Huvec IC_50_/MCF-7 IC_50_).

### 2.3. Antiproliferative activity and development of structure–activity relationship (SAR)

All the newly synthesized chalcone derivatives were examined for their *in vitro* antiproliferative inhibitory activity using, A-375, MCF-7, A-549, HT-29, and H-460 human cancer cell lines. Cisplatin and foretinib were used as a positive control. Inhibition activities were reported as concentrations in micromole (μM) producing 50% inhibition (IC_50_ μM). The IC_50_ values are the average of at least three independent experiments and are presented in Tables 2 and 3.

**Table 2:** Inhibitory effects of synthetic chalcone derivatives on different types of cancer cells

Comp.	Antiproliferative activity IC_50_ ± SD (μM)				
	A375	MCF-7	A-549	HT-29	H-460
8a	7.90 ± 0.94	5.07 ± 0.37	5.11 ± 0.24	9.58 ± 0.32	5.41 ± 0.86
8b	15.36 ± 0.96	10.87 ± 0.55	5.83 ± 0.54	28.35 ± 1.20	5.15 ± 0.81
8c	18.53 ± 1.04	12.64 ± 0.40	6.68 ± 0.06	30.39 ± 0.91	8.96 ± 1.10
8d	5.16 ± 1.23	7.81 ± 0.48	5.77 ± 0.28	8.18 ± 1.12	5.78 ± 1.12
8e	12.88 ± 1.62	9.08 ± 0.73	5.27 ± 1.00	24.36 ± 1.11	8.89 ± 0.17
8f	11.06 ± 1.52	8.45 ± 0.52	5.24 ± 0.57	23.44 ± 1.10	8.73 ± 0.69
8g	16.89 ± 0.83	12.20 ± 0.56	6.23 ± 0.70	29.64 ± 0.40	6.07 ± 0.53
9a	14.75 ± 1.49	9.45 ± 1.15	5.69 ± 0.23	27.52 ± 1.38	3.60 ± 1.07
9b	18.38 ± 1.37	12.33 ± 0.03	6.37 ± 1.20	30.21 ± 1.14	7.42 ± 0.90
9c	18.97 ± 1.90	13.80 ± 0.13	6.82 ± 0.59	30.69 ± 0.19	9.81 ± 0.91
9d	7.86 ± 0.99	8.22 ± 0.04	5.16 ± 0.47	11.67 ± 0.96	8.72 ± 0.28
9e	3.68 ± 1.01	9.27 ± 0.21	5.39 ± 0.82	6.54 ± 0.33	2.72 ± 1.19
9f	14.72 ± 1.61	9.30 ± 1.16	5.55 ± 0.49	27.24 ± 0.80	3.40 ± 1.05
9g	9.75 ± 1.30	8.20 ± 0.78	4.84 ± 0.69	19.98 ± 0.13	8.05 ± 1.23
Cisplatin	9.46 ± 0.14	12.25 ± 0.95	5.12 ± 0.23	25.4 ± 0.23	6.84 ± 0.92
Foretinib	1.9 ± 0.02	1.18 ± 0.06	1.15 ± 0.05	3.97 ± 0.05	2.86 ± 0.04

**Table 3:** Inhibitory effects of synthetic oxime derivatives on different types of cancer cells

Comp.	Antiproliferative activity IC_50_ ± SD (μM)				
	A375	MCF-7	A-549	HT-29	H-460
10a	6.93 ± 1.87	4.61 ± 1.14	3.59 ± 0.51	4.47 ± 0.49	6.31 ± 0.09
10b	6.55 ± 0.93	3.69 ± 1.00	2.83 ± 0.74	14.13 ± 0.03	4.99 ± 1.25
10c	9.01 ± 1.56	6.14 ± 0.28	4.58 ± 1.06	18.12 ± 0.99	6.74 ± 1.08
10d	4.15 ± 1.48	3.22 ± 0.49	2.74 ± 0.53	7.29 ± 1.31	3.81 ± 0.78
10e	2.62 ± 1.82	2.40 ± 0.50	1.80 ± 0.61	8.80 ± 0.73	2.52 ± 0.71
10f	2.43 ± 1.51	0.85 ± 0.38	1.42 ± 0.42	7.83 ± 1.23	1.83 ± 0.68
10g	7.57 ± 1.39	5.29 ± 0.42	3.61 ± 0.67	17.34 ± 0.89	6.68 ± 0.59
11a	4.97 ± 0.72	3.11 ± 0.30	2.71 ± 0.90	9.10 ± 0.21	3.87 ± 1.01
11b	8.63 ± 1.00	5.94 ± 0.86	4.39 ± 0.26	18.09 ± 0.29	6.71 ± 0.36
11c	9.74 ± 1.34	8.09 ± 0.36	4.80 ± 1.18	19.08 ± 0.58	7.37 ± 0.72
11d	1.47 ± 1.36	0.79 ± 0.72	1.32 ± 0.50	3.80 ± 0.11	1.63 ± 0.43
11e	2.69 ± 0.98	2.72 ± 0.80	1.18 ± 0.86	7.35 ± 0.59	2.76 ± 0.87
11f	2.42 ± 1.38	2.76 ± 0.15	1.48 ± 0.48	6.03 ± 1.17	2.17 ± 1.21
11g	0.87 ± 1.60	0.28 ± 0.70	1.25 ± 0.62	2.43 ± 1.24	1.04 ± 0.05
Cisplatin	9.46 ± 0.14	12.25 ± 0.95	5.12 ± 0.23	25.4 ± 0.23	6.84 ± 0.92
Foretinib	1.9 ± 0.02	1.18 ± 0.06	1.15 ± 0.05	3.97 ± 0.05	2.86 ± 0.04

As from antiproliferative assay results shown in Table 3, the IC_50_ values of two compounds (11d and 11g) are 1.47, 0.79, 3.8, 1.63 μM and 0.87, 0.28, 2.43, 1.04 μM respectively against four different A-375, MCF-7, HT-29 and H-460 cell lines. These IC_50_ values showed an excellent antiproliferative activity than that of the positive control foretinib (IC_50_ = 1.9, 1.18, 3.97, and 2.86 μM) but these compounds were found to have high IC_50_ values (1.32 and 1.25 μM) than foretinib (IC_50_, 1.15 μM) for adenocarcinomic human alveolar basal epithelial cells A-549. Careful examination of their structure and configuration revealed that both compounds had oxime functional group with *z* configuration, in place of carbonyl functional group along with 2-phenyl thiophenyl moiety with and without bromo group at position-5.

#### 2.3.1. Human melanoma A-375 cell line

The *in vitro* antiproliferative inhibitory activity showed that only two compounds (11g, 11d) were more active (IC_50_ 0.87 and 1.47 μM) than foretinib (IC_50_ 1.9 μM). Both compounds had oxime group with *z* configuration which was linked with 2-phenyl thiophene moiety. It was observed that the bromo group at position-5 of thiophene moiety was responsible for higher activity of 11g than 11d compounds. To evaluate the presence of 2-phenyl thiophene moiety and oxime group, another molecule with the same moiety but linked with the ketone group was prepared (9d). The IC_50_ value was reduced from 1.47 to 7.86 μM. This result showed that phenyl moiety had a more positive effect on the activity when attached with oxime moiety. Total seventeen compounds (including 11g and 11d) showed better activity (IC_50_ ranging between 0.87 to 9.01 μM) than cisplatin (IC_50_ 9.46 μM). If we compared the structure of these compounds with more active compounds (11g and 11d) then it can be observed that when the chloro group was inducted at position-3 of thiophene moiety, the activity reduced (IC_50_ 2.42 μM) than compound 11g. This result showed that the bromo group at position-5 had more effectiveness than the chloro group at position-3. Then thiophene moiety was changed by removing the phenyl group (10e) the activity was reduced (IC_50_ 2.62 μM). This showed that the presence of the phenyl group was essential to get improved activity. To enhance the activity the methyl group at position-5 was induced (10f) which increased little bit activity (2.43 μM) but the methyl group at position-4 (11e) did not favour the activity (2.69 μM) even in the presence of phenyl group. The same moieties of 11e were tested in compound (9e) had ketone group, the reduced activity was observed (IC_50_ 3.68 μM). The possible reason for this reduced activity can be linked with methyl at position-4 of thiophene moiety irrespective of oxime and ketone group in compounds. Finally, thiophene was fused with a benzo group (10g), the result showed a significant negative effect on the activity (IC_50_ 7.57 μM). To evaluate the relationship of thiophene towards activity, we changed the moiety with the furan group. The comparative results (11d and 11a; 9e and 11b; 8g and 8c; 8f and 8b) showed the thiophene moiety had a positive effect than furan moiety. It was also observed that most of the compounds containing the oxime group had more activity compared to the same compounds containing the ketone group.

#### 2.3.2. Breast cancer cell line MCF-7

The *In vitro* antiproliferative inhibitory activity towards MCF-7 lines showed that only three compounds (11g, 11d, and 10f) were more active (IC_50_ 0.28, 0.79, and 0.85 μM) than foretinib (IC_50_ 1.18 μM). These compounds had an oxime group with *z* configuration which was linked with 2-phenyl thiophene moiety. It was observed that the bromo group at position-5 of thiophene moiety was responsible for higher activity of 11g than 11d compounds. The compound 10f had methyl group at position-5 but phenyl group was not attached, its lower activity (IC_50_ 0.85 μM) showed that bromo group at position-5 and phenyl group at position-2 of thiophene moiety was essential structural feature towards better activity.

To evaluate the presence of 2-phenyl thiophene moiety and oxime group, another molecule with the same moiety but linked with the ketone group was prepared (9d). The IC_50_ value was reduced from 0.79 to 8.22 μM. This result showed that phenyl moiety had a positive effect with oxime moiety only.

Similarly, to evaluate the effect of 5-bromo-2-phenyl thiophene moiety linked with oxime moiety, another compound (9g) with the same moieties was prepared. The only difference between them was the presence of the ketone group instead of the oxime group. The results showed a sudden decrease in the activity and the IC_50_ value changed from 0.28 μM to 8.2 μM). The chloro group was inducted at position-3 of thiophene moiety (11f), the activity reduced (IC_50_ 2.76 μM) compared to compound 11g. This result showed that the bromo group at position-5 had more effectiveness than the chloro group at position-3.

Total, twenty-five compounds (including 11g, 11d, and 10f) showed better activity (IC_50_ ranging between 0.28 to 12.2 μM) than cisplatin (IC_50_ 12.25 μM). If we compared the structure of these compounds with more active compounds (11g, 11d, and 11f) then it can be observed that when the methyl group at position-5 (10f) was replaced with hydrogen (10e) the activity decreased (IC_50_ 2.4 μM), further another methyl group at position-4 was induced (11e) but no increased of activity was observed rather activity dropped even in the presence of phenyl group (IC_50_ 2.72 μM). Finally, the thiophene group was fused with the benzo group, this led to a high reduction in the activity (IC_50_ 5.29 μM). To evaluate the relationship of thiophene towards activity, we changed the moiety with the furan group. The comparative results (11d and 11a; 10f and 10b; 10e and 10a) showed that the thiophene moiety had a positive effect than furan moiety. It was also observed that most of the compounds containing the oxime group had more activity compared to the same compounds containing the ketone group.

#### 2.3.3. Adenocarcinomic human alveolar basal epithelial cells A-549

Among all prepared compounds, none of the compounds was found to be more active towards A-549 cancer cell lines than foretinib (IC_50_ 1.15 μM). Sixteen compounds (IC_50_ values were ranging between 1.18 to 5.11 μM) showed more activity than cisplatin (IC_50_ 5.12 μM) and twelve compounds were less active. It was observed that active compounds had an oxime group with *z* configuration except for two compounds (9g and 8a). The compounds with less activity were found to be those molecules that had ketone functional groups linked with substituted thiophene or substituted furan moieties. The compound (11e) showed the highest activity (IC_50_ 1.18 μM) towards A-549 lines. The structure analysis of this compound showed the oxime group linked with 4-methyl-2-phenyl thiophene moiety. To investigate SAR, a methyl group and phenyl groups were removed one by one (11d and 10f). The results showed that removal of methyl group reduced the activity (IC_50_ 1.32 μM) and removal of phenyl group reduced the activity (IC_50_ 1.42 μM). Then both groups were removed from the compound (10e) also reduced the activity ((IC_50_ 1.8 μM). The results showed that both of these groups are essential for activity. To investigate the effect of the oxime group, the compound (9e) was prepared to have a ketone group. The activity of the compound reduced significantly (IC_50_ 5.39 μM). This result proved that the oxime group had a positive effect on the activity than the same molecular moieties with the ketone functional group. The bromo and chloro functional groups were also evaluated (11g and 11f), both of these functional groups showed reduced activity (1.25 and 1.48 μM) than the compound with a methyl group. To evaluate the relationship of thiophene moiety towards activity, we changed the moiety with the furan group. The comparative results (11d and 11a; 10f and 10b; 10e and 10a) showed that the thiophene moiety had a positive effect than furan moiety. It was also observed that most of the compounds containing the oxime group had more activity compared to the same compounds containing a ketone group.

#### 2.3.4. Human colon cancer cell line HT-29

The *in vitro* antiproliferative inhibitory activity towards HT-29 lines showed that only two compounds (11g and 11d) were more active (IC_50_ 2.43 and 3.8 μM) than foretinib (IC_50_ 3.97 μM). These compounds had an oxime group with *z* configuration which was linked with 2-phenyl thiophene moiety. To relate the higher activity of 11g with the oxime group, a molecule with the same structural features having the ketone group was prepared (9g). The reduced activity of this molecule (IC_50_ 19.98 μM) proved that the oxime group with *z* configuration was essential towards higher activity. The compounds (11g and 11e) had 2-phenyl thiophene moiety linked with the oxime group, to investigate its presence towards activity, another molecule with the same moiety but linked with the ketone group was prepared (9d). The IC_50_ value was reduced from 3.8 to 11.67 μM. This result showed that phenyl moiety had a more positive effect on oxime moiety. Total twenty-one compounds (including 11g and 11d) showed better activity (IC_50_ ranging between 2.43 to 24.36 μM than cisplatin (IC_50_ 25.4 μM). To investigate SAR, the chloro group at position-3 (11f) and the methyl group at position-4 were added (11e). The results of these changes showed a reduced activity (IC_50_ 6.03 and 7.35 μM). This proved that induction of chloro and methyl groups did not favour activity. A reduced activity (IC_50_ 8.8 μM) was also observed when the phenyl group was removed from the prepared molecule (10e). The significantly reduced activity (IC_50_ 8.8 μM) was observed when the benzo group was fused with thiophene moiety (10g). To evaluate the relationship of thiophene moiety towards activity, we changed the moiety with the furan group. The comparative results (11d and 11a; 10f and 10b; 10e and 10a) showed that the thiophene moiety had a positive effect than furan moiety. It was also observed that most of the compounds containing the oxime group had more activity compared to the same compounds containing the ketone group.

The results also showed that out of twenty-eight compounds, seventeen compounds (11g, 11d, 11f, 10f, 10e, 11e, 9e, 10d, 11a, 8d, 10b, 10a, 10g, 9d, 8a, 11b and 10c) were more active towards A-375, MCF-7 and HT-29 lines than cisplatin. The IC_50_ values of these prepared compounds were ranging between 0.87 to 9.01 μM, 0.28 to 6.14 μM, and 2.43 to 18.12 μM respectively). It was observed in the compounds that the majority of them had oxime functional group with *z* configuration in their structures and only four compounds had the carbonyl group in their structures.

Three compounds (11g, 11d and 10f) were found to be more active (IC_50_, 0.28, 0.79, 0.85 μM) towards MCF-7 than foretinib (IC_50_, 1.18 μM). The common structural features in these structures were the presence of oxime group with *z* configuration and substituted thiophene moiety directly or indirectly linked with carbon-containing oxime group. Out of twenty-eight, twenty-five compounds were found to be more active than cisplatin (IC_50_ were ranging between 0.28 to 12.2 μM). Only three compounds (9b, 8c, 9c) were found to be less active (IC_50_, 12.33, 12.64, and 13.8 μM than cisplatin (IC_50_, 12.25 μM). It was observed that these compounds had a ketone group in their structure which was linked with benzofuran or methyl furan directly or with a phenyl group.

Among all prepared compounds, none of the compounds was found to be more active towards A-549, than foretinib (IC_50_ 1.15 μM). Sixteen compounds (IC_50_ values were ranging between 1.18 to 5.11 μM) showed more activity than cisplatin (IC_50_ 5.12 μM) and twelve compounds were less active. It was observed that active compounds had an oxime group with *z* configuration except for two compounds (9g and 8a). The compounds with less activity were found to be those molecules that had ketone functional groups linked with substituted thiophene or substituted furan moieties. Seven compounds (9f, 9a, 8b, 8g, 9b, 8c and 9c) had low efficacy (27.24, 27.52, 28.35, 29.64, 30.21, 30.39 and 30.69 μM) than cisplatin. It was observed that compounds with low efficacy had a ketone group in their structures which was either linked with at position-3 of benzothiophene and 2-phenyl-3-chlorothiophene moieties or with 2-phenyl furan, 5-methyl furan, 2-phenyl-5-methyl furan, benzofuran, and 2-phenyl benzofuran moieties.

#### 2.3.5. Human lung carcinoma cells H-460

The antiproliferative activity results of synthesized compounds towards H-460 cancer cell lines showed that eleven compounds (8b, 8a, 11g, 8c, 8d, 11d, 10f, 11f, 10e, 9e and 11e) were more active (IC_50_ ranging between 0.81 to 2.76 μM) than foretinib (2.86 μM). Among eleven active compounds, six compounds had an oxime group with *z* configuration and five compounds had ketone group in their structures. All oxime-containing compounds were linked with thiophene or substituted thiophene moieties linked at position-3.

The IC_50_ value was 2.52 μM when unsubstituted thiophene moiety was linked with the oxime group of the prepared molecule (10e). When thiophene moiety was fused with a benzo group (10g), the activity dropped from 2.52 to 6.68 μM and activity increased (IC_50_ 1.83 μM) with the induction of methyl group at position-5 (10f). When the phenyl group was inducted at position-2 of thiophene moiety the molecule (11d) became more active with an IC_50_ value of 1.63 μM. The activity further enhanced (IC_50_ 1.04 μM) with the induction of bromo group at position-5 of 2-phenyl thiophene moiety (11g). The induction of the chloro group (11f) in place of the bromo group dropped the IC_50_ value from 1.04 to 2.17 μM. The induction of methyl group at position-4 of 2-phenyl thiophene moiety (11e) was tested and it was observed that methyl group at position-4 lowered the activity (IC_50_ 2.76 μM). The comparable results were observed for some of the compounds (9e, 11e, 9f and 11f) irrespective of oxime or ketone groups.

The activity concerning furan moiety linked with oxime group was studied and it was observed that the molecule with unsubstituted furan moiety (10a) had lower activity (IC_50_ 6.31 μM) than the molecule (10e), which has unsubstituted thiophene moiety (2.52 μM). From this observation, it can be concluded that thiophene moiety had a more significant effect on antiproliferative activity towards H-460 cell lines. To evaluate complete SAR towards furan moiety, the prepared molecule (10a) was changed using different functional groups.

Firstly, the benzo group was fused with furan moiety (10c), the activity reduced (IC_50_ 6.74 μM). The same results were observed in the case of thiophene moiety. It can be concluded that the fused benzo group may be responsible for the reduced activity. The induction of methyl group at position-5 of furan moiety (10b), enhanced the activity (IC_50_ 4.99 μM). It was observed that, when the methoxy group was induced at position-5 of furan moiety (10d), the activity increased towards H-460 lines (IC_50_ 3.81 μM). The induction of phenyl group at position-2 of furan moiety (11a) also increased the activity (IC_50_ 3.87 μM) but induction of methyl group at position-4 of 2-phenyl furan moiety (11b) reduced the activity (IC_50_ 6.71 μM). The antiproliferative activity towards H-460 cell lines was investigated concerning furan moiety linked with the ketone group (8b, 8a, 8c, 8d and 9a). The unsubstituted furan moiety linked with the ketone group (8a) showed an IC_50_ value of 0.86 μM. When the methyl group was induced at position-5 of furan (8b), the molecule showed maximum activity (IC_50_ 0.81 μM). The induction of benzo and 5-methoxybenzo group at furan group (8c, 8d) dropped the activity (IC_50_ 1.1 and 1.12 μM).

The results showed that out of twenty-eight compounds, twenty-one compounds (8b, 8a, 11g, 8c, 8d, 11d, 10f, 11f, 10e, 9e, 11e, 9f, 9a, 10d, 11a, 10b, 8g, 10a, 10g, 11b and 10c) were more active (IC_50_ ranging between 0.81 to 6.74 μM) towards H-460 than cisplatin (IC_50_ 6.84 μM) and seven compounds (11c, 9b, 9g, 9d, 8f, 8e and 9c) were less active (IC_50_ ranging between 7.37 to 9.81 μM) than cisplatin towards H-460 lines.

The most active compound (8a) had an IC_50_ value of 0.81 μM and the least active compound (10c) had IC_50_ 6.74 μM. The structural examination of these compounds showed the most active compound (8b) had 5-methyl furan moiety which was linked with the ketone functional group of chalcone compound. It was observed that removal of this methyl group (8a) or substitution with benzo (8c) and methoxybenzo group (8d) showed negative activity. A similar pattern of reduced activity was observed when compound (8a) was changed by replacing ketone group with oxime group with *z* configuration and methyl furan moiety with 5-methoxybenzo furan (10d), 2-phenyl furan (11a), 5-methyl furan (10b), furan (10a), 5-methyl-2-phenyl furan (11b) and benzofuran (10c) moieties. The seven compounds (11c, 9b, 9g, 9d, 8f, 8e and 9c) had reduced activity (IC_50_ ranging between 5.84 to 9.81 μM) than cisplatin. All these compounds had a ketone functional group in their structure except 11c. The ketone/thiophene group was attached with substituted/unsubstituted moieties of furan and thiophene rings.

### 2.4. Inhibition activity towards EGFR, BRAF^V600E^, c-Met

**Fig. 1:**
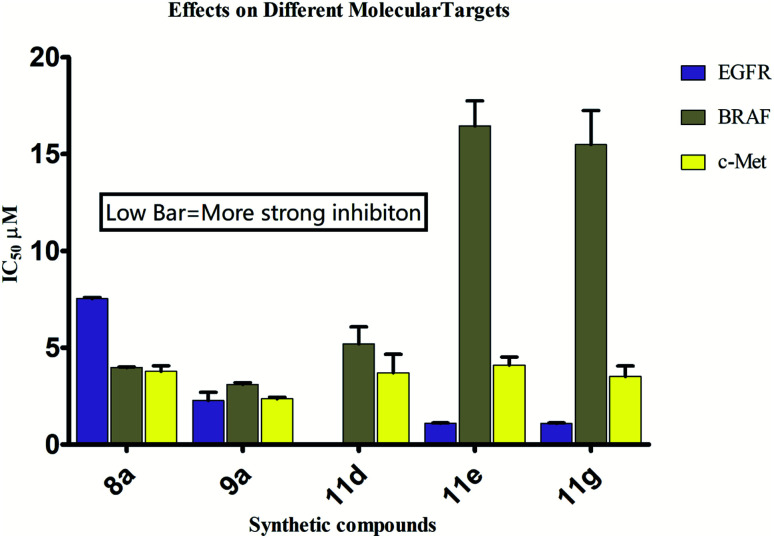
Comparison of inhibitory activities (IC_50_ μM) of compounds on three targets.

The eight more active antiproliferative compounds (8a, 8d, 9a, 9e, 10d, 10e, 10f, 11d, 11e and 11g) were selected to test inhibition activity towards epidermal growth factor receptor (EGFR), BRAF^V600E^ and c-Met. The foretinib was used as a positive control. The results are presented in Table 4 that shows, that the prepared compound (11d) was found to be more active (IC_50_ 0.027 μM) than foretinib (IC_50_ 0.075 μM) towards EGFR inhibition but it was less active (IC_50_ 5.2, 3.71 μM) towards BRAF^V600E^ and c-Met inhibition (IC_50_ 0.09, 0.024 μM). The compound (11g) showed similar activity towards BRAF^V600E^ and c-Met inhibition as that of foretinib but was found to be less active (1.09 μM) towards EGFR (0.075 μM). Comparison of most active compounds of various targets have also been shown in Fig. 1.

### 2.5. Tubulin polymerization assay

Blocking mitosis and cell death are the results of interfering with the microtubule dynamics by inhibiting tubulin polymerization or depolymerization.^28^ Hence tubulin has been identified as a very interesting therapeutic target for the identification of therapeutically effective anticancer drugs, and the principal binding sites on tubulin are the taxane, vinblastine, and colchicine domains.^29^ Most active anticancer molecules were tested for their ability to block tubulin polymerization in order to see if their anticancer activity was a result of their interaction with tubulin. For comparison, colchicine and vinorelbine were utilized. Tubulin polymerization was inhibited by compound 10d, as seen in Table 4, in a manner reminiscent to colchicine. Additionally, the findings of experiment reported in Table 4 suggest that compound 10d was a powerful tubulin polymerization inhibitor with an IC_50_ of 0.41 μM compared to conventional colchicine, which indicates that compound 10d was more effective than normal colchicine. Compound 10d growth suppression appears to be linked to tubulin polymerization inhibition, and these findings support the idea that compound 10d is a robust tubulin assembly inhibitor.

**Table 4:** Effects of selected synthetic compounds on tubulin polymerization, EGFR, BRAF^V600E^ and c-Met

Comp.	EGFR inhibition, IC_50_ ± SD (μM)	BRAF inhibition, IC_50_ ± SD (μM)	c-Met inhibition, IC_50_ ± SD (μM)	Tubulin polymerization, IC_50_ ± SD (μM)
8a	7.54 ± 0.05	3.97 ± 0.04	3.78 ± 0.29	3.71 ± 0.82
8d	19.65 ± 1.07	15.04 ± 1.02	3.61 ± 0.94	1.10 ± 0.07
9a	2.28 ± 0.42	3.11 ± 0.09	2.36 ± 0.09	4.42 ± 1.22
9e	2.61 ± 2.94	10.30 ± 1.07	6.60 ± 0.76	5.61 ± 0.91
10d	22.5 ± 1.52	7.39 ± 0.98	1.47 ± 0.09	0.41 ± 0.02
10e	12.9 ± 0.58	9.31 ± 1.82	5.64 ± 0.95	1.51 ± 0.24
10f	14.2 ± 0.74	14.94 ± 1.20	3.86 ± 0.43	2.21 ± 0.52
11d	0.027 ± 0.004	5.20 ± 0.87	3.71 ± 0.95	1.12 ± 0.04
11e	1.098 ± 0.021	16.45 ± 1.29	4.10 ± 0.42	1.72 ± 0.92
11g	1.09 ± 0.028	15.48 ± 1.76	3.51 ± 0.55	1.51 ± 0.42
Foretinib	0.075 ± 0.005	0.09 ± 0.02	0.024 ± 0.002	—
Colchicine	—	—	—	0.55 ± 0.02
Vinorelbine	—	—	—	31.7 ± 1.5

### 2.6. Molecular studies

**Fig. 2:**
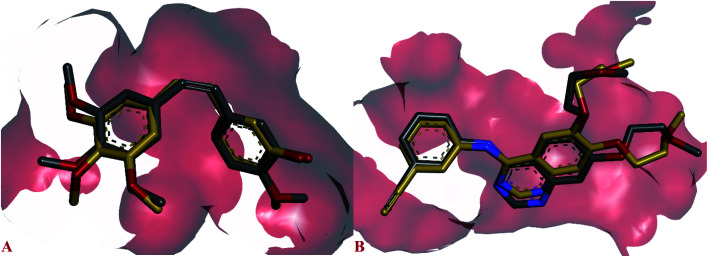
Overlay of re-docked (golden) and crystallographic (grey) conformations of (A) erlotinib in EGFR tyrosine kinase (1M17) and (B) CA4 in and tubulin (5LYJ) respectively.

The possible binding modes of most active compounds were investigated by performing molecular docking studies using Autodock Vina software. We selected 11d and 11g against EGFR tyrosine kinase, 8d and 10d against tubulin protein respectively. For tubulin protein the colchicine binding site was selected as the active site. The docking parameters in both cases were authenticated by re-docking the erlotinib in the active site of EGFR tyrosine kinase and combretastatin A4 (CA4) in the colchicine active site of tubulin. The re-docked (golden) erlotinib and CA4 were found to make interactions in nearly the similar manner as their corresponding crystallographic conformation (grey) approving the docking parameters to be satisfactory (Fig. 2).

**Fig. 3:**
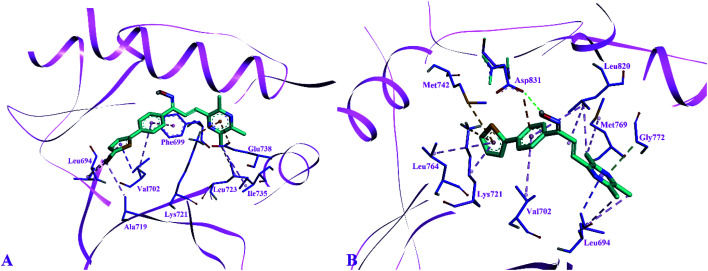
Docked pose of most active inhibitors 11g and 11d with the active site amino acids of EGFR tyrosine kinase, inhibitors shown in cyan color and amino acids of active site in violet color. (A) Binding pose compound 11g; (B) binding pose of compound 11d.

The side chain amino acids of the active site of EGFR tyrosine kinase and the interactions of most active compounds 11d and 11g are shown in Fig. 3. The docking analysis of compounds 11d and 11g in the active site of EGFR tyrosine kinase displayed significant interactions. The top active synthesized compound 11d (IC_50_ = 0.027 ± 0.004 μM) revealed that hydroxyl group of oximes formed a hydrogen bond with the side chain carboxylate group of Asp831 (3.39 Å). Other intermolecular interactions stabilizing the complex (−8.5 kcal mol^−1^) were formed between the three aromatic rings of compound 11d and side chain residues of active site. The thiophen ring formed π–sulphur interaction with Met742, π–cation interaction with side chain Lys721 and π–alkyl interactions with Leu764 and Lys721 respectively. The aromatic phenyl ring was involved in making hydrophobic interactions *i.e.*, π–alkyl and π–anion interactions with Val702 and Asp831 respectively. Sidewise, the side chain Leu820 made π–alkyl interaction with different parts of compound 11d including the phenyl ring, the alkene group and pyrazine ring. The pyrazine ring was found in forming alkyl–alkyl and π–sigma interactions with side chain Leu694, Gly772 and Met769 respectively.

Binding modes compound 11g (IC_50_ = 1.09 ± 0.028 μM, −8.7 kcal mol^−1^), second highest active compound showed that this compound was not making any hydrogen bond interaction as found in compound 11d. The thiophene ring of compound 11g formed alkyl–alkyl, π–alkyl and π–sigma interaction with the side chain Ala719, Leu694 and Val702 respectively. Whereas, the phenyl ring of compound 11g was involved in making π–π stacked interaction with Phe699 and π–alkyl interaction with Val702. Furthermore, the pyrazine ring was involved in forming π–anion interaction with Glu738, π–cation; π–donor interaction with Lys721 and alkyl–alkyl interaction with Ile735 and Leu723 respectively.

**Fig. 4:**
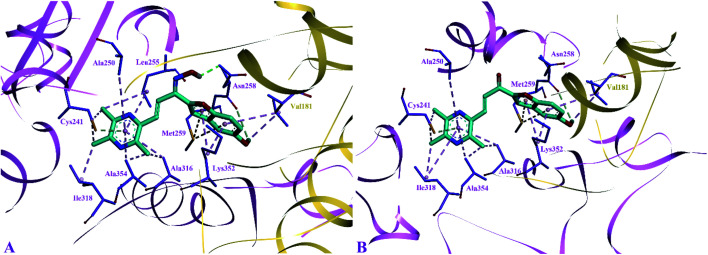
Docked pose of most active inhibitors 10d and 8d with the amino acids of colchicine binding site of tubulin. Inhibitors shown in cyan color and amino acids in violet (β-tubulin) and golden (α-tubulin) color. (A) Binding pose compound 10d; (B) binding pose of compound 8d.

The side chain amino acids of the colchicine binding site of tubulin and the interactions of most active compounds 8d and 10d are shown in Fig. 4. The colchicine binding site consists of both α and β subunits of the tubulin protein. The most active compound 10d (IC_50_ = 0.41 ± 0.02 μM, −9.9 kcal mol^−1^) gets stabilized in the active site of tubulin by hydrogen bond interaction and hydrophobic interactions. A hydrogen bond interaction was found between the amide group of βAsn258 and hydroxyl group of ligand (2.33 Å). Furthermore, the pyrazine group was involved in generating alkyl–alkyl, π–alkyl, and π–donor; π–sulphur interactions with the side chain amino acids *viz* βIle318, βAla354, βAla316, βAla250 and βLeu255, and βCys241. The benzofuran part of the compound 10d showed two π–sulphur interactions with side chain βMet259 and π–alkyl interactions with side chain βLys325 and αVal181.

The predicted binding poses of compound 8d (IC_50_ = 1.10 ± 0.07 μM, −9.4 kcal mol^−1^) showed similar interactions as portrayed by compound 10d except for the hydrogen bond interaction. As depicted in Fig. 3, the pyrazine ring of compound 8d formed alkyl–alkyl, π–alkyl, and π–donor; π–sulphur interactions with βAla250, βAla354, βAla316, βIle318 and βCys241. Whereas the benzofuran ring was involved in forming alkyl–alkyl, π–alkyl and π–sulphur interactions with αVal181, βLys352, and βMet259.

## 3. Conclusion

Cancer therapy still necessitates the discovery of novel drugs. In some cancer cells, chalcones and oximes have anticancer properties. In summary, a class of novel substituted chalcone derivatives were designed and synthesized. The phenyl group of chalcone at position-3 was replaced with 3,5,6-trimethylpyrazine and the phenyl group at position-1 was replaced with five-membered heterocyclic moieties, the carbonyl group was also replaced with oxime group to investigate SAR relationship and to prepare potent derivatives towards antiproliferative activity. All the newly synthesized chalcone derivatives were examined for their *in vitro* antiproliferative inhibitory activity using, A-375, MCF-7, A-549, HT-29, and H-460 human cancer cell lines. The eight more active antiproliferative were selected to test inhibition activity towards EGFR, BRAFV600E and c-Met. Molecular analysis indicates that the compound 11d was found to be more active than foretinib towards EGFR inhibition. The compound 11g showed similar activity towards BRAF^V600E^ and c-Met inhibition as that of foretinib. Tubulin polymerization was inhibited by compound 10d in a manner reminiscent to colchicine. Several of the compounds presented appear to be interesting treatment candidates, targeting multiple pathways in cancer cells and appearing to be extremely promising in the field of cancer therapy. Furthermore, it might serve as a vital motivation in the hunt for novel effective multitarget medicines for cancer metastasis. Further research on structural optimization and biological activities of these most active derivatives is still being performed in our laboratory and will be reported in the future. Further study will involve the inclusion of highly effective functional groups at optimal influence points, as well as *in vivo* evaluation of the same compounds, in order to improve and sustain the efficacy of test compounds.

## 4. Experimental section

### 4.1. Chemistry general information

Analytical grade reagents and chemicals were provided by Acros Organics, Merck, and Sigma-Aldrich. In order to get ^13^C and ^1^H NMR data, the JEOL ECP spectrometer (500 MHz) was employed. The MicroTOF-Q mass spectrometer was used to capture high-resolution mass spectra (HRMS) (Bruker). In order to collect the microanalysis results, the Fison EA 1108 elemental analyzer was employed. Flash column chromatography was done using Merck's silica gel 60 (230–400 mesh) whereas thin layer chromatography (TLC) was carried out using pre-coated silica plates (kiesel gel 60 F254, BDH). Vanillin stain or UV light were used to evaluate the compounds after they had been scorched on a hotplate (254 nm).

### 4.2. Synthesis of ligustrazine containing chalcones and oximes

Direct coupling was used to synthesis new chalcones compounds (8a–g and 9a–g) (Scheme 1).^12^ The Claisen–Schmidt condensation process was used to synthesis novel chalcones by reacting various ketones with ligustrazine-based aromatic aldehyde at a molar ratio of 1 : 1. We first synthesized modified substituted ketones 7a–g and an aldehyde on the basis of the ligustrazine compound 3 in order to make novel chalcones, and then we reacted those aldehydes with ketones in the second step. We've already documented the synthesis's step-by-step process.^12,30^ The key steps in the synthesis of chalcones and their oxime derivatives may be seen in Scheme 1. After taking 15 mL of ethanol and dissolving aromatic aldehyde (10 mmol, 1 equivalent) and specific ketones (10 mmol, 1 equivalent) for 2–3 minutes at 5 °C, the mixture was centrifuged to remove any solids. Add 40 percent NaOH solution in ethanol dropwise to the aforementioned solution and agitate the mixture for 1–24 hours at 27 °C. The change in hue and precipitation in the reaction mixture indicated that the product had formed. For the synthesis of modified ketones (7a–g) the 4-bromoacetophenone (4) (200 mg, 1.01 mmol), various types of substituted boronic acid derivatives (5a–g) (3 mmol), potassium carbonate (277 mg, 2.01 mmol), and tetrakis(triphenylphosphine)palladium (116 mg, 0.10 mmol) were dissolved in DME (5 mL) under N_2_ atmosphere. The solution was stirred at 90 °C overnight, then the reaction solution was diluted with water and extracted with ethyl acetate. The organic layers were combined, concentrated, and purified by column chromatography to give 7a–g as solid. Acidified ice was used to stop the reaction after it was complete, and TLC was employed to monitor the process. Recrystallization and column chromatography were used to isolate the chemicals.

#### 4.2.1. (*E*)-1-(Furan-2-yl)-3-(3,5,6-trimethylpyrazin-2-yl)prop-2-en-1-one (8a)

Yield: 75%; Mp: 182–183 °C; ^1^H NMR (500 MHz, DMSO-d_6_) *δ* 8.03 (t, *J* = 1.6 Hz, 1H, CH-furan), 7.79 (d, *J* = 17.6 Hz, 1H, CH-furan), 7.66 (dd, *J* = 5.1, 1.6 Hz, 1H), 7.40 (dd, *J* = 5.0, 1.7 Hz, 1H, CH-furan), 7.32 (d, *J* = 17.8 Hz, 1H), 2.52 (s, 3H, CH_3_), 2.41 (s, 6H, 2CH_3_). ^13^C NMR (125 MHz) *δ* 21.0, 21.7, 21.7, 113.6, 116.1, 132.4, 133.2, 145.2, 146.8, 147.0, 148.8, 148.9, 153.0, 181.5; HRMS (ESI) *m*/*z*: 242.2785, microanalysis calculated for C_14_H_14_N_2_O_2_ (242.2780), C, 69.41; H, 5.82; N, 11.56. Found C: 69.49%, H: 5.88%, N: 11.55%.

#### 4.2.2. (*E*)-1-(5-Methylfuran-2-yl)-3-(3,5,6-trimethylpyrazin-2-yl)prop-2-en-1-one (8b)

Yield: 61%; Mp: 189–190 °C; ^1^H NMR (500 MHz, DMSO-d_6_ 7.79 (d, *J* = 17.8 Hz, 1H), 7.49 (d, *J* = 17.8 Hz, 1H), 7.25 (d, *J* = 5.1 Hz, 1H, CH-furan), 6.45 (dd, *J* = 5.1, 0.7 Hz, 1H, CH-furan), 2.42–2.37 (m, 12H, 4CH_3_). ^13^C NMR (125 MHz) *δ* 14.1, 21.0, 21.7, 21.7, 109.7, 119.0, 132.0, 133.3, 145.2, 147.0, 148.8, 148.9, 151.8, 158.2, 181.7.; HRMS (ESI) *m*/*z*: 256.3055, microanalysis calculated for C_14_H_14_N_2_O_2_ (256.3050), C, 70.29; H, 6.29; N, 10.93. Found C: 70.32%, H: 6.30%, N: 10.90%.

#### 4.2.3. (*E*)-1-(Benzofuran-2-yl)-3-(3,5,6-trimethylpyrazin-2-yl)prop-2-en-1-one (8c)

Yield: 42%; Mp: 210–211 °C; ^1^H NMR (500 MHz, DMSO-d_6_) *δ* 8.83 (dd, *J* = 7.0, 2.1 Hz, 1H), 7.79 (d, *J* = 17.8 Hz, 1H), 7.75–7.65 (m, 2H), 7.58 (d, *J* = 17.8 Hz, 1H), 7.46–7.36 (m, 2H), 2.52 (s, 3H, CH_3_), 2.41 (s, 6H, 2CH_3_). ^13^C NMR (125 MHz) *δ* 21.0, 21.7, 21.7, 112.2, 112.6, 123.3, 123.7, 125.3, 129.0, 131.7, 133.3, 145.2, 147.0, 148.8, 148.9, 154.3, 154.3, 181.6. HRMS (ESI) *m*/*z*: 292.3384, microanalysis calculated for C_18_H_16_N_2_O_2_ (292.338), C, 73.95; H, 5.52; N, 9.58; found C: 73.99%, H: 5.55%, N: 9.60%.

#### 4.2.4. (*E*)-1-(5-Methoxybenzofuran-2-yl)-3-(3,5,6-trimethylpyrazin-2-yl)prop-2-en-1-one (8d)

Yield: 51%; Mp: 209–210 °C; ^1^H NMR (500 MHz, DMSO-d_6_) *δ* 7.79 (d, *J* = 17.8 Hz, 1H), 7.64 (d, *J* = 2.2 Hz, 1H, CH-furan), 7.61–7.50 (m, 2H), 7.12–7.04 (m, 2H), 3.81 (s, 3H, CH_3_), 2.40 (d, *J* = 2.6 Hz, 9H, 3CH_3_). ^13^C NMR (125 MHz) *δ* 21.0, 21.7, 21.7, 55.3, 104.9, 111.1, 113.3, 114.9, 128.6, 131.7, 133.3, 145.2, 147.0, 148.8, 148.9, 150.8, 154.3, 156.5, 181.5. HRMS (ESI) *m*/*z*: 322.3646, microanalysis calculated for C_19_H_18_N_2_O_3_ (322.364), C, 70.79; H, 5.63; N, 8.69; found C, 70.78; H, 5.65; N, 8.68.

#### 4.2.5. (*E*)-1-(Thiophen-2-yl)-3-(3,5,6-trimethylpyrazin-2-yl)prop-2-en-1-one (8e)

Yield: 51%; Mp: 209–210 °C; ^1^H NMR (500 MHz, DMSO-d_6_) *δ* 7.91 (dd, *J* = 6.4, 1.6 Hz, 1H, CH-thiophene), 7.78 (d, *J* = 15.9 Hz, 1H), 7.64 (dd, *J* = 5.3, 1.6 Hz, 1H, CH-thiophene), 7.30 (d, *J* = 16.1 Hz, 1H), 7.17 (dd, *J* = 6.3, 5.4 Hz, 1H, CH-thiophene), 2.52 (s, 3H, CH_3_), 2.41 (s, 6H, 2CH_3_). ^13^C NMR (125 MHz, acetone-d_6_) *δ* 20.9, 21.6, 21.6, 125.2, 128.6, 131.2, 132.1, 134.0, 144.6, 145.4, 147.3, 148.8, 148.9, 183.1. HRMS (ESI) *m*/*z*: 258.3394, microanalysis calculated for C_14_H_14_N_2_OS (258.339), C, 65.09; H, 5.46; N, 10.84; S, 12.41; found C, 65.07; H, 5.45; N, 10.82; S, 12.45.

#### 4.2.6. (*E*)-1-(5-Methylthiophen-2-yl)-3-(3,5,6-trimethylpyrazin-2-yl)prop-2-en-1-one (8f)

Yield: 62%; Mp: 227–228 °C; ^1^H NMR (500 MHz, DMSO-d_6_) *δ* 7.78 (d, *J* = 15.9 Hz, 1H), 7.63 (d, *J* = 6.4 Hz, 1H, CH-thiophene), 7.48 (d, *J* = 15.9 Hz, 1H), 6.94–6.89 (m, 1H, CH-thiophene), 2.50 (d, *J* = 0.6 Hz, 6H, 2CH_3_), 2.40 (d, *J* = 2.6 Hz, 6H, 2CH_3_). ^13^C NMR (125 MHz, acetone-d_6_) *δ* 15.9, 20.9, 21.6, 21.6, 125.4, 127.3, 131.7, 133.7, 141.6, 145.4, 145.7, 147.3, 148.8, 148.9, 182.8. HRMS (ESI) *m*/*z*: 272.3662, microanalysis calculated for C_15_H_16_N_2_OS (272.366), C, 66.15; H, 5.92; N, 10.29; S, 11.77; found C, 66.12; H, 5.94; N, 10.27; S, 11.79.

#### 4.2.7. (*E*)-1-(Benzo[*b*]thiophen-2-yl)-3-(3,5,6-trimethylpyrazin-2-yl)prop-2-en-1-one (8g)

Yield: 60%; Mp: 217–218 °C; chemical formula: C_18_H_16_N_2_OS, molecular weight: 308.399, HRMS (ESI) *m*/*z*: 308.3996; elemental analysis calculated: C, 70.10; H, 5.23; N, 9.08; S, 10.40; found C, 70.12; H, 5.25; N, 9.05; S, 10.44; ^1^H NMR (500 MHz, DMSO-d_6_) *δ* 8.08–8.02 (m, 1H), 7.99 (d, *J* = 2.2 Hz, 1H), 7.88–7.83 (m, 1H), 7.78 (d, *J* = 15.9 Hz, 1H), 7.57 (d, *J* = 15.9 Hz, 1H), 7.47–7.41 (m, 1H), 7.36 (td, *J* = 7.3, 1.1 Hz, 1H), 2.52 (s, 3H, CH_3_), 2.41 (s, 6H, 2CH_3_). ^13^C NMR (125 MHz, acetone-d_6_) *δ* 20.9, 21.6, 21.6, 123.1, 124.9, 125.1, 125.4, 126.2, 127.0, 133.3, 139.8, 140.2, 141.9, 145.4, 147.3, 148.8, 148.9, 183.6.

#### 4.2.8. (*E*)-1-(4-(Furan-2-yl)phenyl)-3-(3,5,6-trimethylpyrazin-2-yl)prop-2-en-1-one (9a)

Yield: 47%; Mp: 171–172 °C; chemical formula: C_20_H_18_N_2_O_2_; molecular weight: 318.376; elemental analysis: C, 75.45; H, 5.70; N, 8.80; found: C, 75.46; H, 5.72; N, 8.77; ^1^H NMR (500 MHz, DMSO-d_6_) *δ* 8.00–7.90 (m, 5H), 7.74 (t, *J* = 1.4 Hz, 1H, CH-furan), 7.62 (d, *J* = 16.3 Hz, 1H), 6.89 (dd, *J* = 4.9, 1.7 Hz, 1H, CH-furan), 6.64 (dd, *J* = 4.9, 1.2 Hz, 1H, CH-furan), 2.52 (s, 3H, CH_3_), 2.40 (s, 6H, 2CH_3_). ^13^C NMR (125 MHz, acetone-d_6_) *δ* 20.9, 21.6, 21.6, 107.6, 111.0, 124.5, 126.7, 129.2, 132.9, 134.0, 138.2, 142.3, 145.2, 147.3, 148.8, 148.9, 153.0, 189.5.

#### 4.2.9. (*E*)-1-(4-(5-Methylfuran-2-yl)phenyl)-3-(3,5,6-trimethylpyrazin-2-yl)prop-2-en-1-one (9b)

Yield: 49%; Mp: 174–175 °C; chemical formula: C_21_H_20_N_2_O_2_; molecular weight: 332.403; elemental analysis: C, 75.88; H, 6.06; N, 8.43; found: C, 75.85; H, 6.05; N, 8.41; ^1^H NMR (500 MHz, DMSO-d_6_) *δ* 8.01–7.89 (m, 3H), 7.62 (d, *J* = 16.2 Hz, 1H), 6.83 (d, *J* = 5.3 Hz, 1H, CH-furan), 6.32 (d, *J* = 5.3 Hz, 1H, CH-furan), 2.40 (d, *J* = 2.4 Hz, 9H, 3CH_3_), 2.35 (s, 3H, CH_3_). ^13^C NMR (125 MHz, acetone-d_6_) *δ* 13.8, 20.9, 21.6, 21.6, 106.5, 107.1, 124.8, 126.7, 129.2, 132.9, 134.0, 138.2, 145.2, 147.3, 148.8, 148.9, 151.9, 152.6, 189.5.

#### 4.2.10. (*E*)-1-(4-(Benzofuran-2-yl)phenyl)-3-(3,5,6-trimethylpyrazin-2-yl)prop-2-en-1-one (9c)

Yield: 41%; Mp: 181–182 °C; chemical formula: C_24_H_20_N_2_O_2_; molecular weight: 368.436; elemental analysis: C, 78.24; H, 5.47; N, 7.60; Found: C, 78.26; H, 5.49; N, 7.61; ^1^H NMR (500 MHz, DMSO-d_6_) *δ* 8.02–7.90 (m, 5H), 7.62 (d, *J* = 16.3 Hz, 1H), 7.55–7.48 (m, 1H), 7.48–7.41 (m, 1H), 7.33 (d, *J* = 9.0, 1.5 Hz, 2H), 7.19 (d, *J* = 2.2 Hz, 1H), 2.52 (s, 3H, CH_3_), 2.41 (s, 6H, 2CH_3_). ^13^C NMR (125 MHz, acetone-d_6_) *δ* 20.9, 21.6, 21.6, 102.0, 111.6, 121.5, 123.6, 124.9, 125.3, 126.7, 128.7, 129.1, 132.9, 134.3, 138.3, 145.2, 147.3, 148.8, 148.9, 155.5, 156.4, 189.5.

#### 4.2.11. (*E*)-1-(4-(Thiophen-2-yl)phenyl)-3-(3,5,6-trimethylpyrazin-2-yl)prop-2-en-1-one (9d)

Yield: 50%; Mp: 180–181 °C; chemical formula: C_20_H_18_N_2_OS; molecular weight: 334.437; elemental analysis: C, 71.83; H, 5.43; N, 8.38; S, 9.59; found: C, 71.85; H, 5.41; N, 8.35; S, 9.62; ^1^H NMR (500 MHz, DMSO-d_6_) *δ* 8.02 (d, *J* = 7.8 Hz, 3H), 7.96–7.90 (m, 4H), 7.69–7.58 (m, 2H, 2CH-thiophene), 2.52 (s, 3H, CH_3_), 2.40 (s, 6H, 2CH_3_). ^13^C NMR (125 MHz, acetone-d_6_) *δ* 20.9, 21.6, 21.6, 124.6, 125.9, 126.7, 126.7, 127.7, 129.0, 132.9, 138.1, 138.6, 143.0, 145.2, 147.3, 148.8, 148.9, 189.5.

#### 4.2.12. (*E*)-1-(4-(4-Methylthiophen-2-yl)phenyl)-3-(3,5,6-trimethylpyrazin-2-yl)prop-2-en-1-one (9e)

Yield: 47%; Mp: 175–176 °C; chemical formula: C_21_H_20_N_2_OS; molecular weight: 348.464; elemental analysis: C, 72.38; H, 5.79; N, 8.04; S, 9.20; found: C, 72.39; H, 5.82; N, 8.05; S, 9.22; ^1^H NMR (500 MHz, DMSO-d_6_) *δ* 8.02 (d, *J* = 7.8 Hz, 2H), 7.97–7.90 (m, 3H), 7.62 (d, *J* = 16.3 Hz, 1H), 7.09–7.05 (m, 1H, CH-thiophene), 6.85 (s, 1H, CH-thiophene), 2.40 (d, *J* = 2.6 Hz, 9H, 3CH_3_), 2.29 (s, 3H, CH_3_). ^13^C NMR (125 MHz, acetone-d_6_) *δ* 15.5, 20.9, 21.6, 21.6, 125.8, 126.5, 126.7, 126.7, 129.0, 132.9, 137.7, 138.2, 138.2, 141.6, 145.2, 147.3, 148.8, 148.9, 189.5.

#### 4.2.13. (*E*)-1-(4-(3-Chlorothiophen-2-yl)phenyl)-3-(3,5,6-trimethylpyrazin-2-yl)prop-2-en-1-one (9f)

Yield: 41%; Mp: 164–165 °C; chemical formula: C_20_H_17_ClN_2_OS; molecular weight: 368.879; elemental analysis: C, 65.12; H, 4.65; N, 7.59; S, 8.69; found: C, 65.15; H, 4.65; N, 7.61; S, 8.72; ^1^H NMR (500 MHz, DMSO-d_6_) *δ* 8.07 (d, *J* = 7.7 Hz, 2H), 7.93 (d, *J* = 16.2 Hz, 1H), 7.86 (d, *J* = 7.7 Hz, 2H), 7.62 (d, *J* = 16.2 Hz, 1H), 7.46 (d, *J* = 4.3 Hz, 1H, CH-thiophene), 7.16 (d, *J* = 4.3 Hz, 1H, CH-thiophene), 2.52–2.40 (m, 9H, 3CH_3_). ^13^C NMR (125 MHz, acetone-d_6_) *δ* 20.9, 21.6, 21.6, 126.7, 127.2, 129.2, 129.5, 131.0, 131.7, 132.9, 135.4, 137.8, 138.6, 145.2, 147.3, 148.8, 148.9, 189.5.

#### 4.2.14. (*E*)-1-(4-(5-Bromothiophen-2-yl)phenyl)-3-(3,5,6-trimethylpyrazin-2-yl)prop-2-en-1-one (9g)

Yield: 48%; Mp: 179–180 °C; chemical formula: C_20_H_17_BrN_2_OS; molecular weight: 413.333; elemental analysis: C, 58.12; H, 4.15; N, 6.78; S, 7.76; found: C, 58.14; H, 4.15; N, 6.75; S, 7.77; ^1^H NMR (500 MHz, DMSO-d_6_) *δ* 8.02 (d, *J* = 7.8 Hz, 4H), 7.98–7.90 (m, 1H), 7.62 (d, *J* = 16.2 Hz, 1H), 7.19 (d, *J* = 6.9 Hz, 1H, CH-thiophene), 7.12 (d, *J* = 6.9 Hz, 1H, CH-thiophene), 2.40 (d, *J* = 2.4 Hz, 9H, 3CH_3_). ^13^C NMR (125 MHz, acetone-d_6_) *δ* 20.97, 21.6, 21.6, 112.7, 124.1, 126.0, 126.7, 128.9, 129.0, 132.9, 137.9, 138.1, 142.6, 145.2, 147.3, 148.8, 148.9, 189.5.

### 4.3. Synthesis of ligustrazine based chalcone oxime analogs

To synthesize oxime analogs, the most effective ligustrazine-containing chalcones were selected as precursors. Hydroxylamine hydrochloride (2 mmol) was reacted with each chosen chemical (1 mmol) in 10 mL ethanol to produce the corresponding oximes in the reaction (10a–g and 11a–g). TLC found that the typical reaction took 6–8 hours to complete. For the purification of certain items, column chromatography with the 70/30 mixture of hexane and water was utilized.

#### 4.3.1. (1*E*,2*E*)-1-(Furan-2-yl)-3-(3,5,6-trimethylpyrazin-2-yl)prop-2-en-1-one oxime (10a)

Yield: 51%; Mp: 181–182 °C; chemical formula: C_14_H_15_N_3_O_2_; molecular weight: 257.293; elemental analysis: C, 65.36; H, 5.88; N, 16.33; found: C, 65.39; H, 5.89; N, 16.32; ^1^H NMR (500 MHz, DMSO-d_6_) *δ* 11.53 (s, 1H, OH), 7.91 (d, *J* = 17.3 Hz, 1H), 7.80–7.76 (m, 1H, CH-furan), 7.41 (d, *J* = 17.3 Hz, 1H), 6.74 (dd, *J* = 4.5, 1.5 Hz, 1H, CH-furan), 6.68 (dd, *J* = 4.5, 1.5 Hz, 1H, CH-furan), 2.52 (s, 3H), 2.41 (s, 6H). ^13^C NMR (125 MHz, acetone-d_6_) *δ* 20.9, 21.6, 21.6, 111.7, 112.7, 124.4, 127.1, 143.3, 145.6, 147.1, 147.2, 148.8, 148.9, 149.0.

#### 4.3.2. (1*Z*,2*E*)-1-(5-Methylfuran-2-yl)-3-(3,5,6-trimethylpyrazin-2-yl)prop-2-en-1-one oxime (10b)

Yield: 50%; Mp: 194–195 °C; chemical formula: C_15_H_17_N_3_O_2_; molecular weight: 271.320; elemental analysis: C, 66.40; H, 6.32; N, 15.49; found: C, 66.42; H, 6.35; N, 15.47; ^1^H NMR (500 MHz, DMSO-d_6_) *δ* 11.33 (s, 1H, OH), 7.91 (d, *J* = 17.3 Hz, 1H), 7.43 (d, *J* = 17.3 Hz, 1H), 6.47 (d, *J* = 4.7 Hz, 1H, CH-furan), 6.27 (d, *J* = 4.7 Hz, 1H, CH-furan), 2.51 (s, 9H, 3CH_3_), 2.38 (s, 3H, CH_3_). ^13^C NMR (125 MHz, acetone-d_6_) *δ* 14.0, 20.9, 21.6, 21.6, 110.5, 111.1, 126.1, 126.8, 143.8, 147.1, 147.2, 148.8, 148.9, 149.0, 163.5.

#### 4.3.3. (1*Z*,2*E*)-1-(Benzofuran-2-yl)-3-(3,5,6-trimethylpyrazin-2-yl)prop-2-en-1-one oxime (10c)

Yield: 57%; Mp: 191–192 °C; chemical formula: C_18_H_17_N_3_O_2_; molecular weight: 307.353; elemental analysis: C, 70.34; H, 5.58; N, 13.67; found: C, 70.35; H, 5.62; N, 13.65; ^1^H NMR (500 MHz, DMSO-d_6_) *δ* 11.36 (s, 1H), 8.83 (dd, *J* = 7.2, 1.8 Hz, 1H), 7.91 (d, *J* = 17.3 Hz, 1H), 7.69–7.63 (m, 2H), 7.53 (d, *J* = 17.3 Hz, 1H), 7.46–7.36 (m, 1H), 7.27 (d, *J* = 1.9 Hz, 1H), 2.52 (s, 3H, CH_3_), 2.41 (s, 6H, 2CH_3_). ^13^C NMR (125 MHz, acetone-d_6_) *δ* 20.9, 21.6, 21.6, 106.9, 111.4, 121.8, 123.6, 125.2, 126.3, 126.4, 129.1, 145.6, 147.1, 147.2, 148.8, 149.0, 150.5, 154.1.

#### 4.3.4. (1*Z*,2*E*)-1-(5-Methoxybenzofuran-2-yl)-3-(3,5,6-trimethylpyrazin-2-yl)prop-2-en-1-one oxime (10d)

Yield: 55%; Mp: 194–195 °C; chemical formula: C_19_H_19_N_3_O_3_; molecular weight: 337.379; elemental analysis: C, 67.64; H, 5.68; N, 12.46; found: C, 67.65; H, 5.70; N, 12.45; ^1^H NMR (500 MHz, DMSO-d_6_) *δ* 11.36 (s, 1H), 7.91 (d, *J* = 17.3 Hz, 1H), 7.57–7.50 (m, 2H), 7.09 (td, *J* = 4.3, 2.5 Hz, 2H), 7.05 (t, *J* = 2.0 Hz, 1H), 3.81 (s, 3H, CH_3_), 2.40–2.52 (m, 9H, 3CH_3_). ^13^C NMR (125 MHz, acetone-d_6_) *δ* 20.9, 21.6, 21.6, 55.3, 103.9, 105.6, 111.9, 114.8, 126.3, 126.4, 128.4, 145.6, 147.1, 147.2, 148.8, 149.0, 149.9, 150.7, 156.4.

#### 4.3.5. (1*Z*,2*E*)-1-(Thiophen-2-yl)-3-(3,5,6-trimethylpyrazin-2-yl)prop-2-en-1-one oxime (10e)

Yield: 54%; Mp: 153–154 °C; chemical formula: C_14_H_15_N_3_OS; molecular weight: 273.354; elemental analysis: C, 61.52; H, 5.53; N, 15.37; S, 11.73; found: C, 61.55; H, 5.55; N, 15.35; S, 11.75; ^1^H NMR (500 MHz, DMSO-d_6_) *δ* 9.93 (s, 1H), 7.89 (d, *J* = 15.6 Hz, 1H), 7.60 (dd, *J* = 5.3, 1.5 Hz, 1H, CH-thiophene), 7.49 (d, *J* = 15.6 Hz, 1H, CH-thiophene), 7.38 (dd, *J* = 5.8, 1.6 Hz, 1H), 7.23 (t, *J* = 5.6 Hz, 1H, CH-thiophene), 2.52 (s, 3H, CH_3_), 2.41 (s, 6H, 2CH_3_). ^13^C NMR (125 MHz, acetone-d_6_) *δ* 20.9, 21.6, 21.6, 123.6, 126.1, 127.6, 128.3, 130.1, 135.7, 146.9, 147.3, 147.4, 148.8, 149.0.

#### 4.3.6. (1*Z*,2*E*)-1-(5-Methylthiophen-2-yl)-3-(3,5,6-trimethylpyrazin-2-yl)prop-2-en-1-one oxime (10f)

Yield: 57%; Mp: 199–200 °C; chemical formula: C_15_H_17_N_3_OS; molecular weight: 287.381; elemental analysis: C, 62.69; H, 5.96; N, 14.62; S, 11.16; found: C, 62.70; H, 5.98; N, 14.60; S, 11.14; ^1^H NMR (500 MHz, DMSO-d_6_) *δ* 9.94 (s, 1H), 7.89 (d, *J* = 15.6 Hz, 1H), 7.55 (d, *J* = 15.6 Hz, 1H), 7.08 (d, *J* = 6.0 Hz, 1H, CH-thiophene), 6.82 (d, *J* = 6.0 Hz, 1H, CH-thiophene), 2.50 (s, 3H, CH_3_), 2.40 (d, *J* = 2.4 Hz, 9H, 3CH_3_). ^13^C NMR (125 MHz, acetone-d_6_) *δ* 15.9, 20.9, 21.6, 21.6, 123.7, 125.7, 127.3, 127.6, 133.9, 142.9, 146.0, 146.9, 147.3, 148.8, 149.0.

#### 4.3.7. (1*Z*,2*E*)-1-(Benzo[*b*]thiophen-2-yl)-3-(3,5,6-trimethylpyrazin-2-yl)prop-2-en-1-one oxime (10g)

Yield: 52%; Mp: 180–181 °C; chemical formula: C_18_H_17_N_3_OS; molecular weight: 323.414; elemental analysis: C, 66.85; H, 5.30; N, 12.99; S, 9.91; found: C, 66.87; H, 5.32; N, 12.96; S, 9.92; ^1^H NMR (500 MHz, DMSO-d_6_) *δ* 9.96 (s, 1H), 8.05 (d, *J* = 7.3 Hz, 1H), 7.92–7.83 (m, 2H), 7.61 (d, *J* = 15.6 Hz, 1H), 7.52 (d, *J* = 1.9 Hz, 1H), 7.47–7.41 (m, 1H), 7.39–7.33 (m, 1H), 2.52 (s, 3H, CH_3_), 2.41 (s, 6H, 2CH_3_). ^13^C NMR (125 MHz, acetone-d_6_) *δ* 20.9, 21.6, 21.6, 122.3, 123.8, 123.8, 124.1, 125.1, 126.1, 127.0, 136.4, 139.8, 141.1, 146.9, 147.3, 148.3, 148.8, 149.0.

#### 4.3.8. (1*Z*,2*E*)-1-(4-(Furan-2-yl)phenyl)-3-(3,5,6-trimethylpyrazin-2-yl)prop-2-en-1-one oxime (11a)

Yield: 29%; Mp: 194–195 °C; chemical formula: C_20_H_19_N_3_O_2_; molecular weight: 333.391; elemental analysis: C, 72.05; H, 5.74; N, 12.60; found: C, 72.07; H, 5.78; N, 12.61; ^1^H NMR (500 MHz, DMSO-d_6_) *δ* 9.51 (s, 1H), 7.89 (d, *J* = 7.6 Hz, 1H), 7.81 (d, *J* = 15.8 Hz, 1H), 7.74 (s, 1H), 7.56 (d, *J* = 7.6 Hz, 2H), 7.44 (d, *J* = 15.8 Hz, 2H), 6.89 (dd, *J* = 4.7, 1.5 Hz, 1H, CH-furan), 6.67–6.62 (m, 1H, CH-furan), 2.40 (d, *J* = 2.4 Hz, 9H, 3CH_3_). ^13^C NMR (125 MHz, acetone-d_6_) *δ* 20.9, 21.6, 21.6, 107.6, 111.0, 124.4, 124.8, 126.9, 127.5, 132.1, 133.0, 142.3, 147.0, 147.3, 148.8, 149.0, 153.1, 154.1.

#### 4.3.9. (1*Z*,2*E*)-1-(4-(5-Methylfuran-2-yl)phenyl)-3-(3,5,6-trimethylpyrazin-2-yl)prop-2-en-1-one oxime (11b)

Yield: 26%; Mp: 214–215 °C; chemical formula: C_21_H_21_N_3_O_2_; molecular weight: 347.418; elemental analysis: C, 72.60; H, 6.09; N, 12.10; found: C, 72.62; H, 6.12; N, 12.14; ^1^H NMR (500 MHz, DMSO-d_6_) *δ* 9.51 (s, 1H), 7.89 (d, *J* = 7.6 Hz, 2H), 7.81 (d, *J* = 15.8 Hz, 1H), 7.56 (d, *J* = 7.6 Hz, 2H), 7.44 (d, *J* = 15.8 Hz, 1H), 6.83 (d, *J* = 5.3 Hz, 1H, CH-furan), 6.32 (d, *J* = 5.3 Hz, 1H, CH-furan), 2.40 (d, *J* = 2.4 Hz, 9H, 3CH_3_), 2.35 (s, 3H, CH_3_). ^13^C NMR (125 MHz, acetone-d_6_) *δ* 13.8, 20.9, 21.6, 21.6, 106.5, 107.1, 124.4, 125.3, 126.9, 127.5, 132.2, 133.0, 147.0, 147.3, 148.8, 149.0, 151.9, 152.6, 154.1.

#### 4.3.10. (1*Z*,2*E*)-1-(4-(Benzofuran-2-yl)phenyl)-3-(3,5,6-trimethylpyrazin-2-yl)prop-2-en-1-one oxime (11c)

Yield: 19%; Mp: 224–225 °C; chemical formula: C_24_H_21_N_3_O_2_; molecular weight: 383.451; elemental analysis: C, 75.18; H, 5.52; N, 10.96; found: C, 75.19; H, 5.55; N, 10.95; ^1^H NMR (500 MHz, DMSO-d_6_) *δ* 9.51 (s, 1H), 7.89 (d, *J* = 7.6 Hz, 2H), 7.81 (d, *J* = 15.8 Hz, 1H), 7.58 (d, *J* = 7.6 Hz, 2H), 7.55–7.50 (m, 1H), 7.48–7.41 (m, 2H), 7.33 (pd, *J* = 8.7, 1.2 Hz, 2H), 7.19 (d, *J* = 1.9 Hz, 1H), 2.40 (d, *J* = 2.4 Hz, 9H, 3CH_3_). ^13^C NMR (125 MHz, acetone-d_6_) *δ* 20.9, 21.6, 21.6, 102.0, 111.6, 121.5, 123.6, 124.4, 124.9, 125.7, 126.9, 127.5, 128.7, 132.0, 133.1, 147.0, 147.3, 148.8, 149.0, 154.1, 155.5, 156.4.

#### 4.3.11. (1*Z*,2*E*)-1-(4-(Thiophen-2-yl)phenyl)-3-(3,5,6-trimethylpyrazin-2-yl)prop-2-en-1-one oxime (11d)

Yield: 32%; Mp: 222–223 °C; chemical formula: C_20_H_19_N_3_OS; molecular weight: 349.452; elemental analysis: C, 68.74; H, 5.48; N, 12.02; S, 9.17; found: C, 68.77; H, 5.49; N, 12.00; S, 9.19; ^1^H NMR (500 MHz, DMSO-d_6_) *δ* 9.51 (s, 1H), 7.95 (d, *J* = 7.2 Hz, 2H), 7.81 (d, *J* = 15.8 Hz, 1H), 7.69–7.61 (m, 4H), 7.44 (d, *J* = 15.8 Hz, 1H), 7.23–7.18 (m, 1H, CH-thiophene), 2.40 (d, *J* = 2.4 Hz, 9H, 3CH_3_). ^13^C NMR (125 MHz, acetone-d_6_) *δ* 20.9, 21.6, 21.6, 124.4, 124.6, 126.0, 126.7, 127.5, 127.7, 132.6, 136.5, 143.0, 147.0, 147.3, 148.8, 149.0, 154.2.

#### 4.3.12. (1*Z*,2*E*)-1-(4-(4-Methylthiophen-2-yl)phenyl)-3-(3,5,6-trimethylpyrazin-2-yl)prop-2-en-1-one oxime (11e)

Yield: 29%; Mp: 194–195 °C; chemical formula: C_21_H_21_N_3_OS; molecular weight: 363.479; elemental analysis: C, 69.39; H, 5.82; N, 11.56; S, 8.82; found: C, 69.41; H, 5.80; N, 11.55; S, 8.80; ^1^H NMR (500 MHz, DMSO-d_6_) *δ* 9.51 (s, 1H), 7.96 (d, *J* = 7.2 Hz, 2H), 7.81 (d, *J* = 15.8 Hz, 1H), 7.63 (d, *J* = 7.2 Hz, 2H), 7.44 (d, *J* = 15.8 Hz, 1H), 7.09–7.05 (m, 1H), 6.85 (s, 1H), 2.40 (d, *J* = 2.4 Hz, 9H, 3CH_3_), 2.29 (s, 3H, CH_3_). ^13^C NMR (125 MHz, acetone-d_6_) *δ* 15.5, 20.9, 21.6, 21.6, 124.4, 125.9, 126.5, 126.6, 126.7, 127.5, 132.6, 136.0, 137.7, 141.5, 147.0, 147.3, 148.8, 149.0, 154.2.

#### 4.3.13. (1*Z*,2*E*)-1-(4-(3-Chlorothiophen-2-yl)phenyl)-3-(3,5,6-trimethylpyrazin-2-yl)prop-2-en-1-one oxime (11f)

Yield: 34%; Mp: 219–220 °C; chemical formula: C_20_H_18_ClN_3_OS; molecular weight: 383.894; elemental analysis: C, 62.57; H, 4.73; N, 10.95; S, 8.35; found: C, 62.55; H, 4.75; N, 10.95; S, 8.35; ^1^H NMR (500 MHz, DMSO-d_6_) *δ* 9.51 (s, 1H), 7.86–7.78 (m, 3H), 7.64 (d, *J* = 7.2 Hz, 2H), 7.49–7.41 (m, 2H), 7.16 (d, *J* = 4.3 Hz, 1H), 2.40 (d, *J* = 2.4 Hz, 9H, 3CH_3_). ^13^C NMR (125 MHz, acetone-d_6_) *δ* 20.9, 21.6, 21.6, 124.4, 126.9, 127.5, 127.6, 129.5, 131.0, 131.7, 132.3, 135.4, 136.9, 147.0, 147.3, 148.9, 149.0, 154.2.

#### 4.3.14. (1*Z*,2*E*)-1-(4-(5-Bromothiophen-2-yl)phenyl)-3-(3,5,6-trimethylpyrazin-2-yl)prop-2-en-1-one oxime (11g)

Yield: 28%; Mp: 204–205 °C; chemical formula: C_20_H_18_BrN_3_OS; molecular weight: 428.348; elemental analysis: C, 56.08; H, 4.24; N, 9.81; S, 7.48; found: C, 56.10; H, 4.25; N, 9.85; S, 7.49; ^1^H NMR (500 MHz, DMSO-d_6_) *δ* 9.51 (s, 1H), 8.00 (d, *J* = 7.2 Hz, 2H), 7.81 (d, *J* = 15.8 Hz, 1H), 7.63 (d, *J* = 7.2 Hz, 2H), 7.44 (d, *J* = 15.8 Hz, 1H), 7.19 (d, *J* = 6.9 Hz, 1H), 7.12 (d, *J* = 6.9 Hz, 1H), 2.40 (d, *J* = 2.4 Hz, 9H, 3CH_3_). ^13^C NMR (125 MHz, acetone-d_6_) *δ* 20.9, 21.6, 21.6, 112.7, 124.1, 124.4, 126.0, 126.6, 127.5, 128.9, 132.5, 136.1, 142.6, 147.0, 147.3, 148.8, 149.0, 154.2.

### 4.4. Cell culture and reagents

The American Type Culture Collection provided the human cancer cell lines used in this study (ATCC, Manassas, VA). In order to sustain the cell lines, they were cultured in DMEM and RPMI-1640 supplemented with 10% FBS (Gibco) and 1% penicillin–streptomycin, respectively (Hyclone). The cells were incubated at 37 °C in an environment containing 5% CO_2_. The DMSO used in this experiment was acquired from Sigma Chemical Co. (St. Louis, MO, USA).

### 4.5. Cell growth inhibition MTT assay

All of the cell lines that were used were grown as described. A new medium was put in place after 24 hours. The new medium had the desired compounds in it at the right concentrations, and there were also control samples (cisplatin and foretinib) with the same amount of the vehicle. In order to make a stock solution of 10 mM, all of the compounds were mixed with DMSO. The final solutions were made by diluting the stock solution with the right volume of liquid in the media, keeping the final DMSO content at 0.5 percent. In every case, a triplicate was always made on the same plate. After 72 hours, 0.25 mg mL^−1^ of MTT was added and incubated for 3 hours. The medium was taken out, and DMSO was added to dissolve the formazan crystals that were in it. To figure out how much the formazan absorbs, an ELISA plate reader was used to measure the absorbance at 570 nm. The background absorbance was measured at 690 nm. This is how the 690 nm background absorbance was taken out of the values at 570 nm. The average of the three repeats for each condition was found. In order to make the data easier to read, it was compared to the DMSO control cells that had 100% survival rate. The curves were made with GraphPad Prism, and the IC_50_ values were found from the plots.

### 4.6. BRAF kinase assay

Commercially available ELISA kits (Invitrogen) were utilized to examine the influence of compounds on BRAF kinase activity in cancer cells. The V600E mutant BRAF kinase assay was used and assay dilution buffer (4 μL) and test compound (1 μL) were incubated with GST-tagged BRAF^V600E^ at 25 °C for 60 minutes. MgCl_2_, ATP, full-length MEK1 (200 ng), and N-terminal histidine-tagged MEK1 (Invitrogen) were diluted with 5 microliters of dilution buffer to initiate the test, which was incubated at 25 °C for 25 minutes. Foretinib was used as positive control. Detailed methods are described in ESI† of this article.

### 4.7. EGFR enzymatic activities assay

Numerous small compounds have been synthesized and tested as EGFR TKIs since the discovery of anti-EGFR medicines for cancer therapy, ranging in generation from first to third generation. The focus of the research is on developing and discovering drugs that specifically target mutant EGFR. It has been found that the epigenetic mutations in the EGFR tyrosine kinase domain can be dealt with by using small-molecule EGFR degraders. To overcome epigenetic alterations and increase the efficacy of EGFR TK inhibitors, researchers have looked into multi-target medicines and combination treatment.^23^ In this study EGFR enzymatic assay was used to evaluate the activities of selected compounds. To achieve a final DMSO concentration of 1% in all reactions, the compounds were diluted in DMSO at a 10% dilution and then added to a 50 mL reaction. Kinase-Glo Plus luminescence kinase test kit was used for the experiment. Foretinib was used as positive control. Detailed instructions can be found in the article's ESI.†

### 4.8. Tubulin polymerization assay

Microtubules play an essential part in cellular functions, making tubulin a good target for antineoplastic drug design because of its indispensable significance. Antimitotic drugs can be anything that interferes with microtubule kinetics. It is possible to use tubulin inhibitors in conjunction with tubulin to limit the protein's stability and so impair the cell's ability to perform its normal functions. As an alternative to typical cytotoxic medications, tubulin inhibitors have been shown to be a very successful technique for eradicating cancerous tissue.^31^ To evaluate the effect of new synthetic compounds, bovine brain tubulin (0.4 mg, 97 percent pure) and a tubulin polymerization kit (Cytoskeleton, Inc. Denver, CO) were used in this study. One hundred microliters of general tubulin buffer were used to mix the experimental compounds with the controls (colchicine, vinorelbine, and paclitaxel). In the article's ESI† details are provided.

### 4.9. *In vitro* c-Met kinase assay

For testing the inhibitory action of test compounds on c-Met recombinant kinases, a homogeneous time-resolved fluorescence (HTRF) assay was employed. Foretinib was used as positive control. Detailed instructions can be found in the article's appendices.

### 4.10. Experimental docking modeling

Molecular docking of 11d, 11g, 8d, and 10d with two enzymes EGFR tyrosine kinase and tubulin was executed utilizing Autodock Vina software^32^ to determine the interactions. The details of EGFR tyrosine kinase (PDB ID: 1M17) and tubulin (PDB ID: 5LYJ) were downloaded from Protein Data Bank [www.rcsb.org/pdb]. Prior to docking simulations, the crystallographic protein files were developed by deleting water molecules, complexed ligands and heteroatoms from both proteins. Charges “q” along with autodock type “t” were added to both pdb files and were saved in pdbqt format by means of Autodock tools.^33^ Meanwhile, ChemBio3D Ultra (version 16.0.0.82) was used to sketch the ligands and optimize their energies. The optimized structures of ligands were changed to pdbqt format by Openbabel software.^34^ The grid box size 64 × 40 × 56 Å positioned at *X* = 23.531, *Y* = 0.879 and *Z* = 56.525 for EGFR tyrosine kinase and a grid box size 40 × 40 × 40 Å positioned at *X* = 16.035, *Y* = 64.991 and *Z* = 40.329 for tubulin protein were defined to make sure the unrestricted movement of ligands and to cover the active sites of both proteins. Other docking parameters for both protein calculations were set as follow; number of binding modes (max) = 50, exhaustiveness value = 32, energy alteration between the worst and best binding modes (max) = 4 kcal mol^−1^ respectively. Discovery Studio Visualizer^35^ was used to analyse the final simulated docking results.

## Funding

This project was supported by Jouf University under research project no. (40/003).

## Data availability

All data generated or analyzed during this study have been included in this article.

## Conflicts of interest

There are no conflicts to declare.

## Supplementary Material

RA-012-D2RA01198K-s001
