# Supplementary material for: Synthesis and evaluation of new chalcones and oximes as anticancer agents
Source: RSC Adv. 2022 Apr 1;12(17):10307–20. doi: 10.1039/d2ra01198k (PMC8973297; doi:10.1039/d2ra01198k)

Electronic Supplementary Material (ESI) for RSC Advances.
This journal is © The Royal Society of Chemistry 2022

Synthesis and evaluation of new chalcones and oximes as anticancer agents

Syed Nasir Abbas Bukhari,[a]+

[a] Syed Nasir Abbas Bukhari
Department of Pharmaceutical Chemistry,
College of Pharmacy, Jouf University, Aljouf,
Sakaka, 2014, Saudi Arabia.
Email: sbukhari@ju.edu.sa
Email: snab_hussaini@yahoo.com
Cell #: 00966565738896

1- MECHANISTIC ASSAY METHODS

1.1.BRAF kinase assay

The BRAF kinase activity of each compound was tested using the V600E mutant BRAF kinase assay. GST-tagged BRAFV600E was pre-incubated for 60 minutes at 25°C with drug (1 µL) and the assay dilution buffer (4 µL). Experimental dilution buffer was diluted with 5 microliters of $MgCl_2$, ATP, recombinant human full-length MEK1 (200 ng), and N-terminal His-tagged MEK1 (Invitrogen) to begin the assay, which was incubated at 25 °C for 25 minutes. The protein denaturation buffer (LDS) solution (5 µL) was used to halt the test. Heat (70° C) was applied for 5 minutes to further denature the protein. Electrophoresis was performed on precast NuPage gel plates (Invitrogen) at a concentration of 4% to 12%. (at 200 V). The precast plates were filled with 10 L of each reaction, and the electrophoresis procedure was allowed to run its course. After electrophoresis, the precast gel plate's front portion (which contained the heated ATP) was cut off and discarded. A phosphor screen was used to generate the dried gel. The negative control was a reaction with no active enzyme, whereas the positive control was a reaction with no inhibitor. Commercially available ELISA kits (Invitrogen) were utilized to examine the influence of substances on cell-based pERK1/2 activity in cancer cells.

1.2. EGFR enzymatic activities assay

For 40 minutes, all of the enzyme reactions were carried out at 30 degrees Celsius. Tris (pH 7.4), $MgCl_2$, 0.1 mg/ml BSA, 1 mM DTT, 10 M ATP and the enzyme substrate (0.2 mg/ml Poly (Glu, Tyr)) are in the 50 L reaction mixture. To achieve a final DMSO concentration of 1% in all reactions, the compounds were diluted in DMSO at a 10% dilution and then added to a 50 mL reaction. Kinase-Glo Plus luminescence kinase test kit was used for the experiment. ATP remaining in solution after the kinase reaction is used to quantify kinase activity. Analyses show a correlation between ATP concentration and luminous signal. Kinase activity appears to have an opposite relationship to this correlation. Nonlinear regression with normalized dose-response fit was used to generate the IC_{50} values using Prism GraphPad software.

1.3. Tubulin polymerization assay

Bovine brain tubulin (0.4 mg, 97 percent pure) with a tubulin polymerization kit (Cytoskeleton, Inc. Denver, CO) were used in this test (80 mM PIPES, 2.0 mM MgCl2, 0.5 mM EGTA, and 1 mM GTP). The tested molecules and the controls (Colchicine, Vinorelbine, and Paclitaxel) (100 µL of general tubulin buffer) were introduced to the system. At 37 °C and 340 nm, the Tecan Microplate Reader was used to measure the pH, temperature, and 340 nm absorbance every 1 minute for 20 minutes. IC_{50} is the concentration of a substance that inhibits polymerization by 50% after 20 minutes of incubation.

1.4. In vitro c-Met kinase assay

The homogeneous time resolved fluorescence (HTRF) assay was used to assess the inhibitory activity of test drugs on c-Met recombinant kinases. Following the manufacturer's instructions, the concentrations of enzyme, ATP, and substrate were tuned using an HTRF KinEASE kit (Cisbio, Codolet, France). Kinase reaction buffer (250 mM HEPES (pH 7.0), ATP, orthovanadate, $MgCl_2$,

0.5 mM DTT, 0.05 percent BSA, 0.1 percent NaN_3) was used to commence the reaction, which involved the addition of ATP to a mixture comprising c-Met enzyme, peptide substrates, and serially diluted inhibitor. A Victor multi-label reader was used to measure the TR-FRET signal after detection reagents were applied. Nonlinear regression was used to fit the curve, and GraphPad Prism was used to compute the IC_{50} (GraphPad, La Jolla, CA, USA).

NMR spectra of selected compounds

Copies of the $^{1}H/^{13}C$ NMR spectra

^{1}H NMR spectrum of **8a**

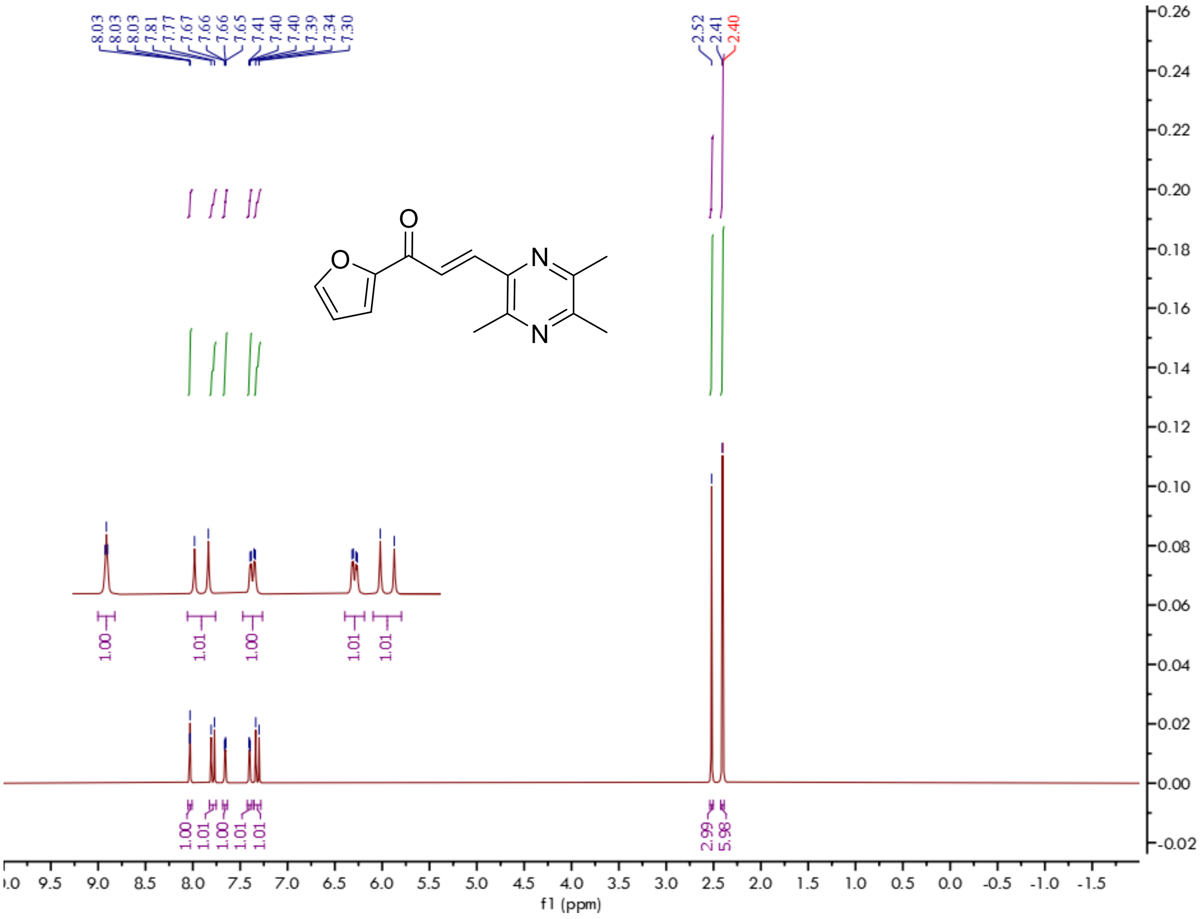

^{13}C NMR spectrum of **8a**

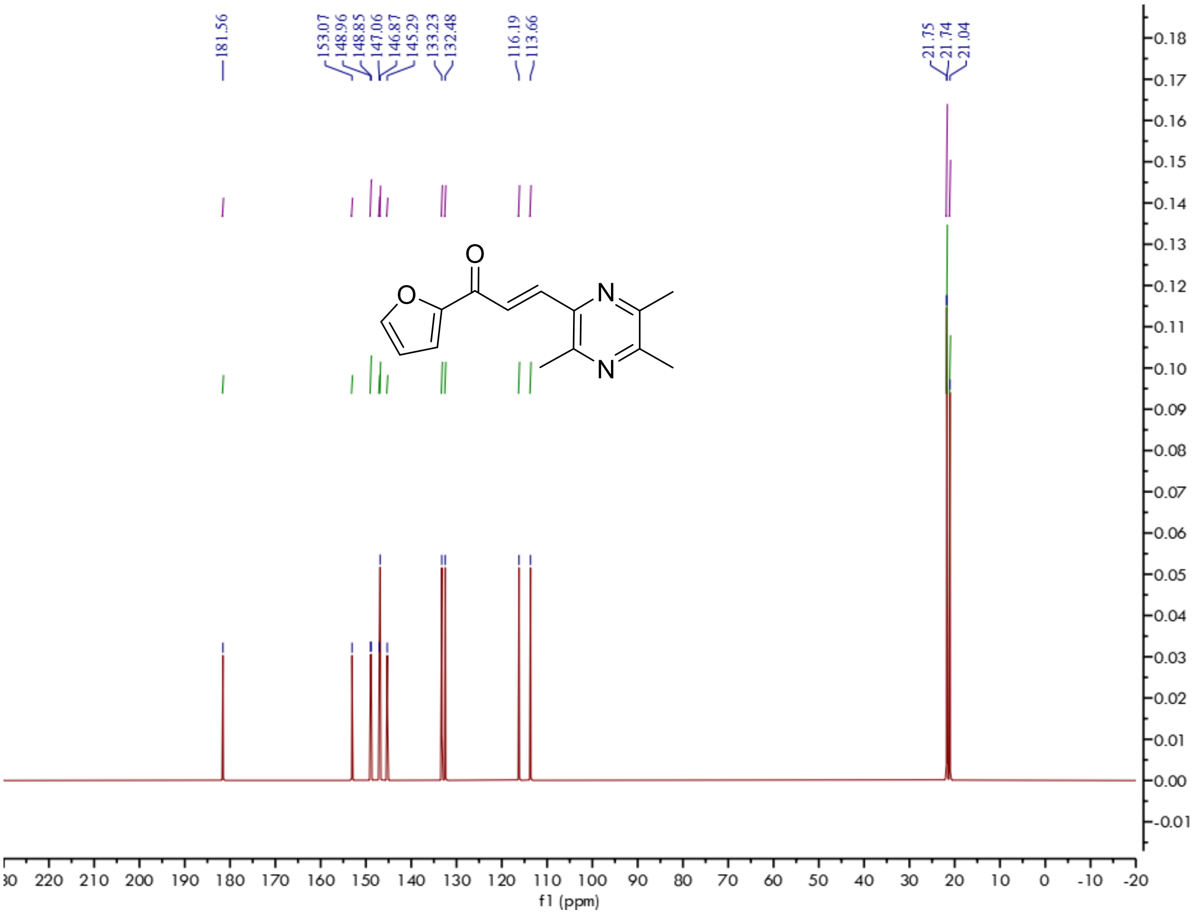

^{1}H NMR spectrum of **8b**

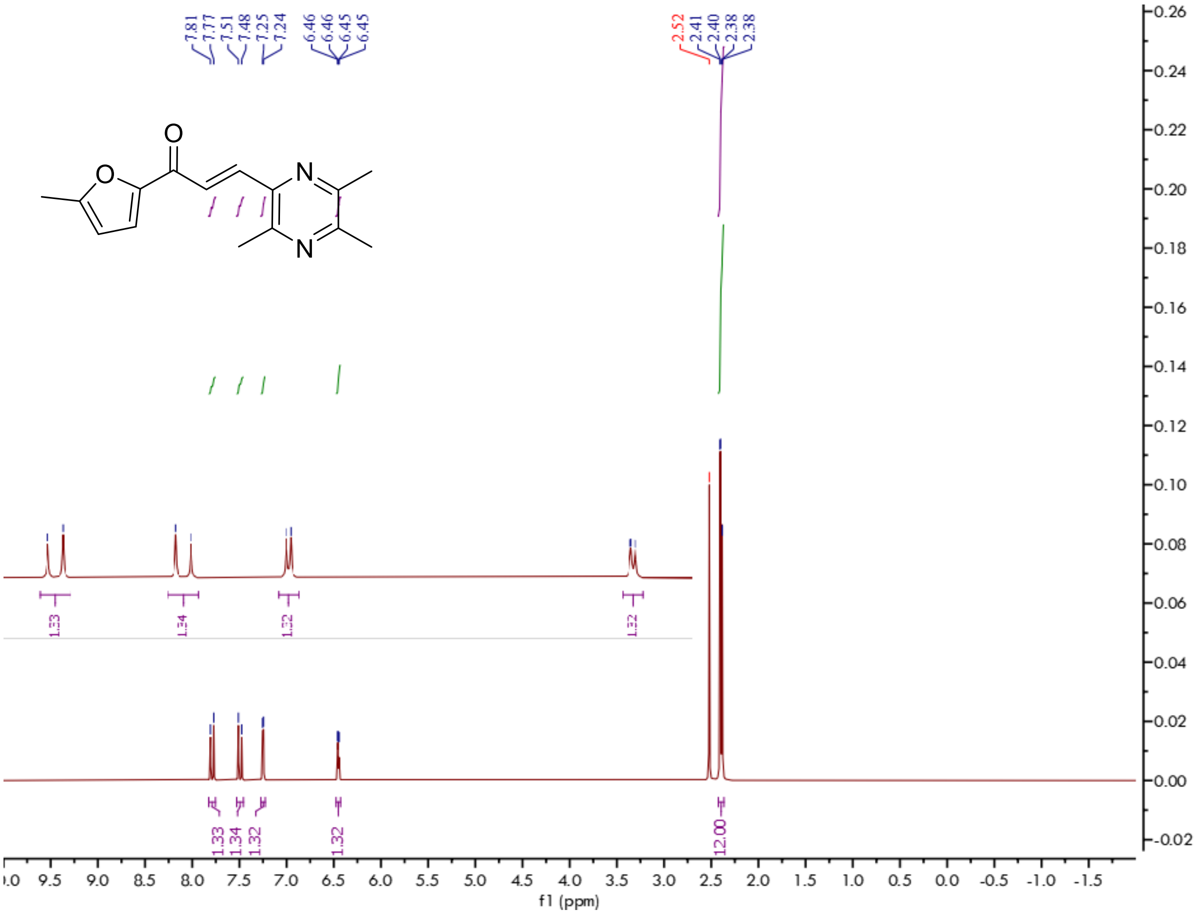

^{13}C NMR spectrum of **8b**

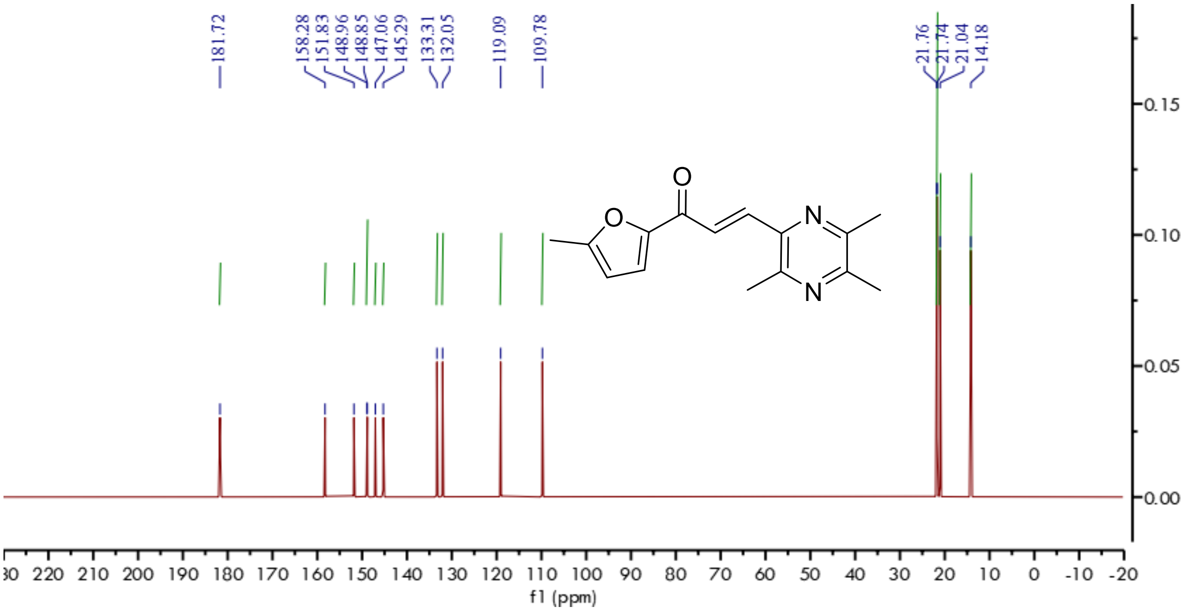

^{1}H NMR spectrum of **8c**

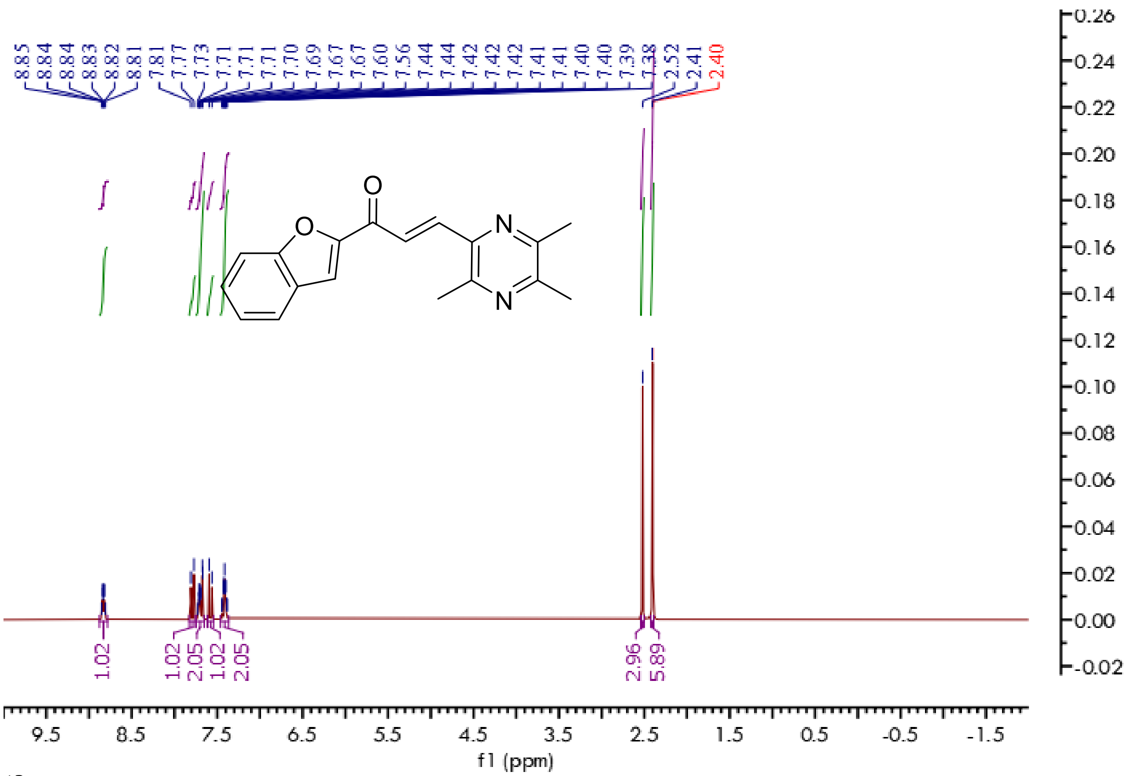

^{13}C NMR spectrum of **8c**

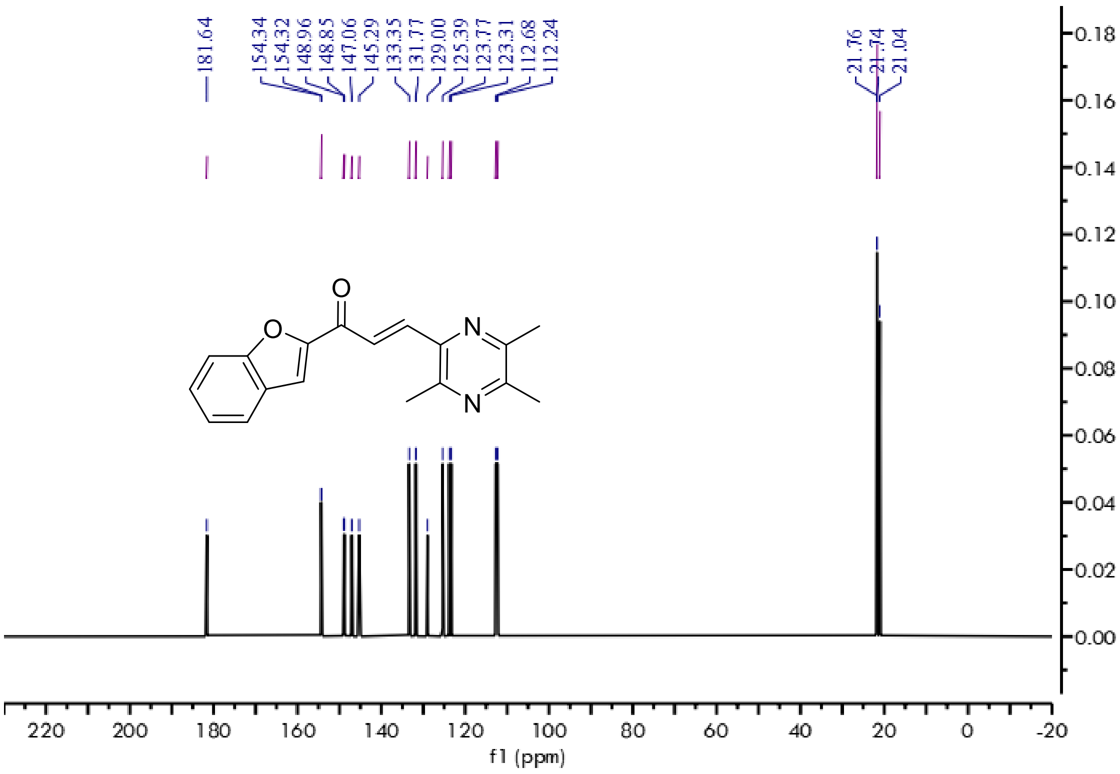

^{1}H NMR spectrum of **8d**

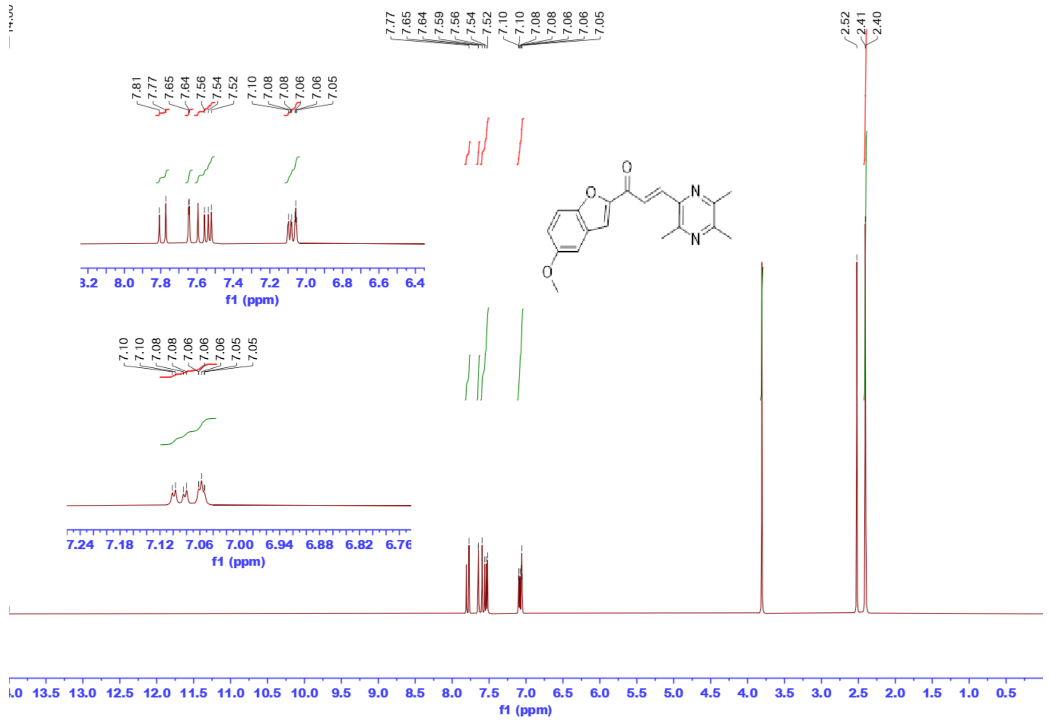

^{13}C NMR spectrum of **8d**

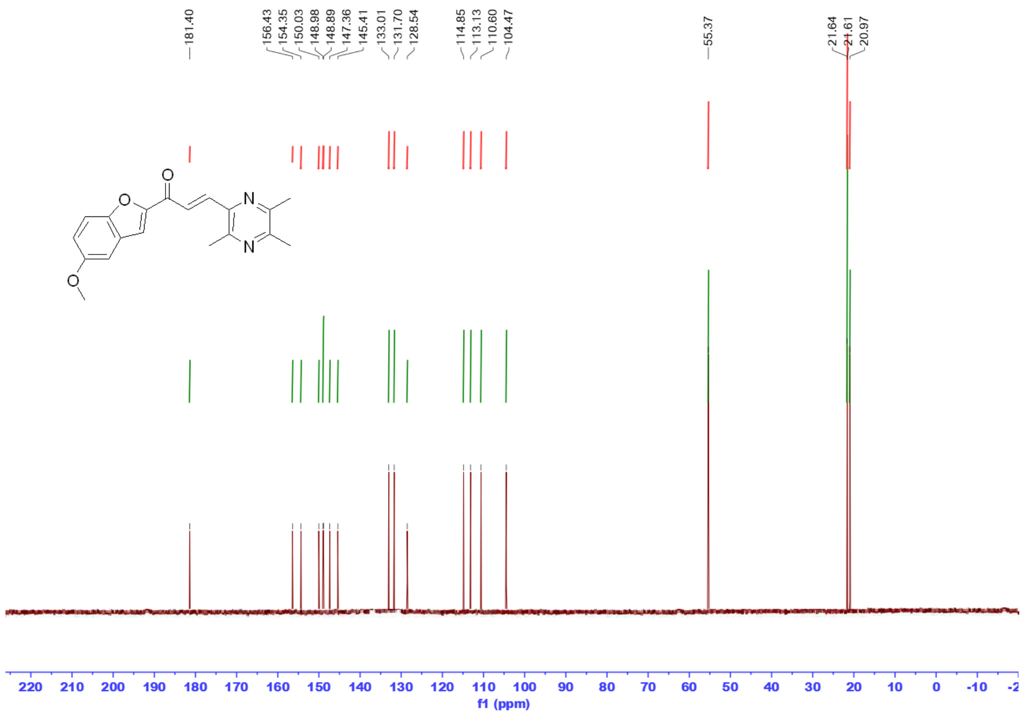

^{1}H NMR spectrum of **8e**

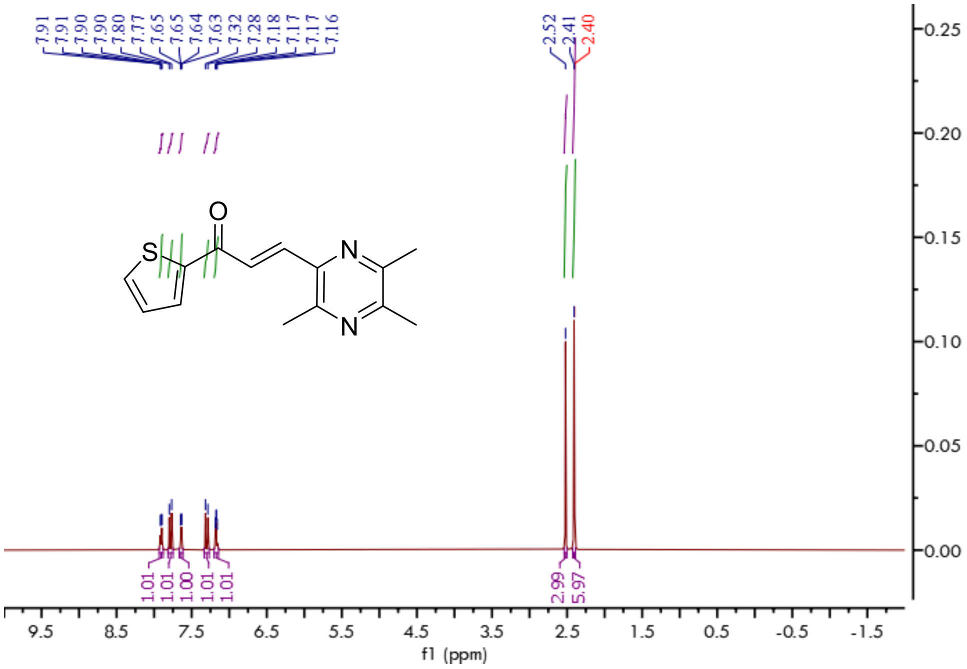

^{13}C NMR spectrum of **8e**

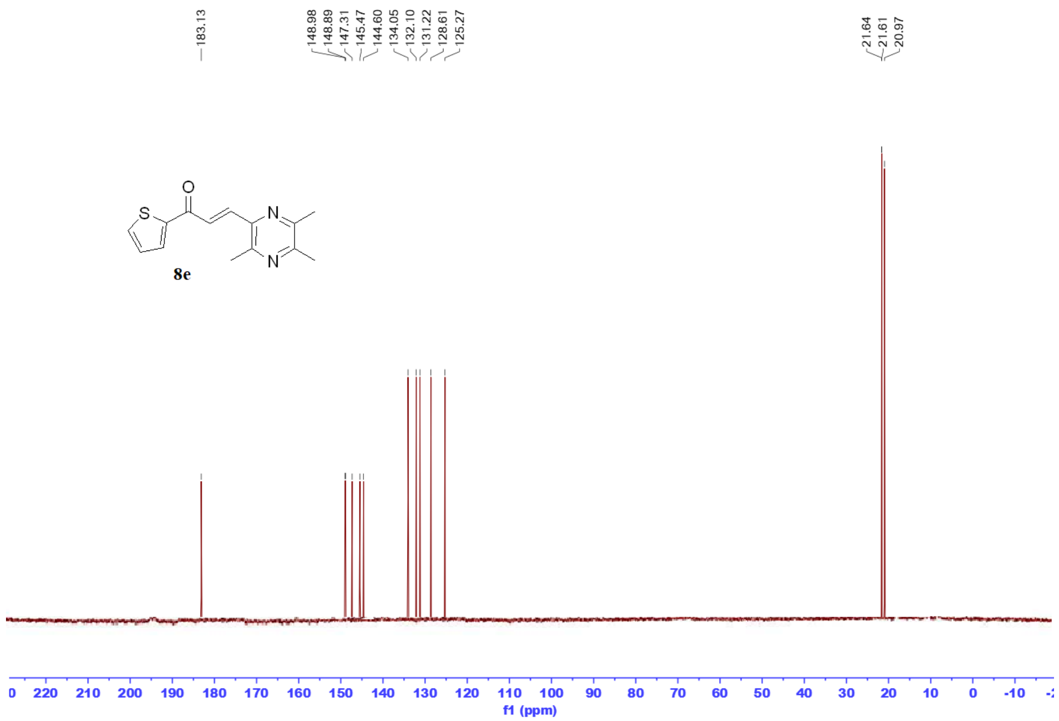

^{1}H NMR spectrum of **8f**

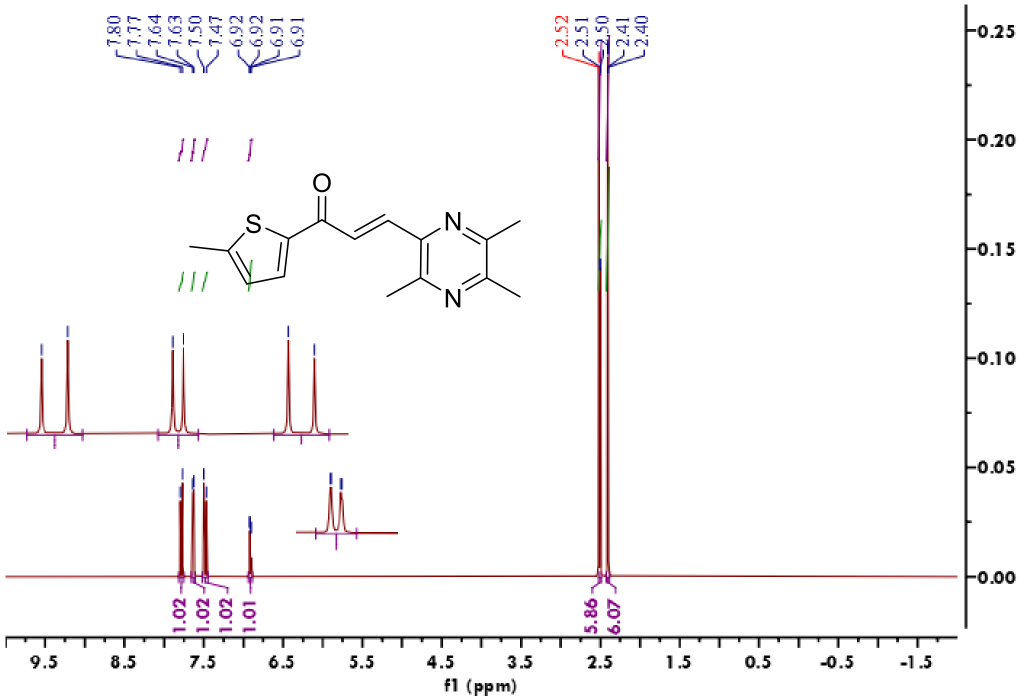

^{13}C NMR spectrum of **8f**

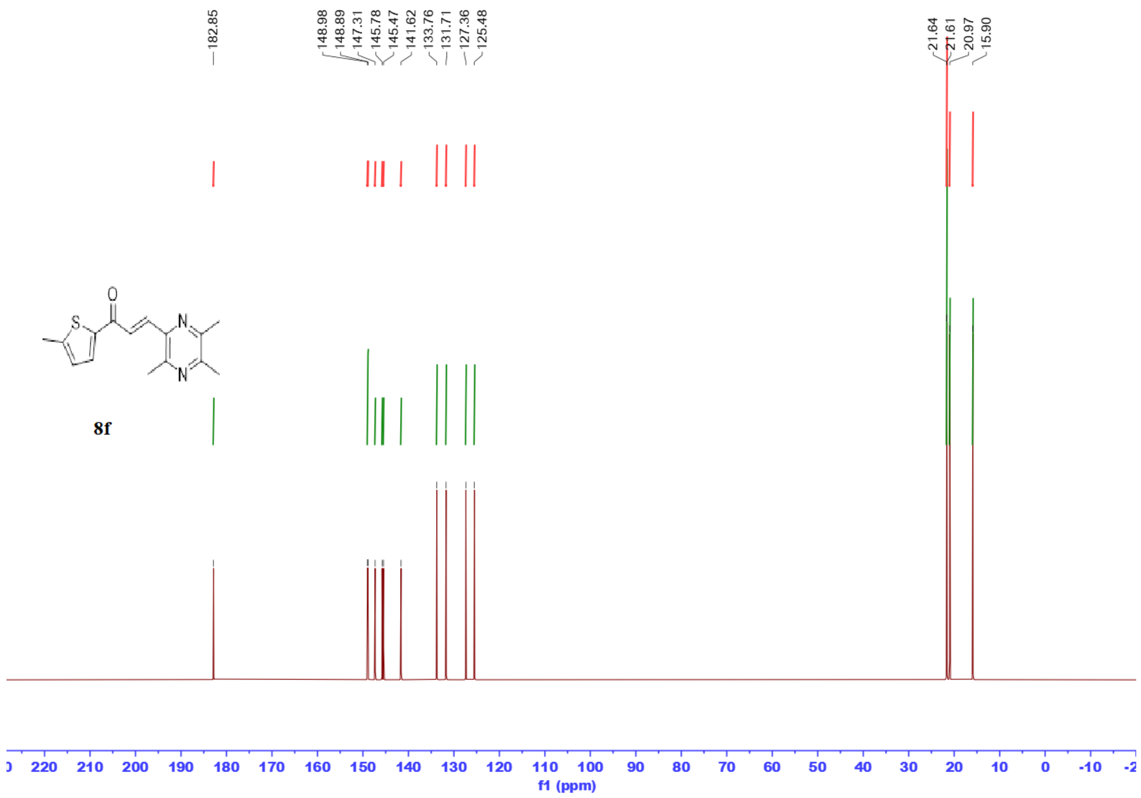

^{1}H NMR spectrum of **8g**

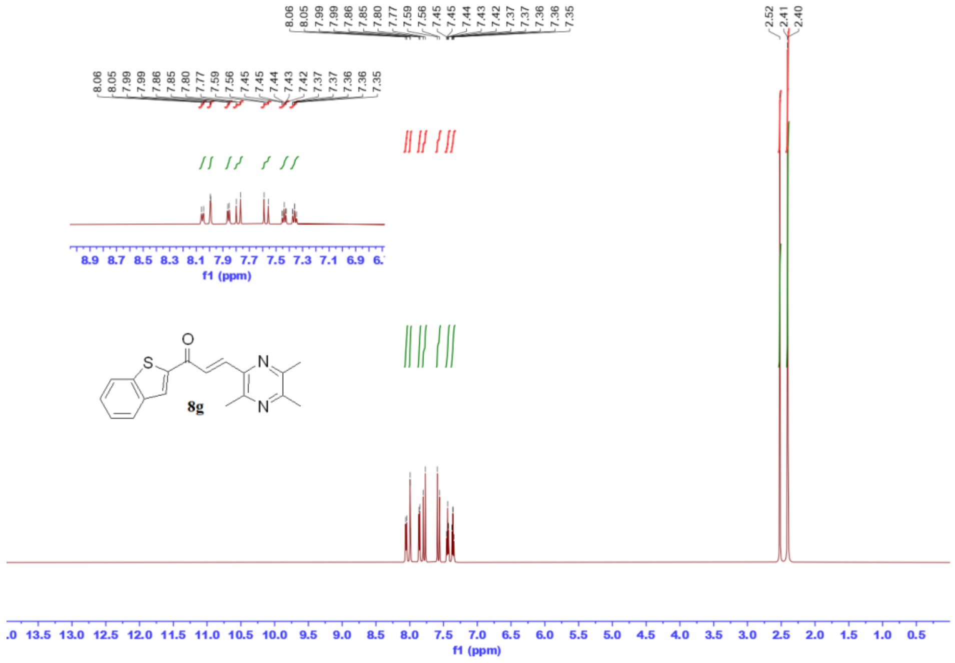

^{13}C NMR spectrum of **8g**

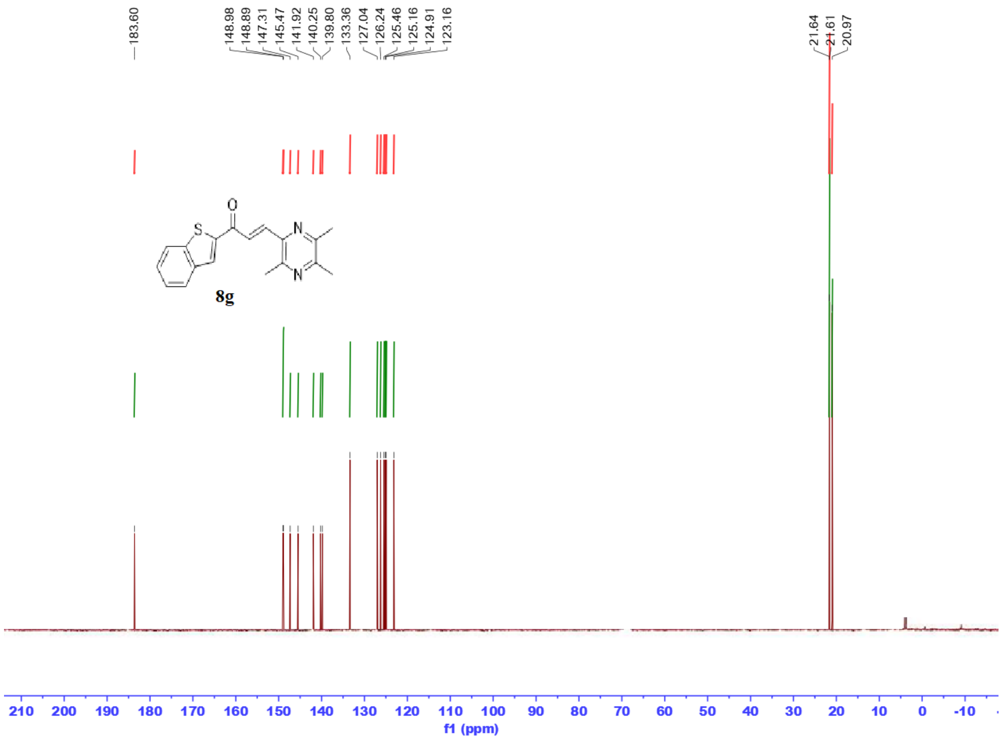

^{1}H NMR spectrum of **9a**

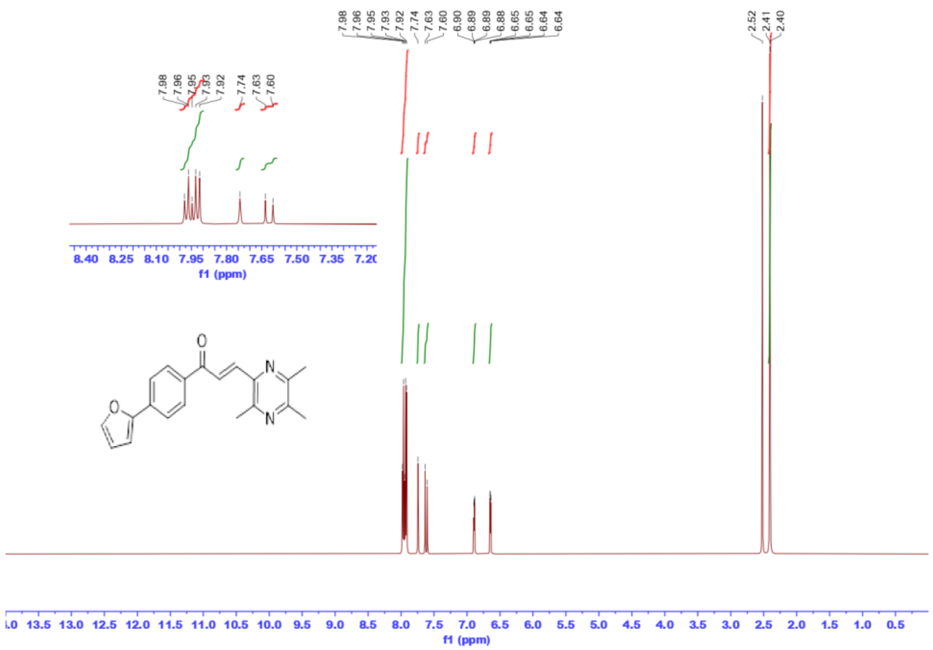

^{13}C NMR spectrum of **9a**

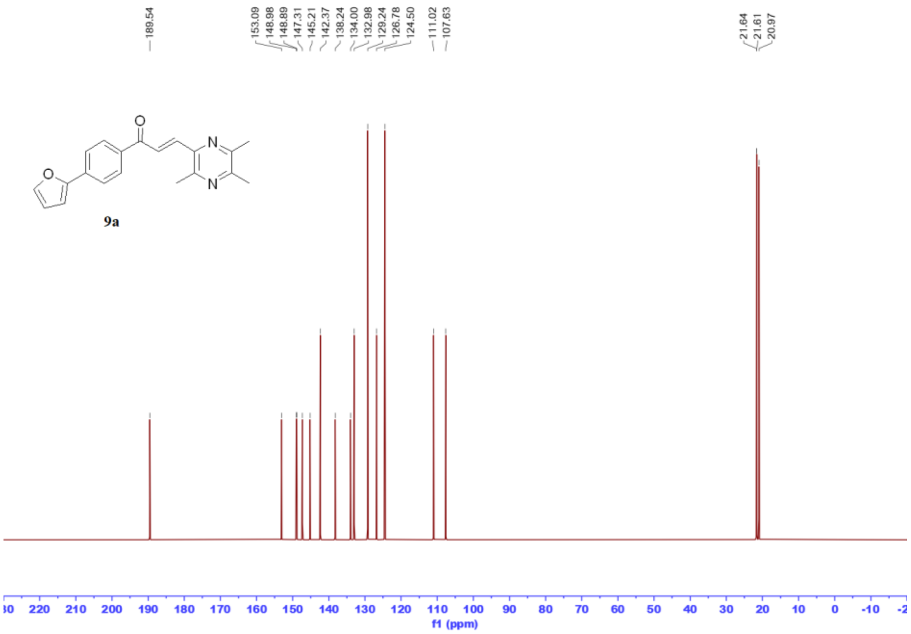

^{1}H NMR spectrum of **9b**

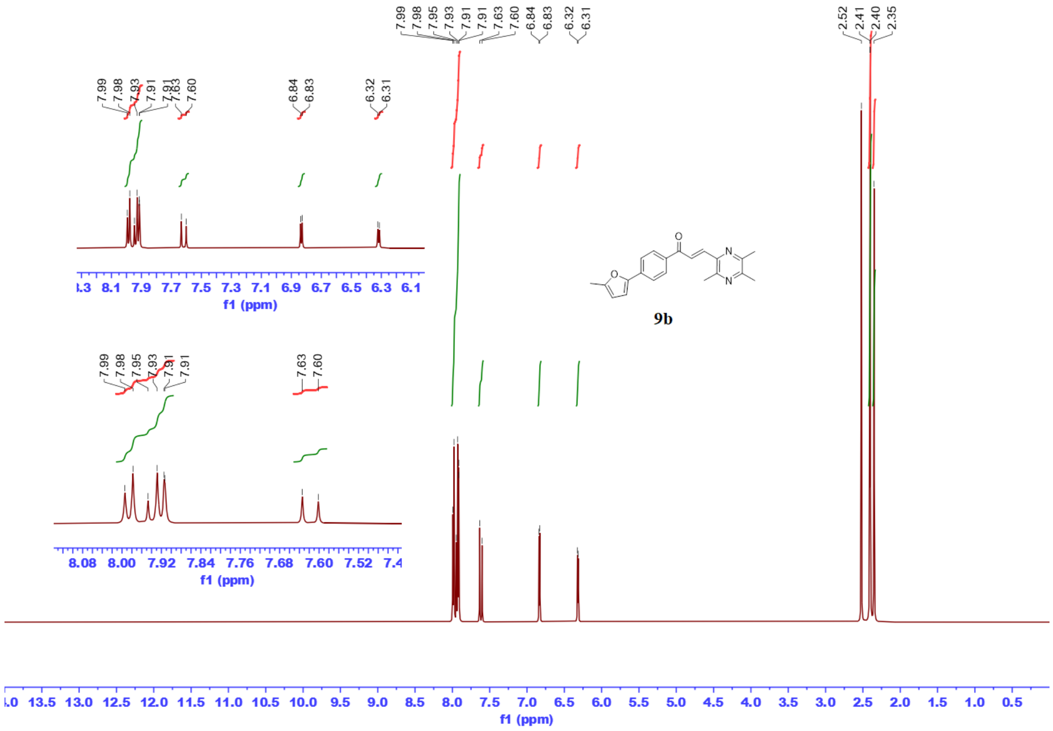

^{13}C NMR spectrum of **9b**

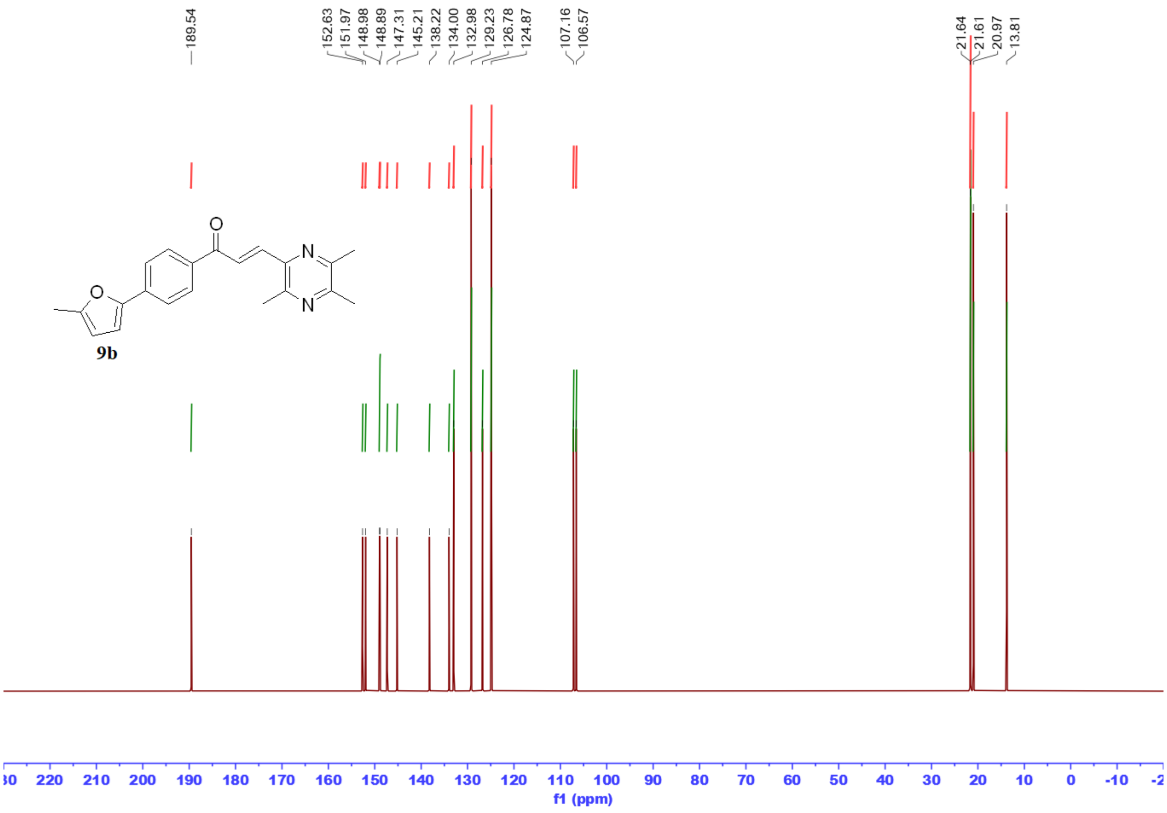

^{1}H NMR spectrum of **9c**

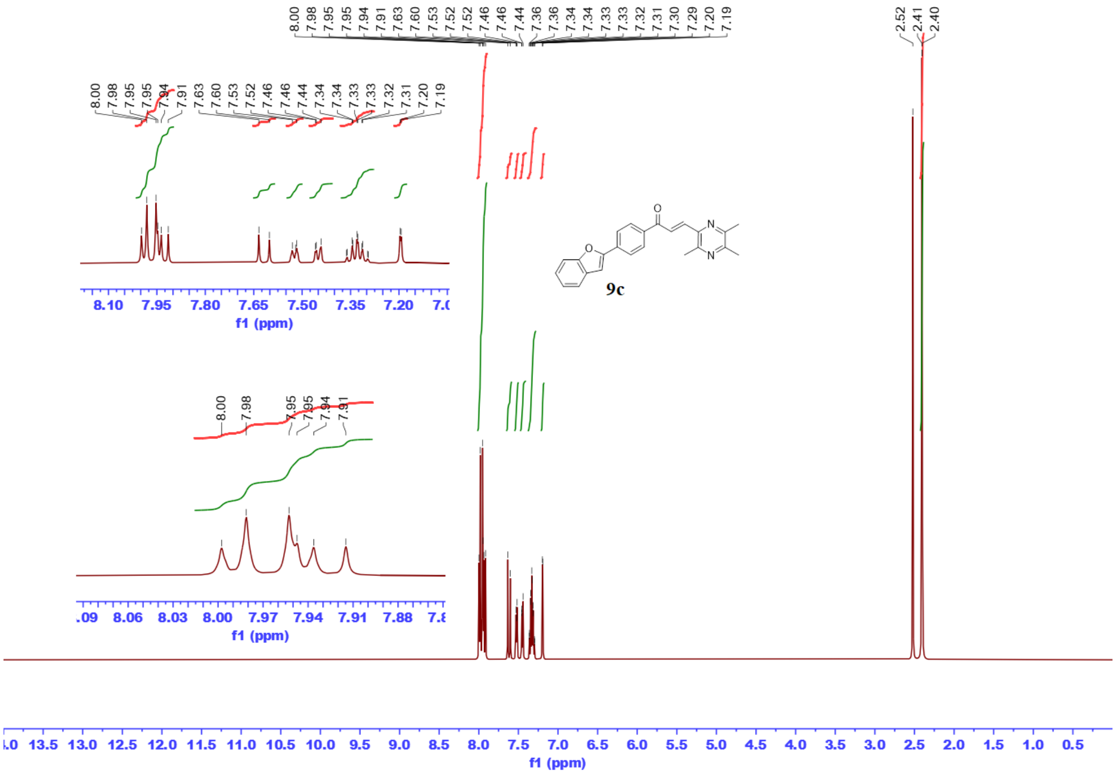

^{13}C NMR spectrum of **9c**

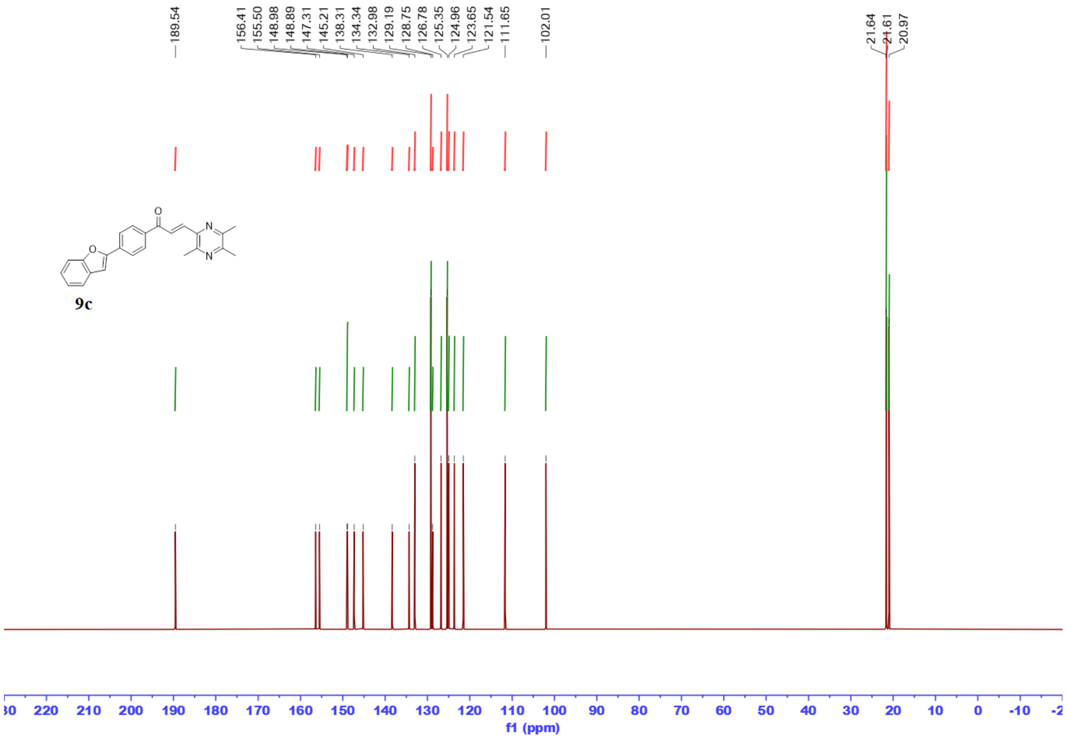

^{1}H NMR spectrum of **9d**

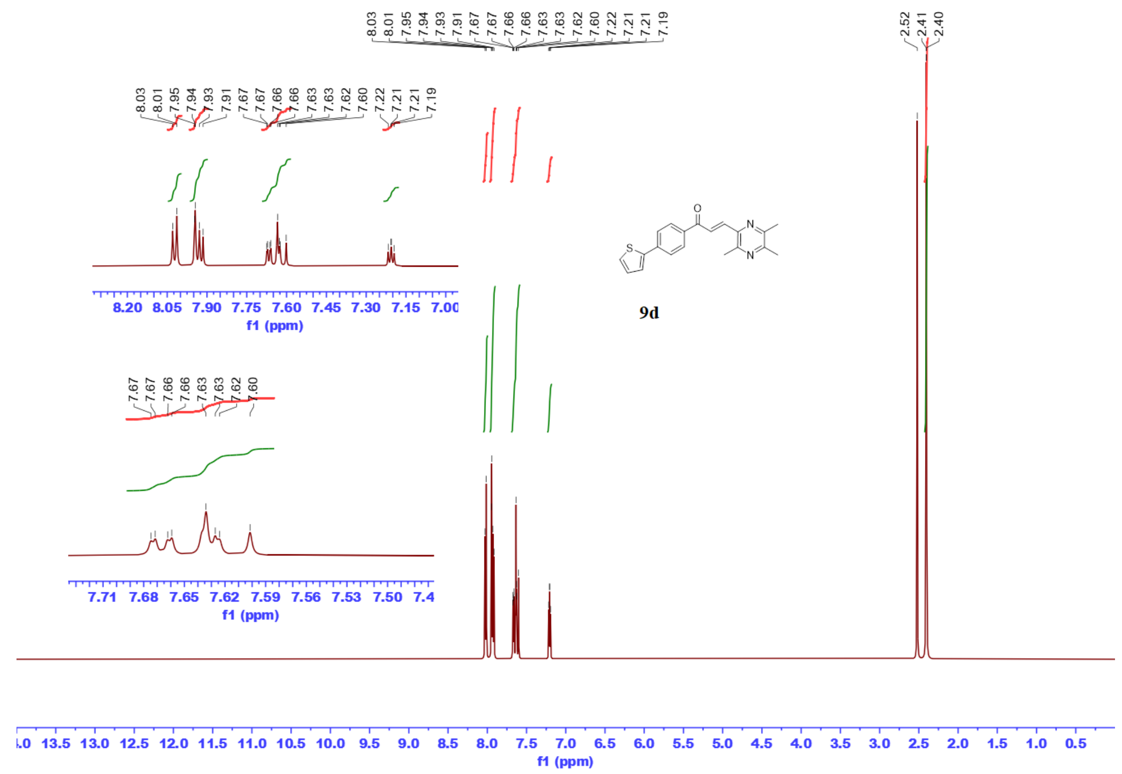

^{13}C NMR spectrum of **9d**

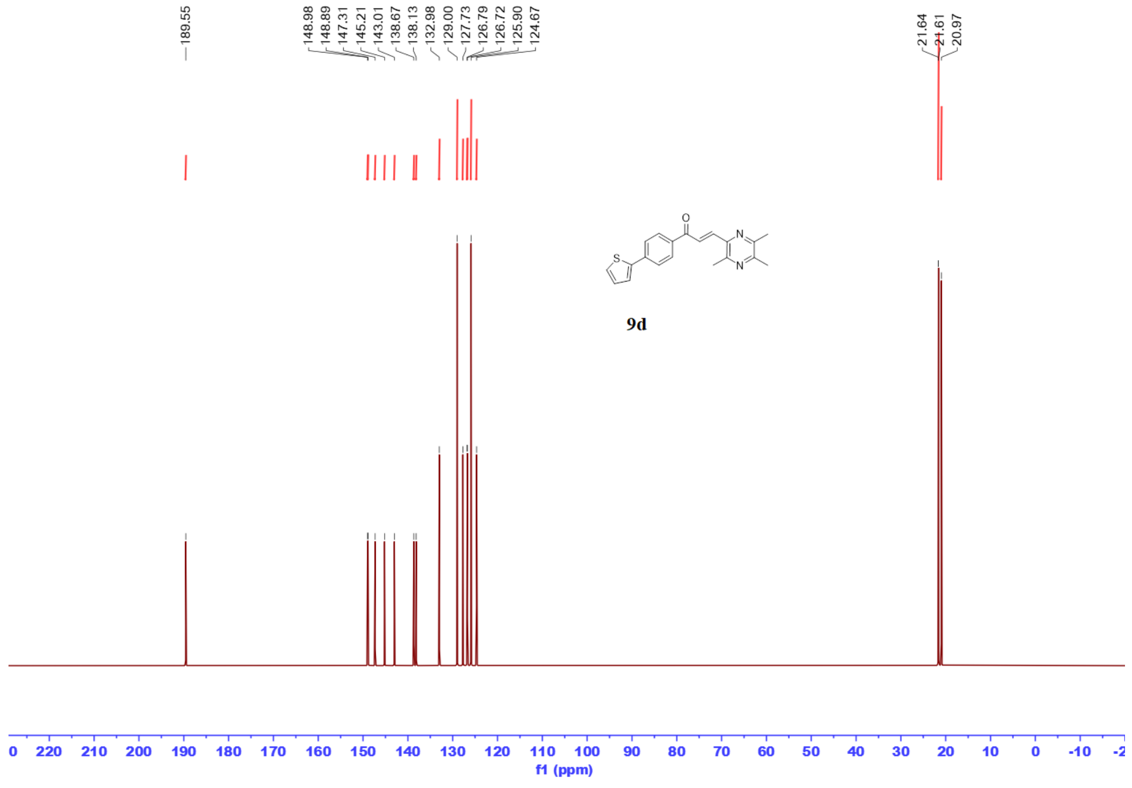

^{1}H NMR spectrum of **9e**

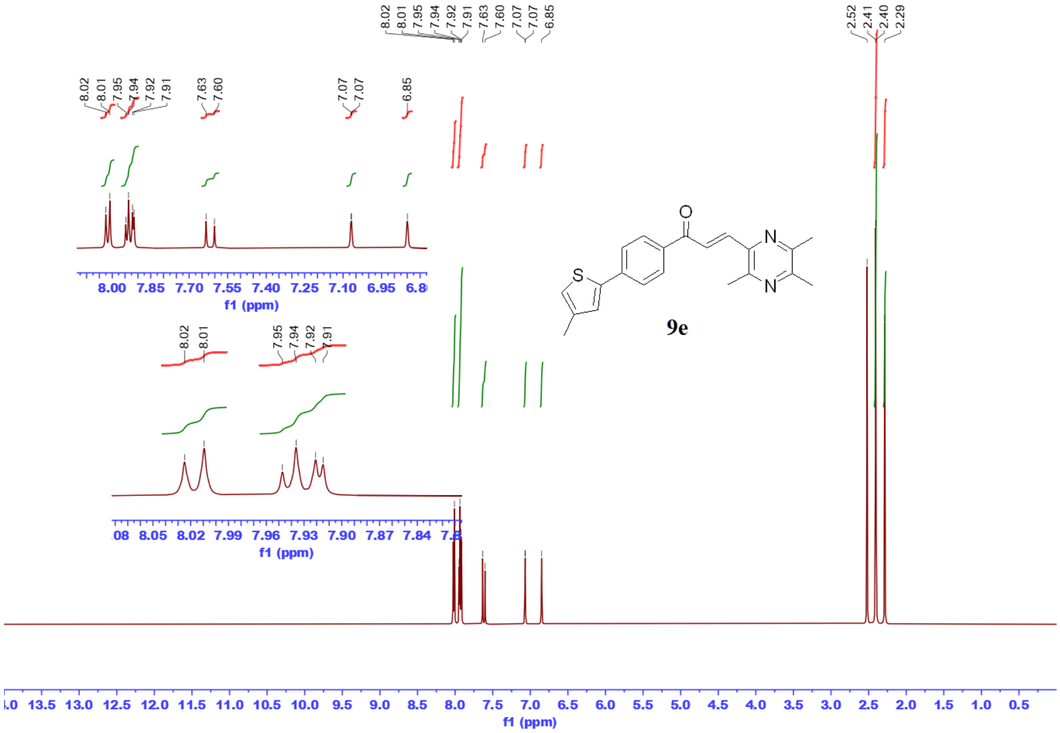

^{13}C NMR spectrum of **9e**

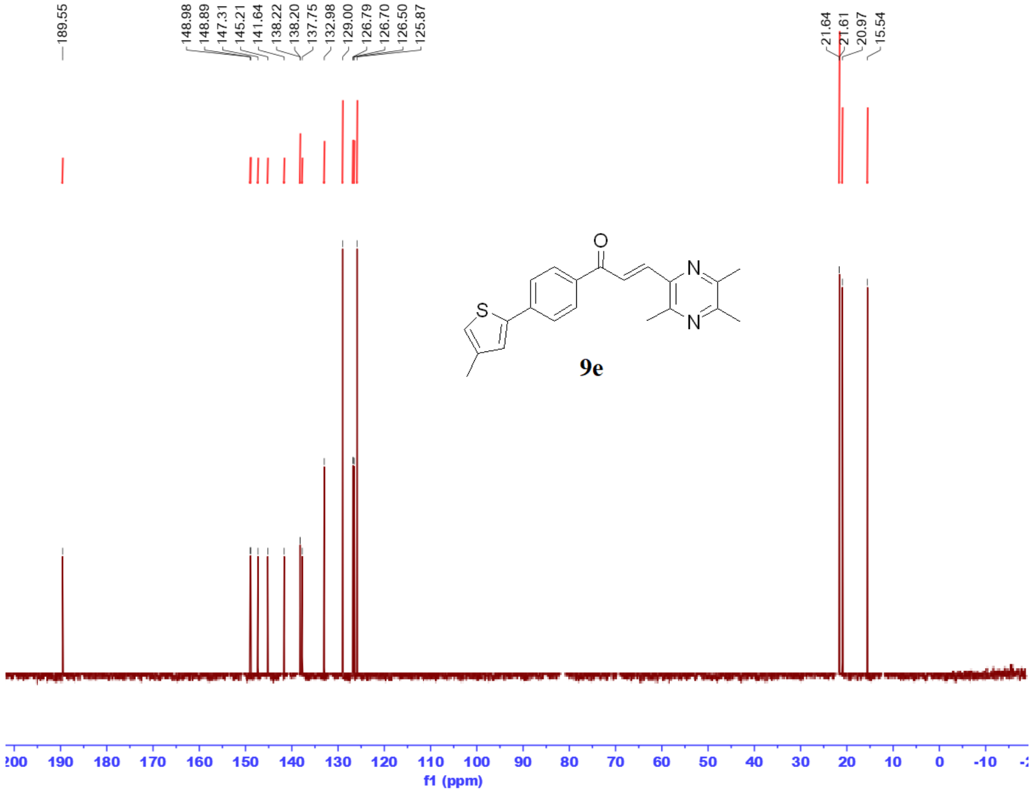

^{1}H NMR spectrum of **9f**

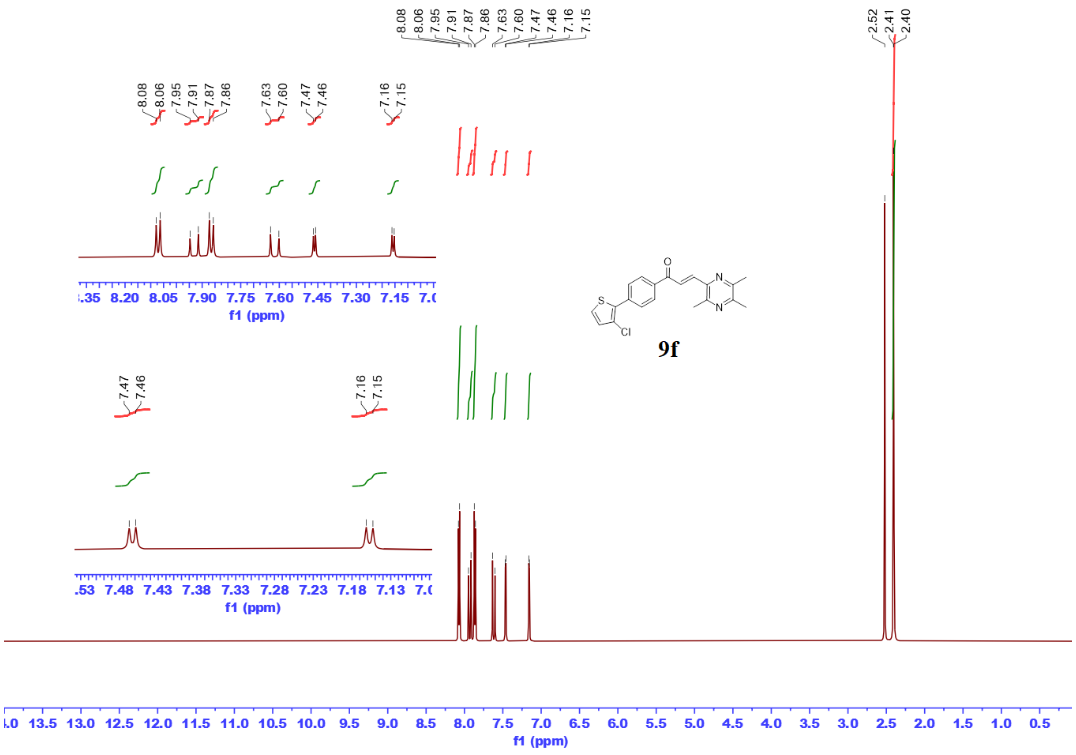

^{13}C NMR spectrum of **9f**

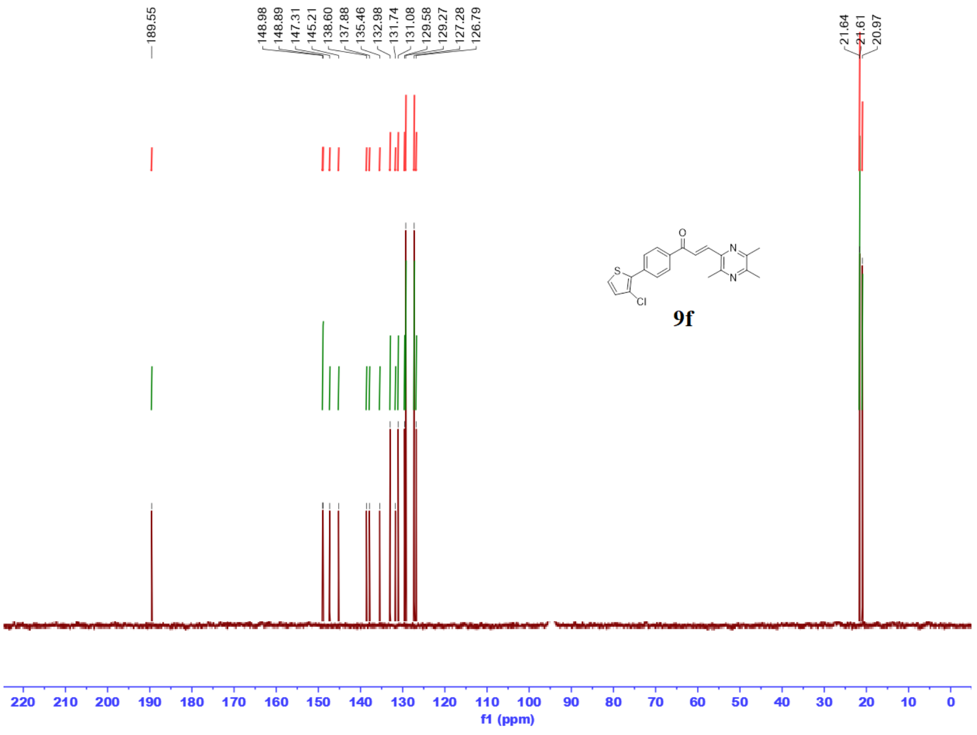

^{1}H NMR spectrum of **9g**

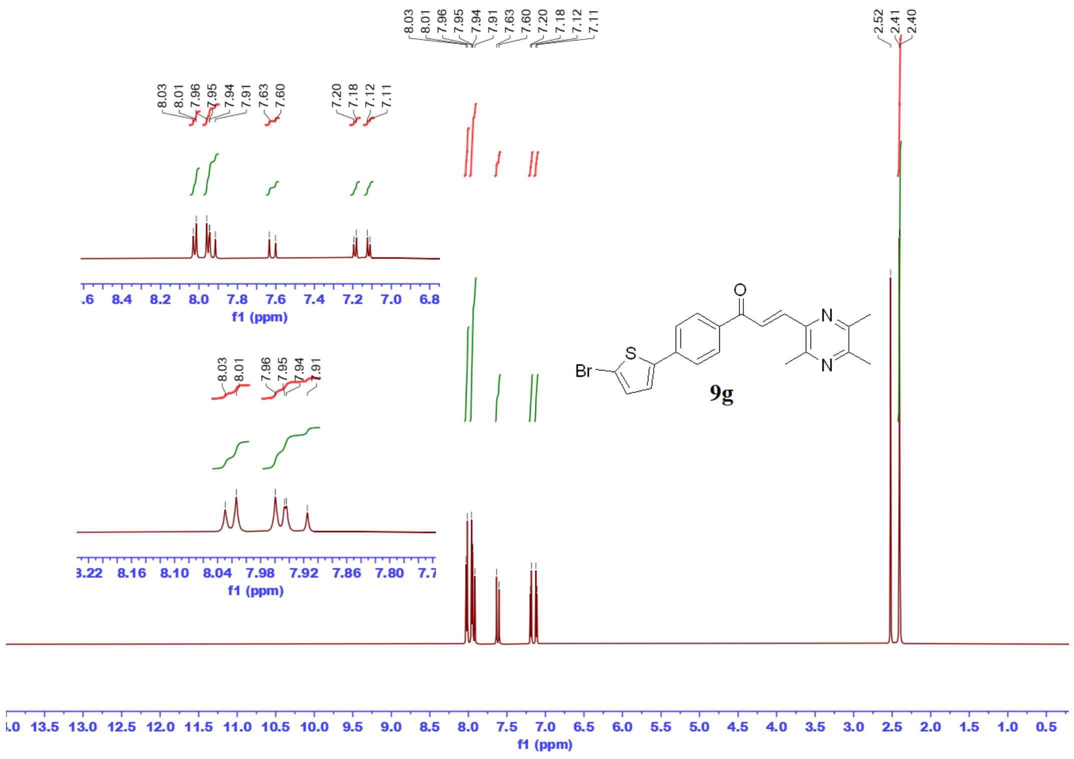

^{13}C NMR spectrum of **9g**

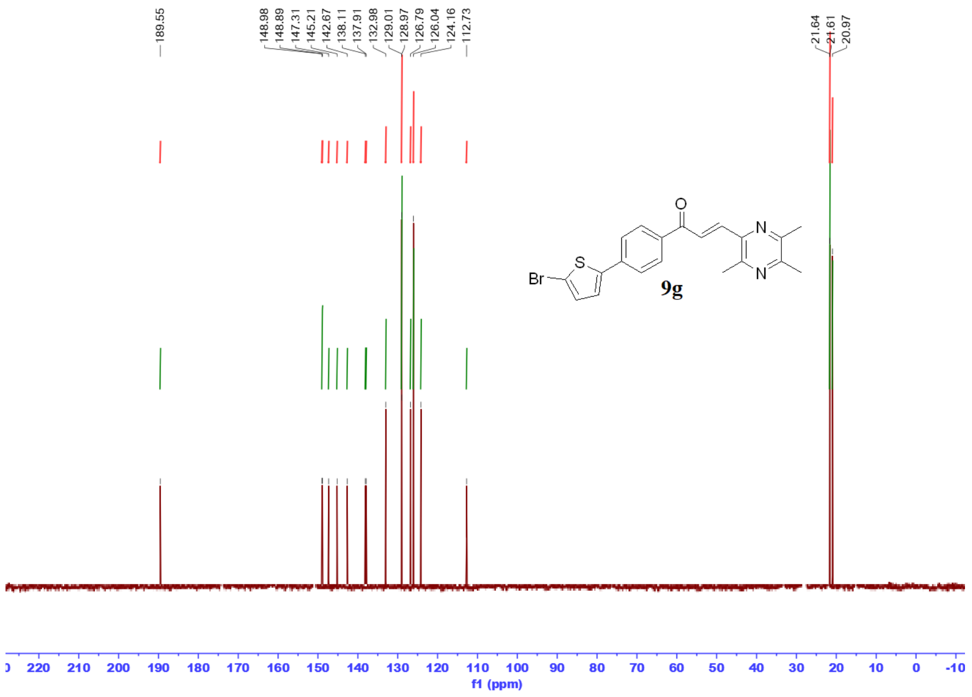

^{1}H NMR spectrum of **10a**

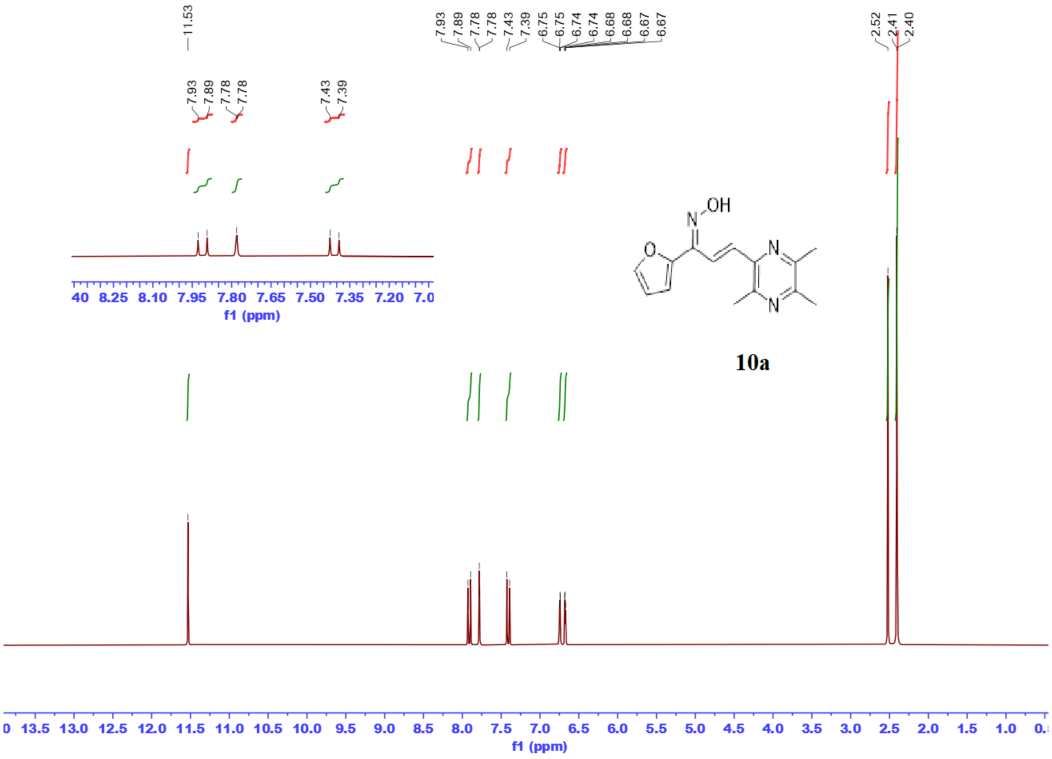

^{13}C NMR spectrum of **10a**

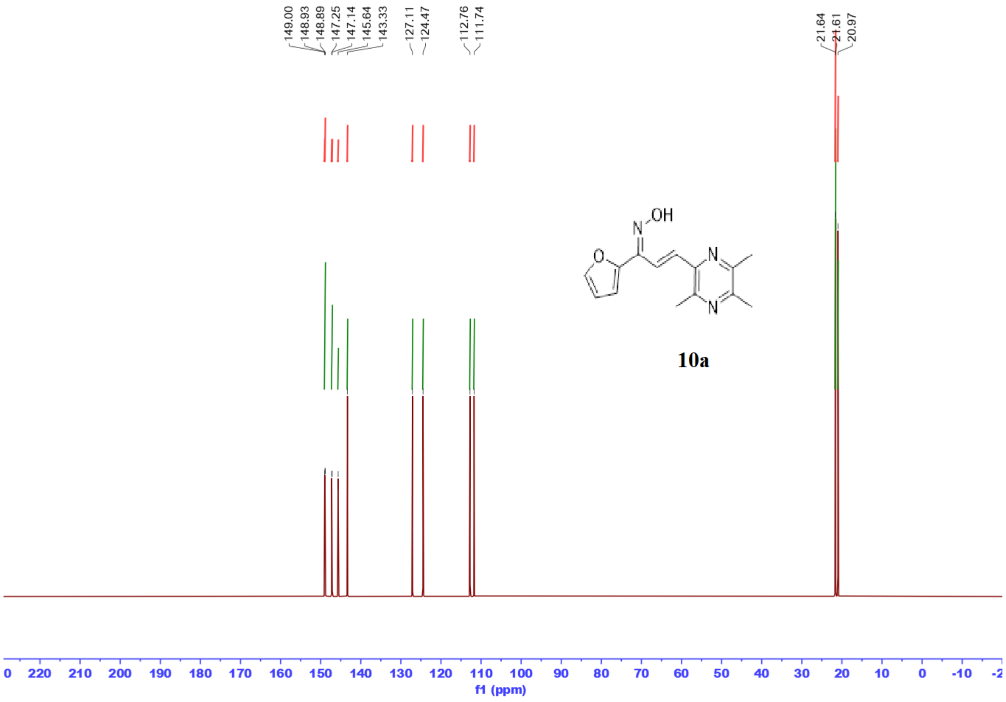

^{1}H NMR spectrum of **10b**

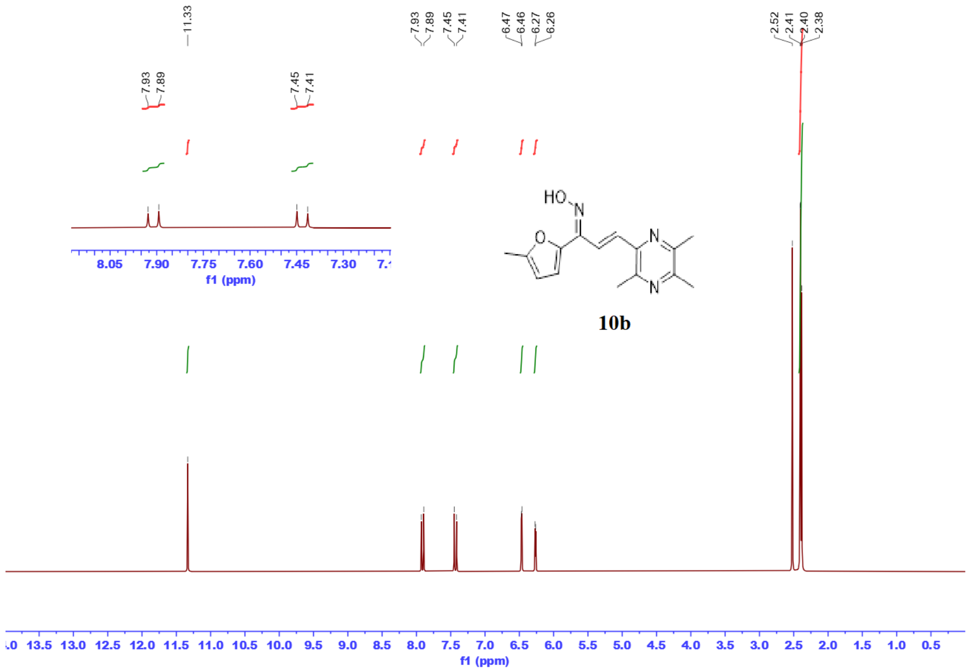

^{13}C NMR spectrum of **10b**

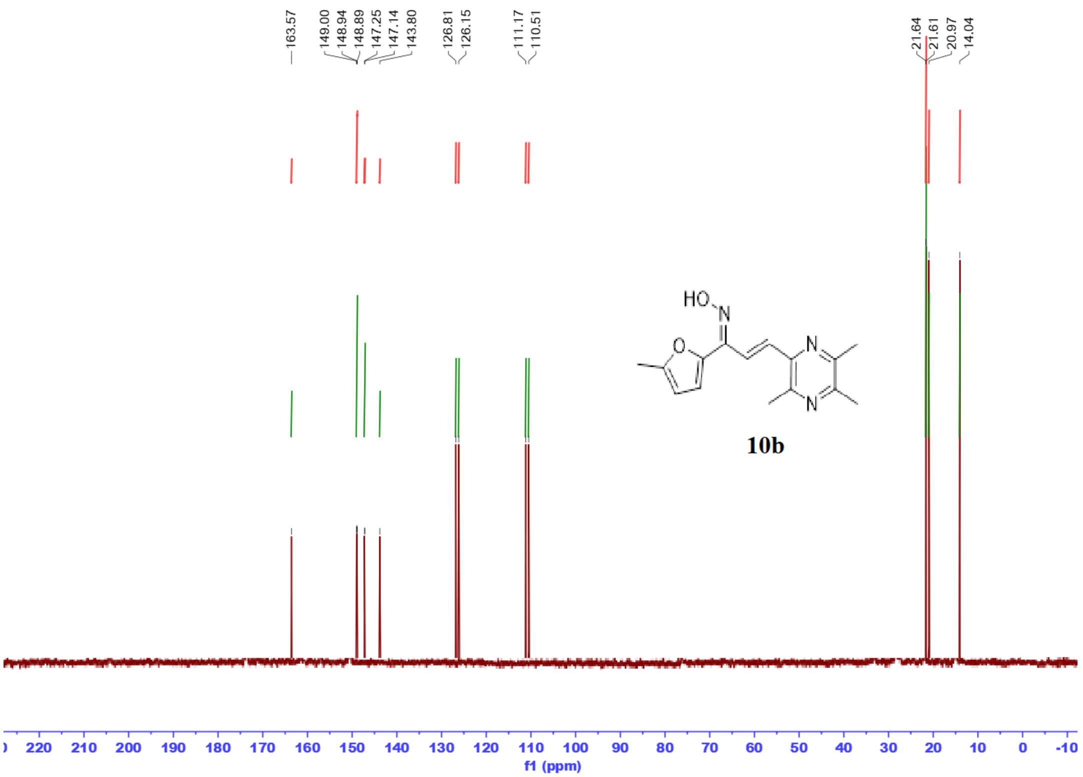

^{1}H NMR spectrum of **10c**

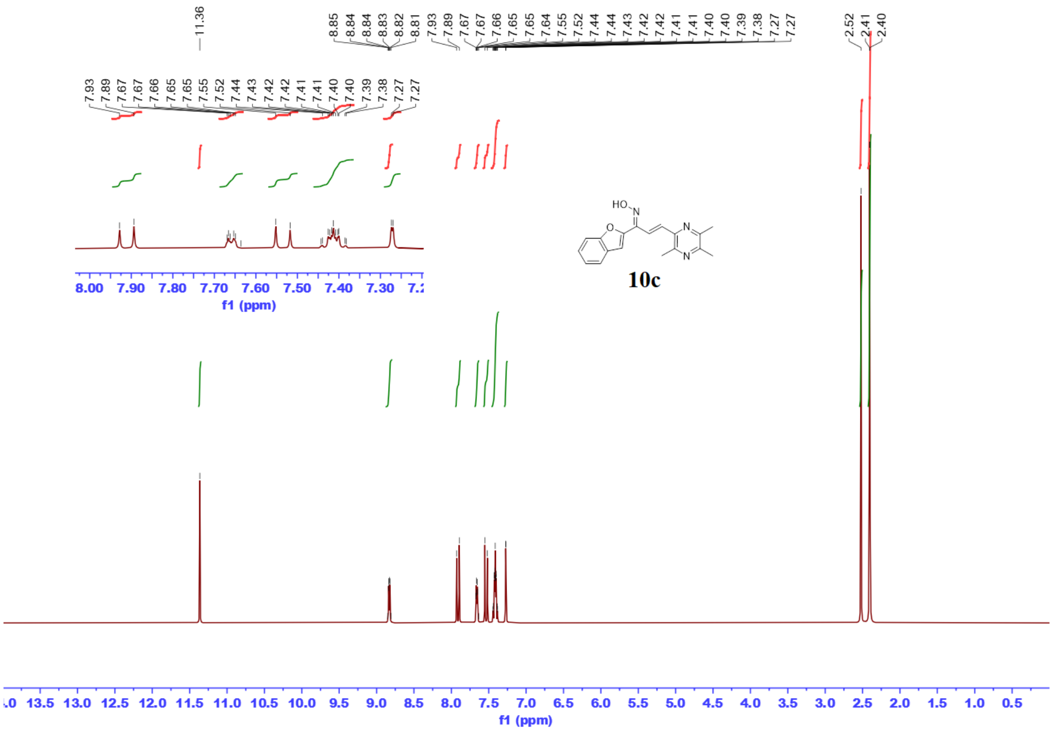

^{13}C NMR spectrum of **10c**

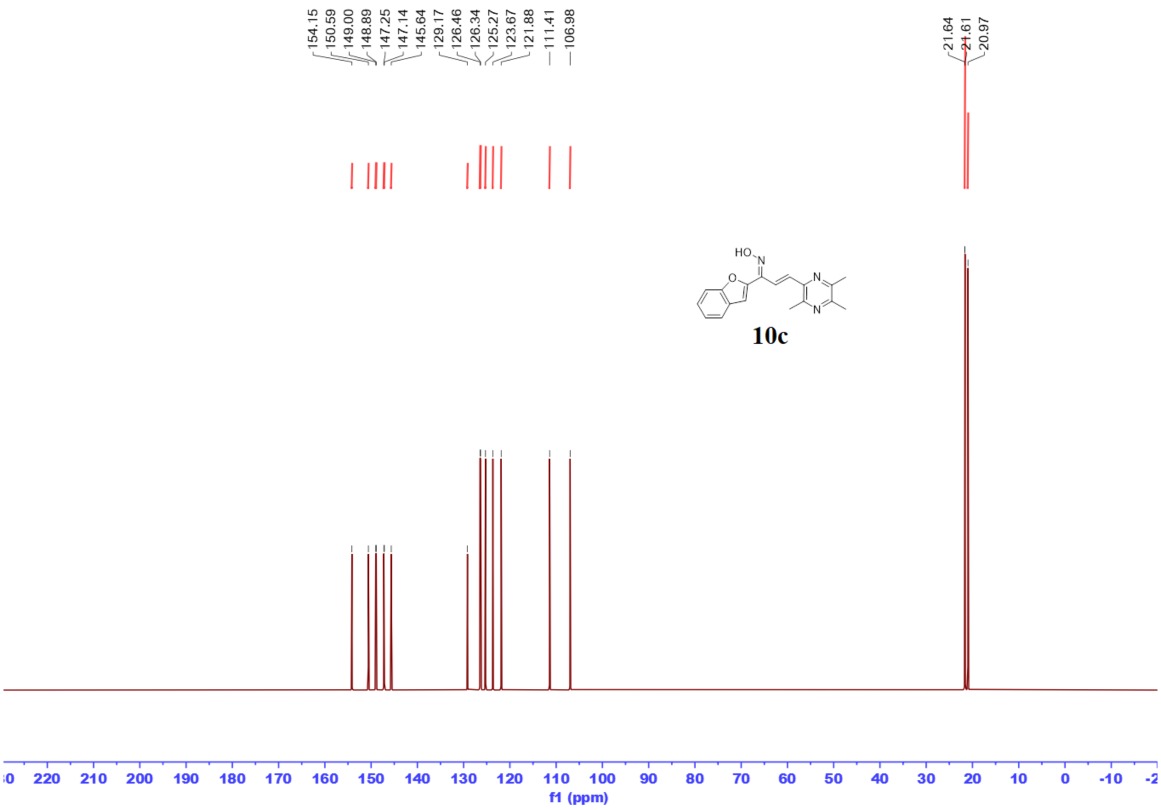

^{1}H NMR spectrum of **10d**

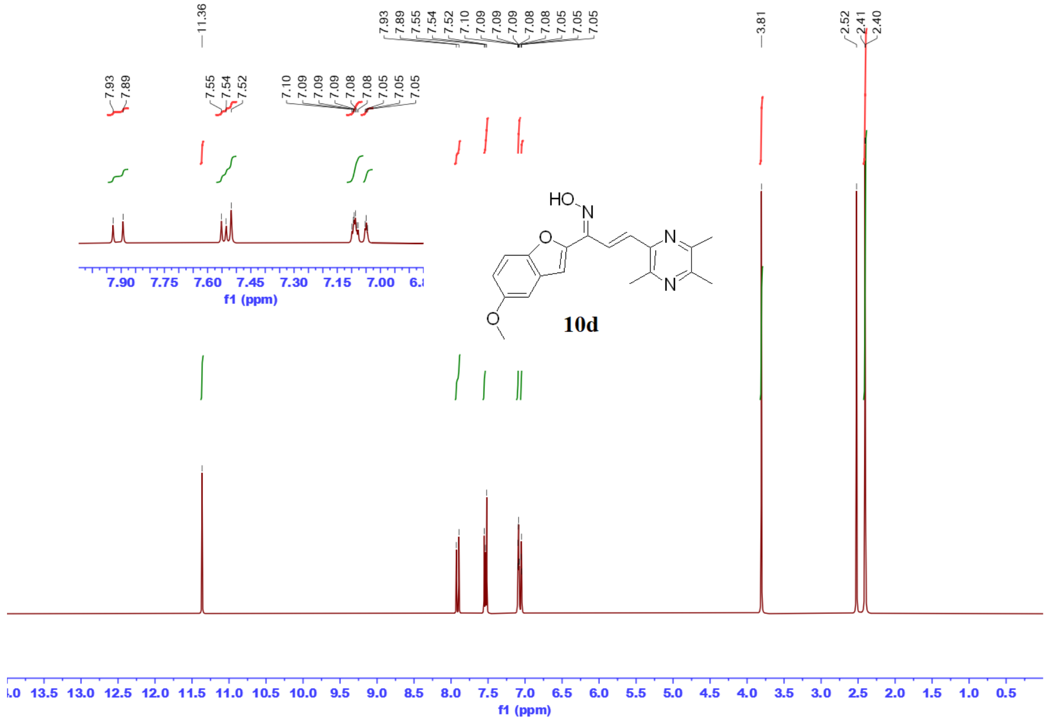

^{13}C NMR spectrum of **10d**

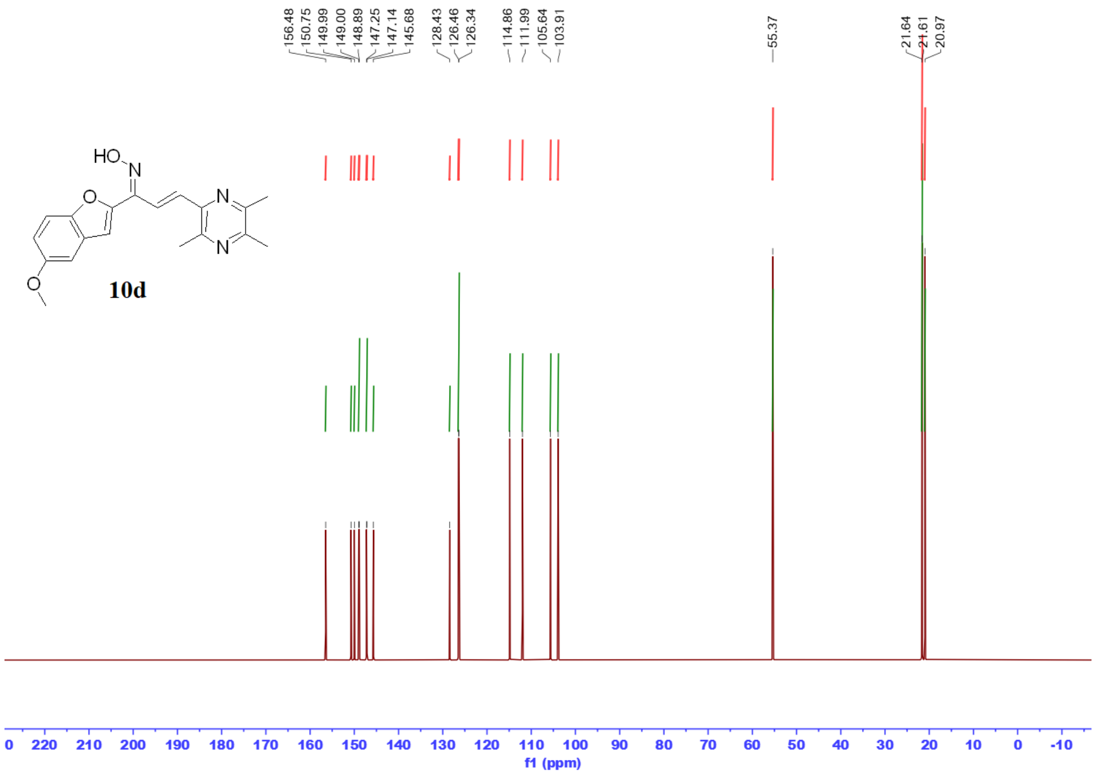

^{1}H NMR spectrum of **10e**

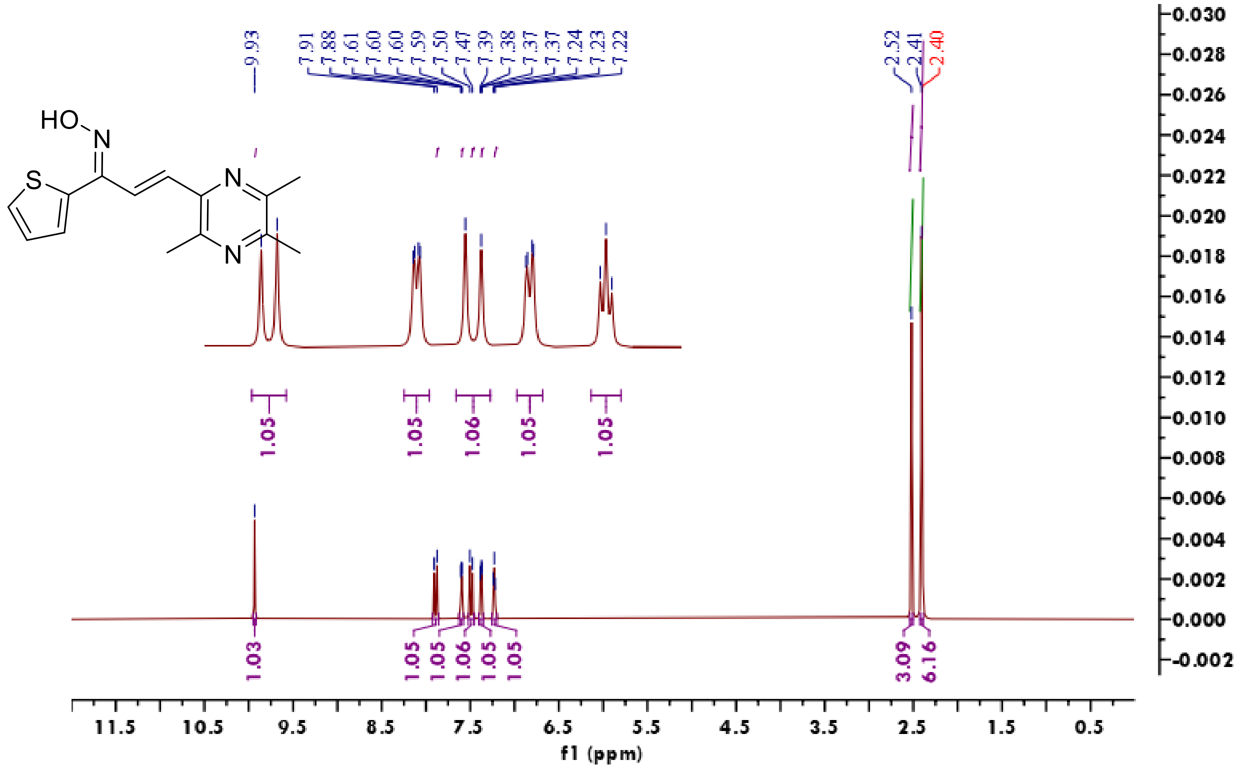

^{13}C NMR spectrum of **10e**

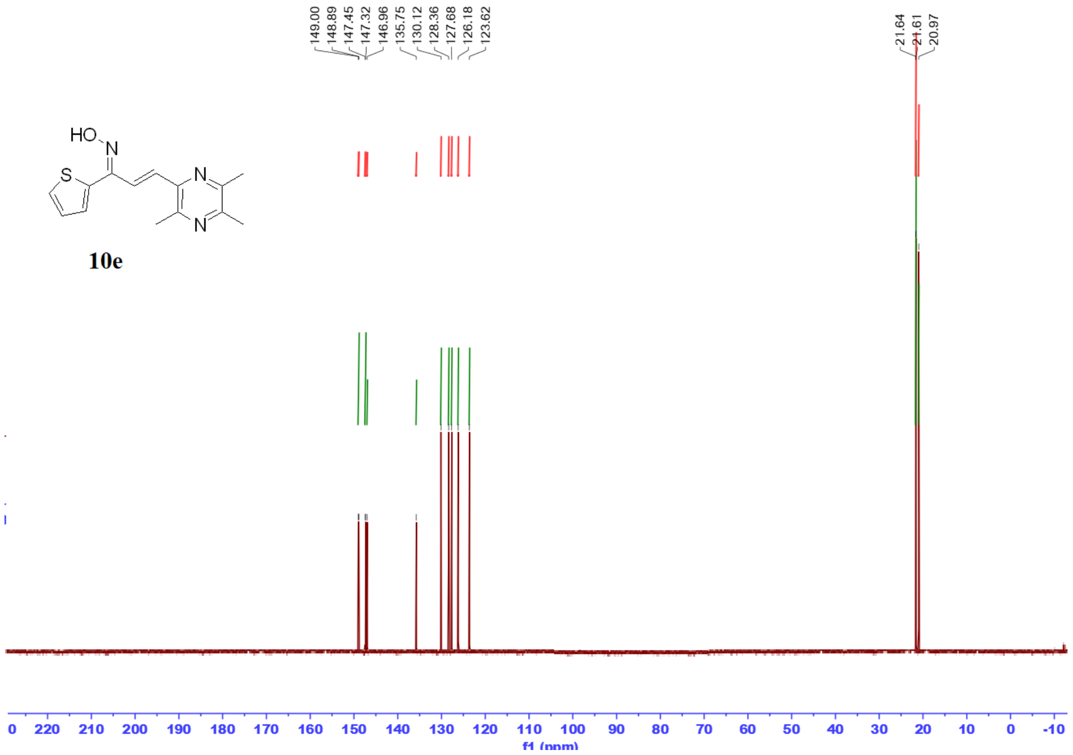

^{1}H NMR spectrum of **10f**

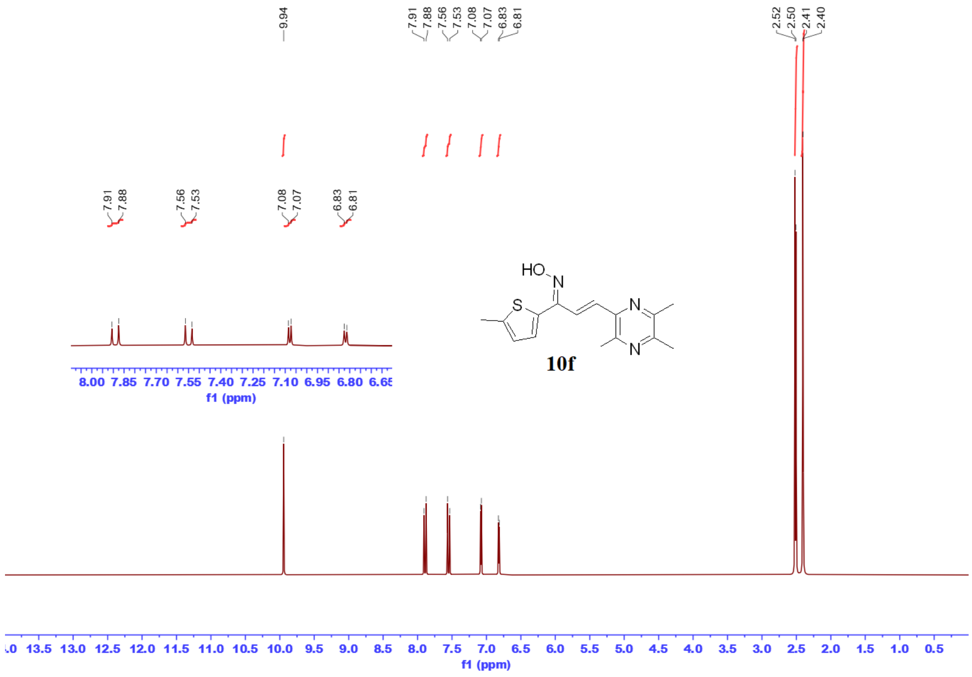

^{13}C NMR spectrum of **10f**

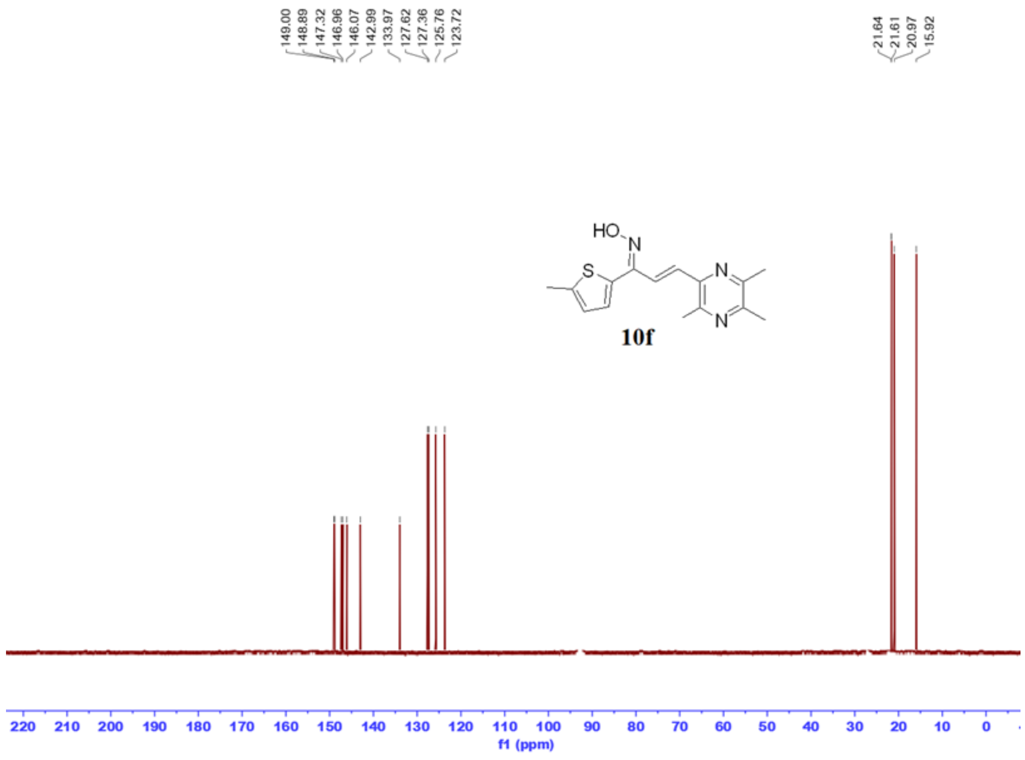

^{1}H NMR spectrum of **10g**

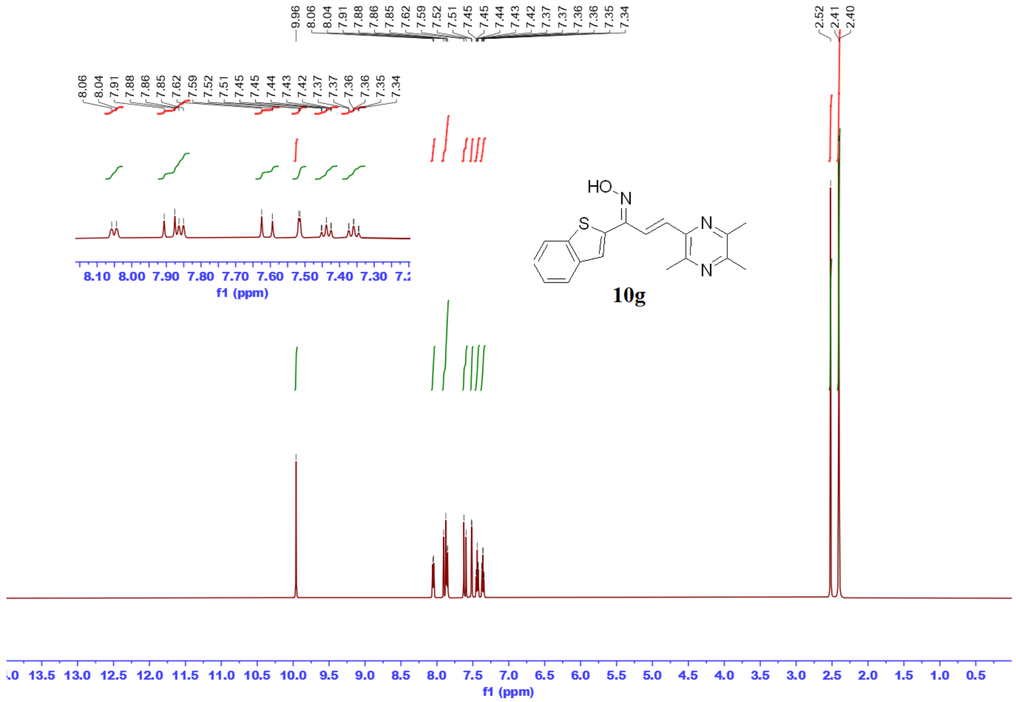

^{13}C NMR spectrum of **10g**

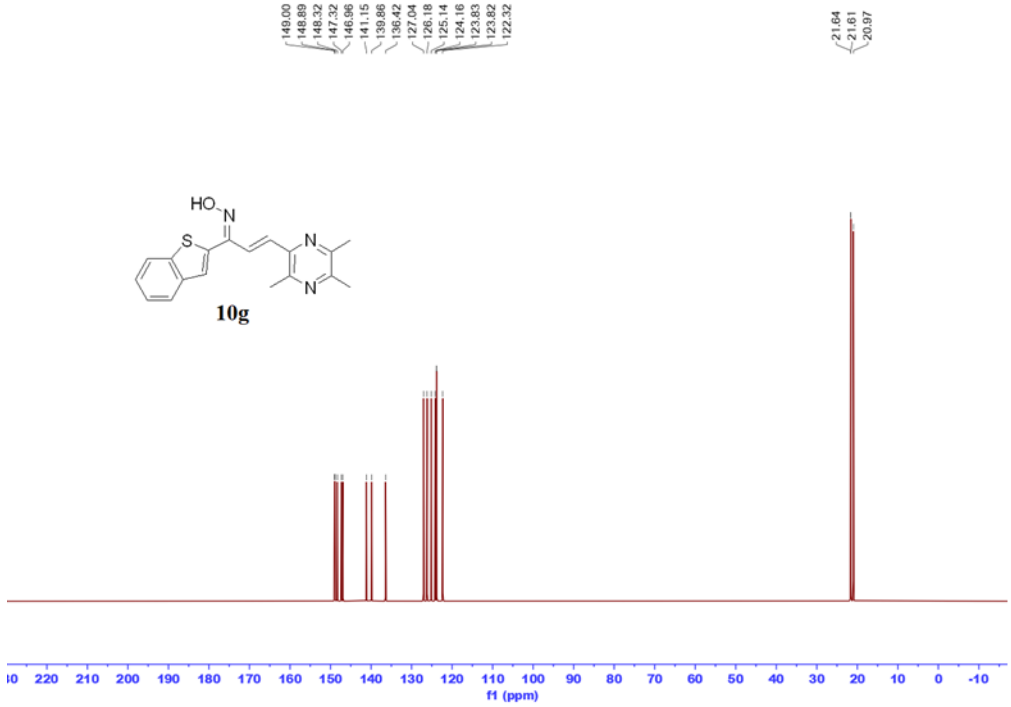

^{1}H NMR spectrum of **11a**

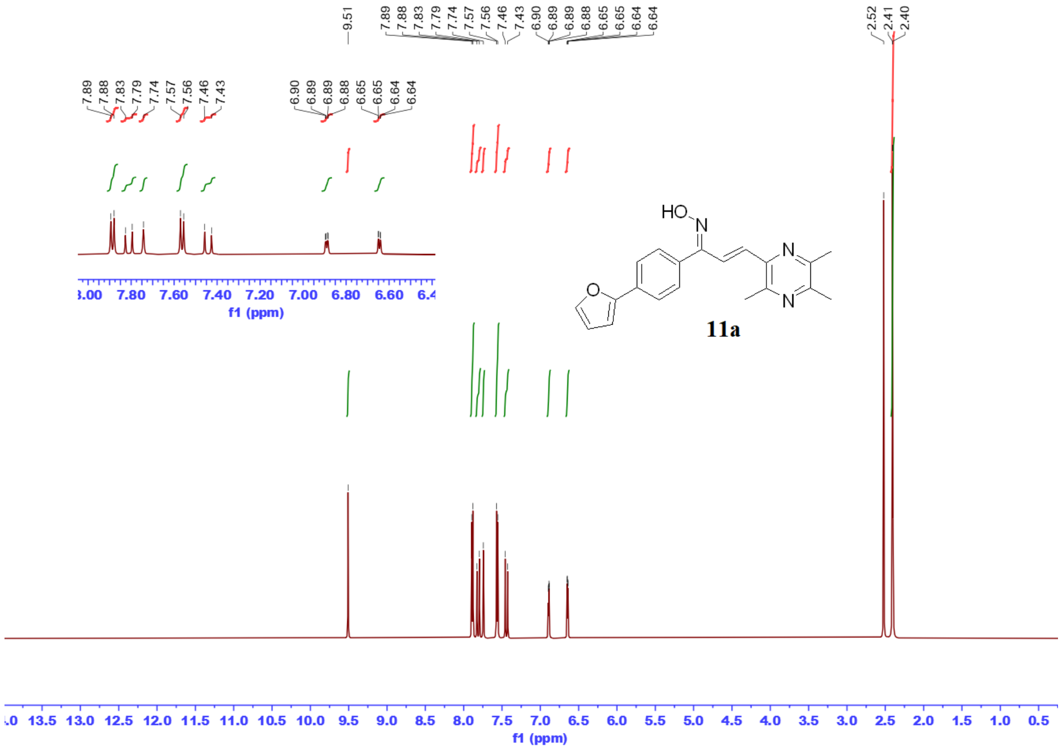

^{13}C NMR spectrum of **11a**

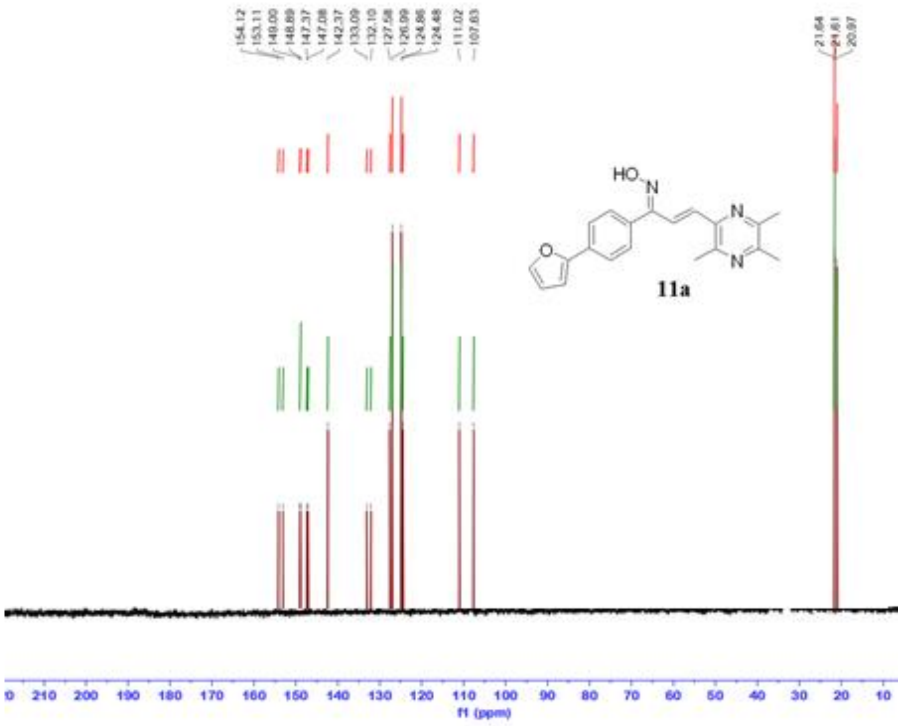

^{1}H NMR spectrum of **11b**

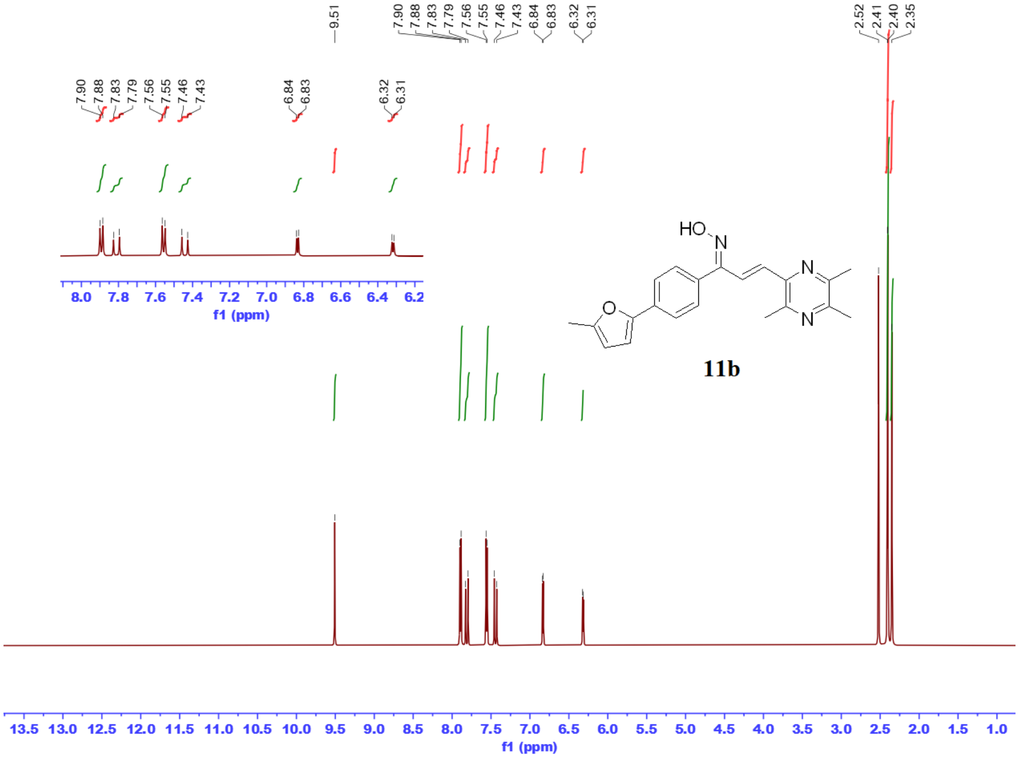

^{13}C NMR spectrum of **11b**

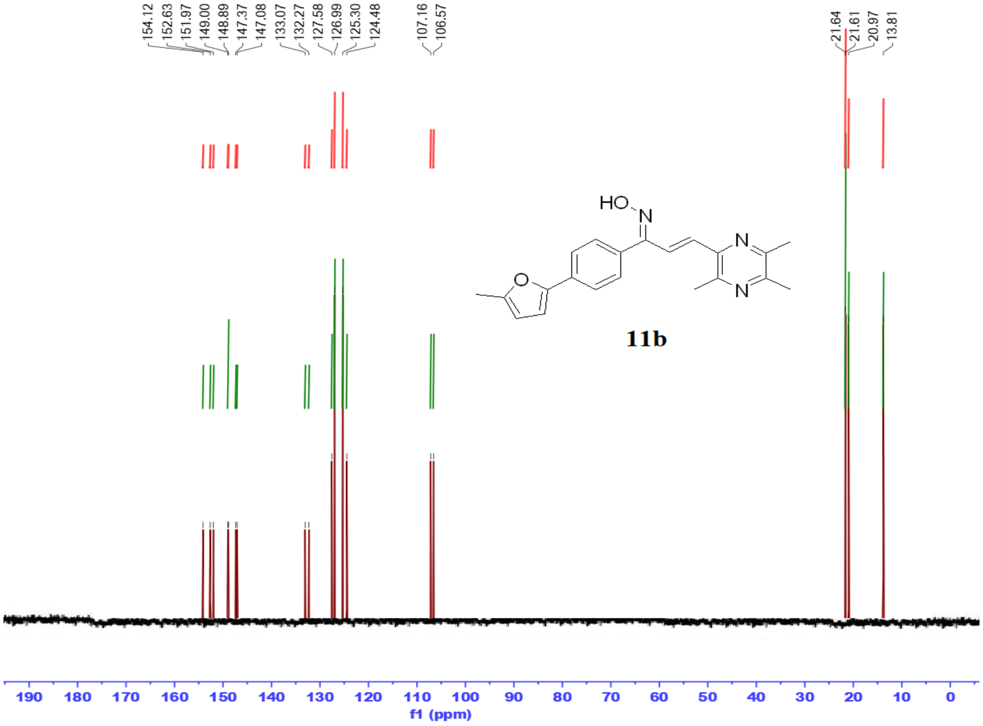

^{1}H NMR spectrum of **11c**

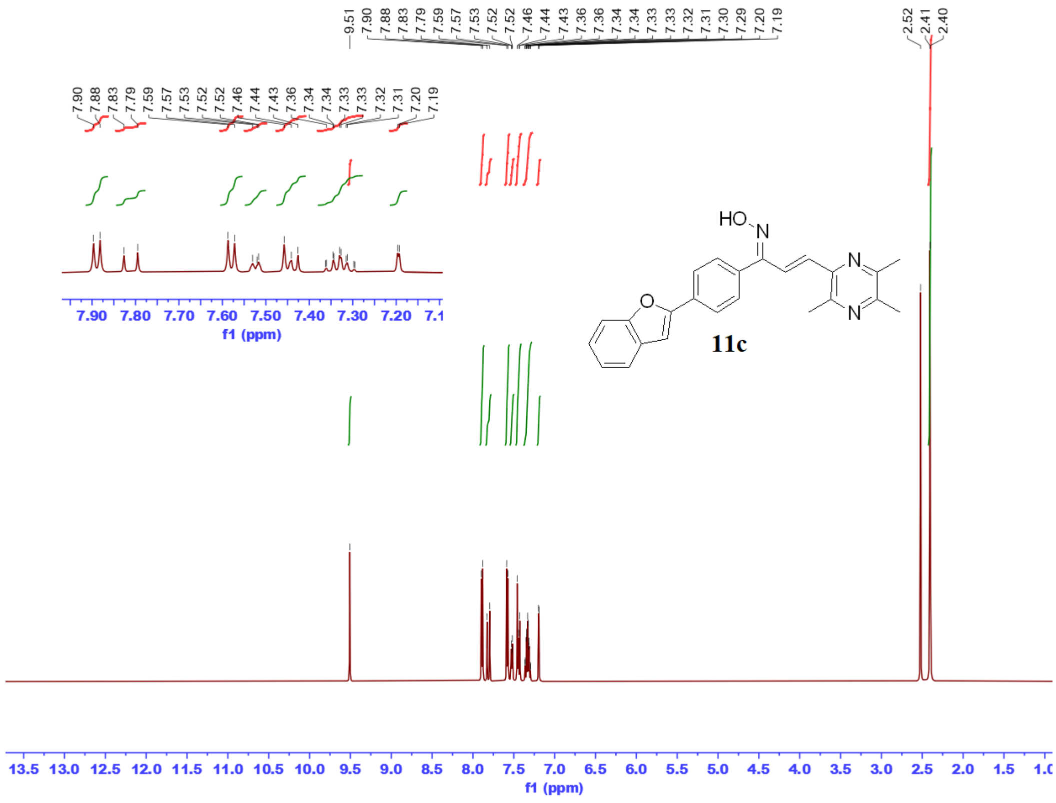

^{13}C NMR spectrum of **11c**

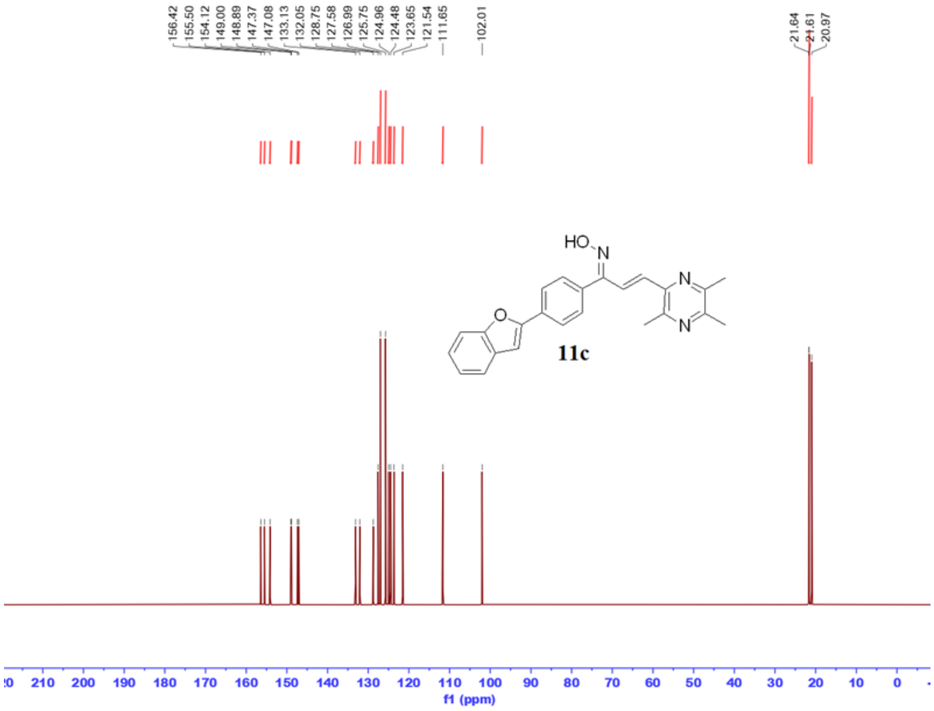

^{1}H NMR spectrum of **11d**

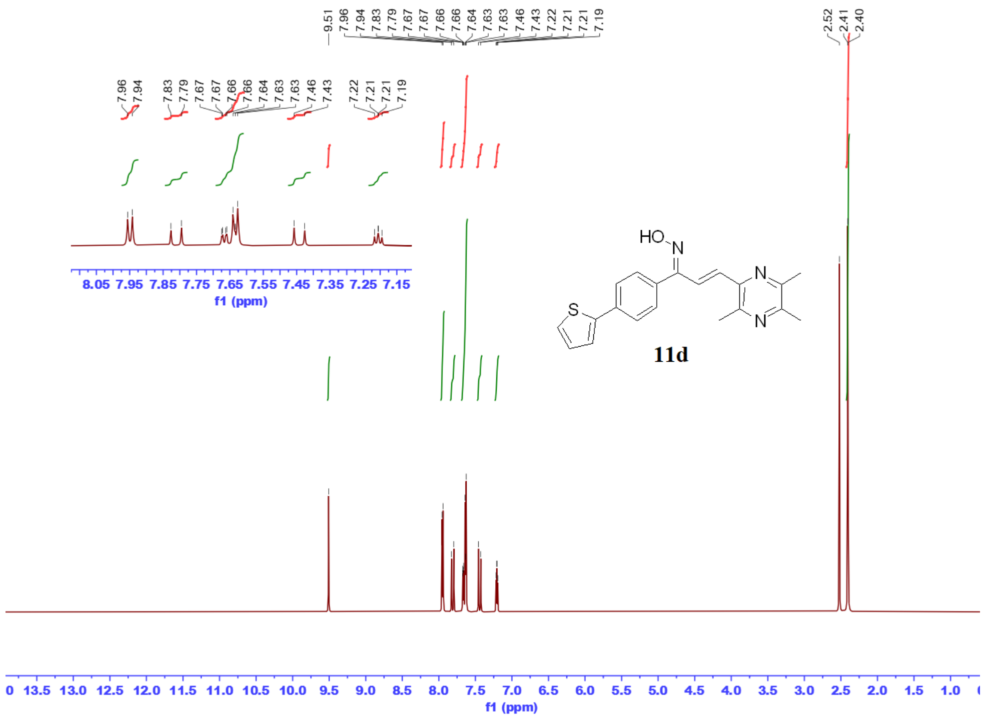

^{13}C NMR spectrum of **11d**

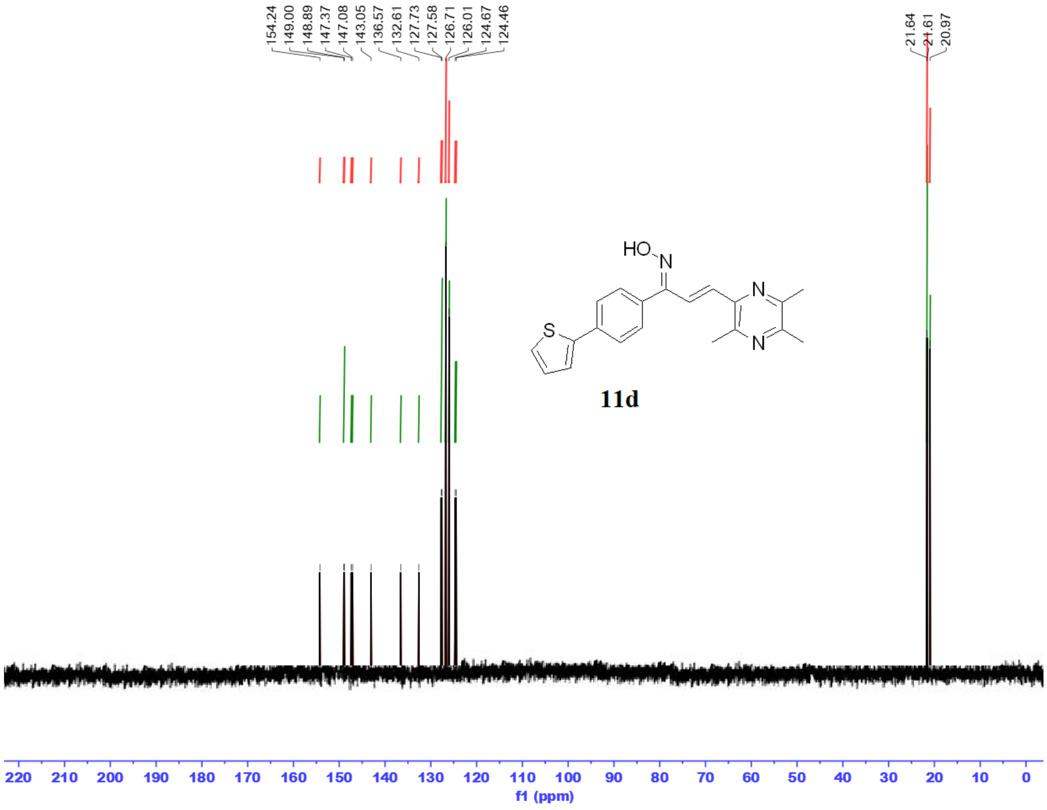

^{1}H NMR spectrum of **11e**

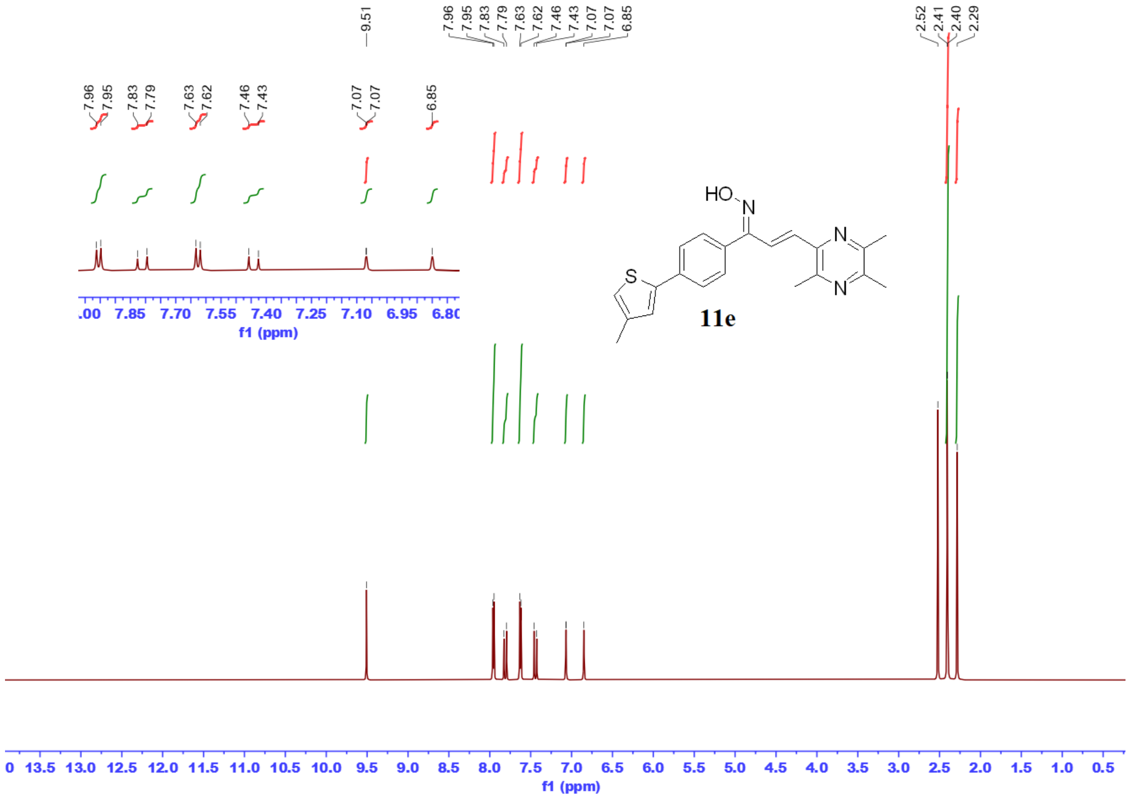

^{13}C NMR spectrum of **11e**

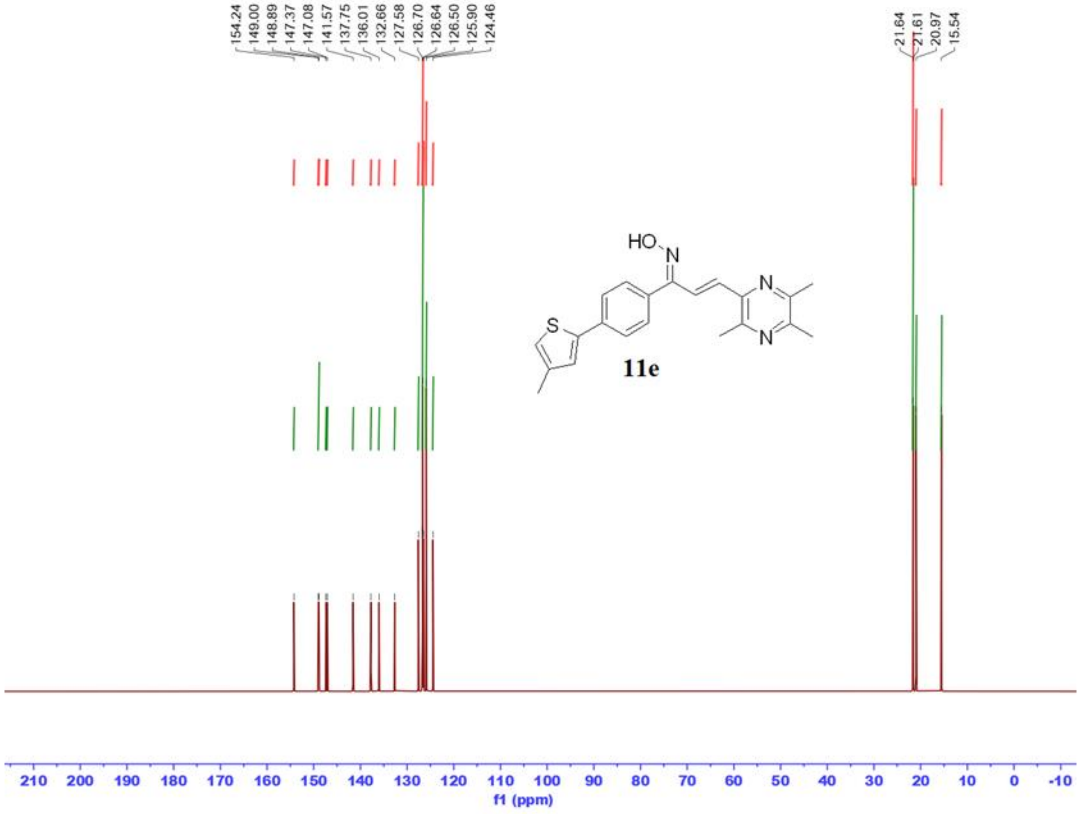

^{1}H NMR spectrum of **11f**

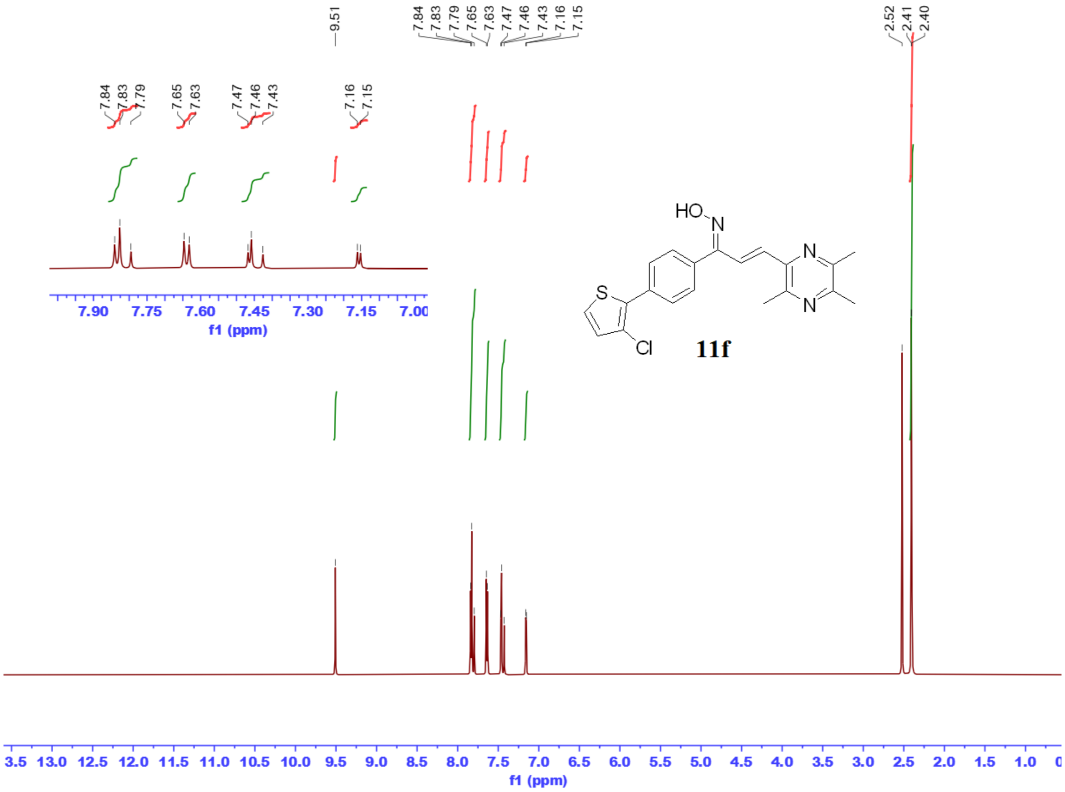

^{13}C NMR spectrum of **11f**

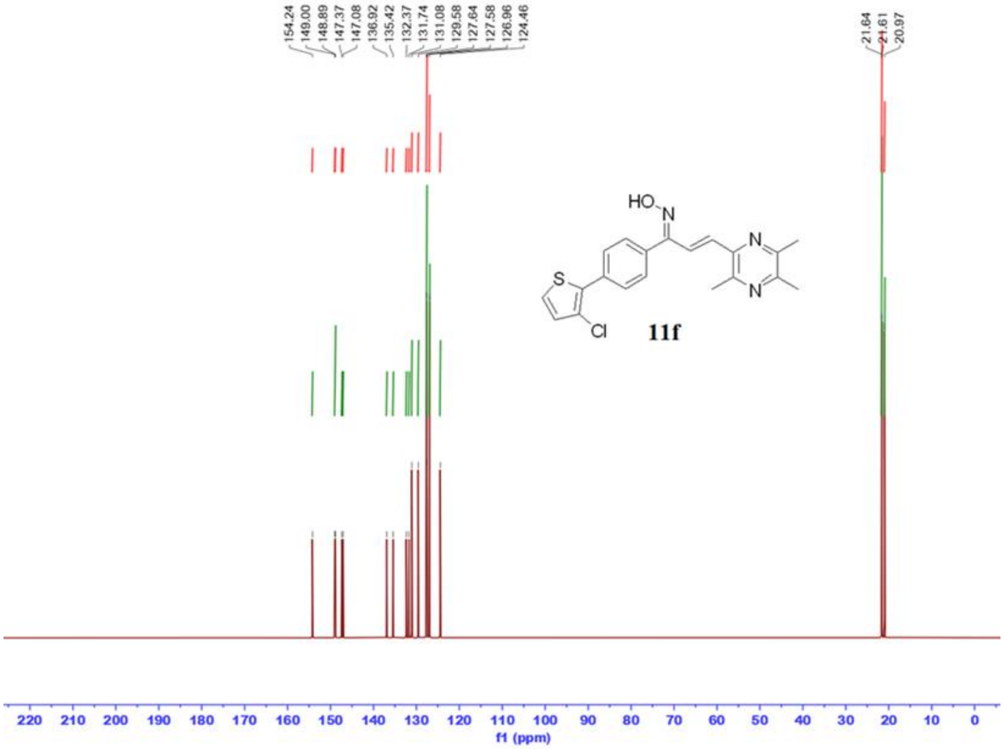

^{1}H NMR spectrum of **11g**

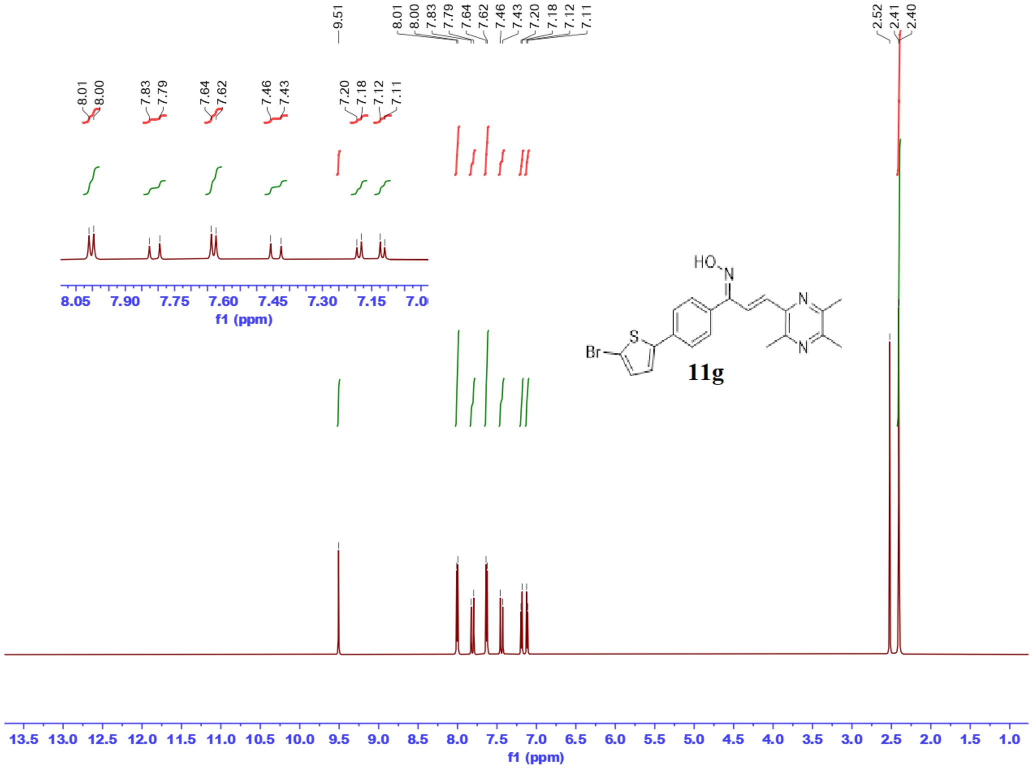

^{13}C NMR spectrum of **11g**

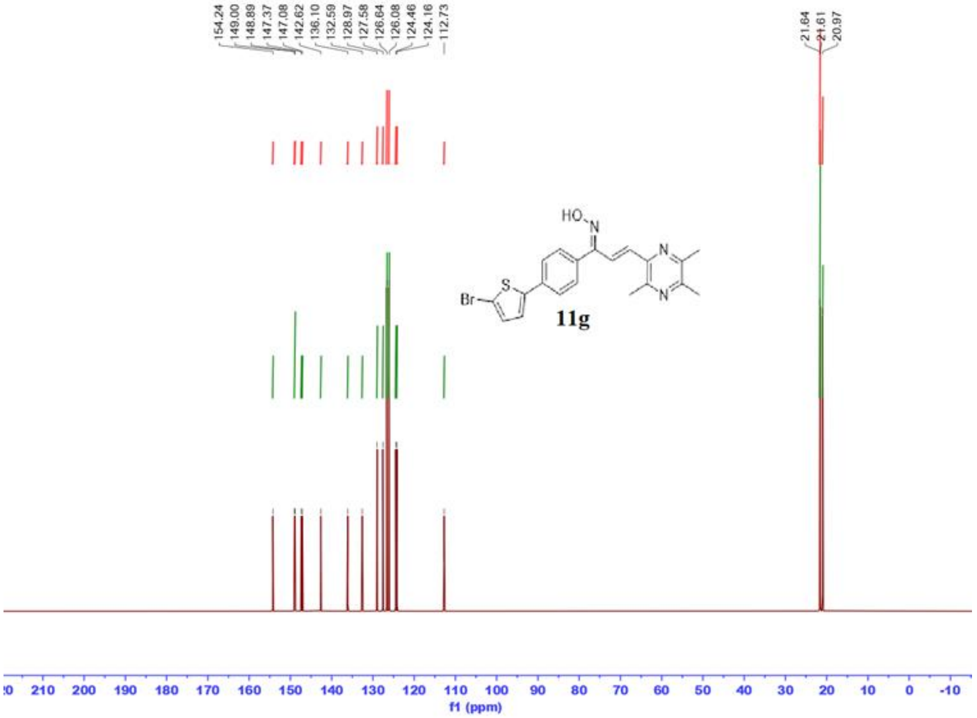

Supplement: RA-012-D2RA01198K-s001 [file RA-012-D2RA01198K-s001.pdf]
